# Biomarkers of rheumatoid arthritis-associated interstitial lung disease: a systematic review and meta-analysis

**DOI:** 10.3389/fimmu.2024.1455346

**Published:** 2024-10-29

**Authors:** Luhan Guo, Jun Wang, Jiansheng Li, Jiaheng Yao, Hulei Zhao

**Affiliations:** ^1^ Department of Respiratory Diseases, The First Affiliated Hospital of Henan University of Chinese Medicine, Zhengzhou, Henan, China; ^2^ The First Clinical Medical College of Henan University of Chinese Medicine, Henan, China; ^3^ Collaborative Innovation Center for Chinese Medicine and Respiratory Diseases Co-constructed by Henan Province & Education Ministry of P.R, Zhengzhou, Henan, China

**Keywords:** rheumatoid arthritis, interstitial lung disease, biomarkers, systematic review, meta-analysis

## Abstract

**Background:**

Interstitial Lung Disease (ILD) represents the most common extra-articular manifestation of Rheumatoid Arthritis (RA) and is a major cause of mortality. This study aims to identify and evaluate biomarkers associated with Rheumatoid Arthritis-Associated Interstitial Lung Disease (RA-ILD).

**Methods:**

We searched PubMed, Cochrane Library, EMBASE, and Web of Science databases for studies related to biomarkers of RA-ILD up until October 7, 2023. The Newcastle-Ottawa Scale (NOS) and standards recommended by the Agency for Healthcare Research and Quality (AHRQ) were used for quality assessment, and meta-analysis was conducted using Stata18.0 software.

**Results:**

A total of 98 articles were assessed for quality, 48 of which were included in the meta-analysis. 83 studies were of high quality, and 15 were of moderate quality. The meta-analysis showed significant differences in biomarkers such as C-Reactive Protein (CRP), Erythrocyte Sedimentation Rate (ESR), Anti-Cyclic Citrullinated Peptide (anti-CCP) antibody, Rheumatoid Factor (RF), Krebs von den Lungen-6 (KL-6), Surfactant Protein D (SP-D), Carcinoembryonic Antigen (CEA), Carbohydrate Antigen 19-9 (CA19-9), Matrix Metalloproteinase-7 (MMP-7), C-X-C Motif Chemokine Ligand 10 (CXCL-10), and Neutrophil-to-Lymphocyte Ratio (NLR) between RA-ILD patients and RA patients. However, Platelet-to-Lymphocyte Ratio [Platelet-to-Lymphocyte Ratio (PLR)], Cancer Antigen 125 [Cancer Antigen 125 (CA-125)], and Cancer Antigen 153 [Cancer Antigen 153 (CA-153)] did not show significant differences between the two groups. KL-6, MMP-7, and Human Epididymis Protein 4 (HE4) are negatively correlated with lung function, and KL-6 is associated with the prognosis of RA-ILD.

**Conclusions:**

Biomarkers hold promising clinical value for prediction, diagnosis, severity assessment, and prognosis evaluation in RA-ILD. However, these findings need to be validated through multicenter, large-sample, prospective cohort studies.

**Systematic review registration:**

https://www.crd.york.ac.uk/prospero/, identifier CRD42023448372.

## 1 Introduction

Rheumatoid Arthritis (RA) is a systemic autoimmune disease characterized by progressive joint destruction, with a prevalence rate of approximately 0.5% to 1.0% in developed countries (1). Pulmonary involvement is the most common extra-articular manifestation of RA and the leading cause of death among RA patients (2, 3). Currently, the diagnosis of Rheumatoid Arthritis-Associated Interstitial Lung Disease (RA-ILD) requires assessment in collaboration with radiology, pathology, rheumatology, and pulmonology experts (4). However, there is a lack of tools for early diagnosis and effective prediction of disease progression. Biomarkers have promising applications in RA-ILD, offering important information about disease activity, progression, and response to treatment. They hold potential value for the management of RA-ILD, yet the diagnostic utility of almost all reported biomarkers, as well as their predictive efficacy for severity and prognosis, has not been well validated (5). No study has comprehensively summarized these biomarkers and quantitatively assessed their relationship with RA-ILD. Therefore, this study aims to conduct a systematic review and meta-analysis to screen for biomarkers related to RA-ILD.

## 2 Manuscript formatting

### 2.1 Methods

This study was registered on PROSPERO (https://www.crd.york.ac.uk/prospero/, CRD42023448372) and conducted according to the Preferred Reporting Items for Systematic Reviews and Meta-Analyses (PRISMA) guidelines (6).

#### 2.1.1 Search strategy

We searched PubMed, the Cochrane Library, EMBASE, and Web of Science databases for relevant literature published up to October 7, 2023, using the terms “Rheumatoid Arthritis,” “Interstitial Lung Disease,” and “Biomarkers.” We also manually searched the reference lists of eligible studies and related review articles to identify additional reports. The detailed search strategy is provided in **Supplementary Data Sheet 1**.

#### 2.1.2 Study selection

Two researchers (Guo/Yao) independently screened the titles and abstracts of all retrieved articles to select studies that matched our criteria. Disagreements were resolved through discussion or by a third reviewer (Zhao). Inclusion criteria were: (1) Cohort studies, case-control studies, cross-sectional studies; (2) Patients diagnosed with RA-ILD; (3) Studies reporting on biomarkers for the prediction, diagnosis, severity assessment, and prognosis evaluation of RA-ILD. Exclusion criteria included: (1) for duplicate publications, only the study with the most comprehensive data was selected; (2) incomplete data or missing target indicators; (3) review articles, letters, conference records, editorials, and case reports.

#### 2.1.3 Data extraction and quality assessment

Two researchers (Guo/Yao) independently extracted data from the included studies. Extracted data included the general study characteristics (first author’s name, publication year, study location, study design), information about the studied population (sample size, number of men and women, mean age, diagnostic criteria), and information on the outcomes in the study (biomarker types, measurement methods, etc). If detailed information was not available, we contacted the authors via email to obtain data.Study quality was assessed using the Newcastle-Ottawa Scale (NOS) for cohort and case-control studies. A score of 0-3 was considered low quality, 4-6 medium quality, and 7-9 high quality. For cross-sectional studies, quality was evaluated according to standards recommended by the Agency for Healthcare Research and Quality (AHRQ), with scores of 0-3 indicating low quality, 4-7 medium quality, and 8-11 high quality. Both researchers collaborated to assess study quality and reached a consensus through discussion.

#### 2.1.4 Statistical analysis

Statistical analysis was performed using STATA 18.0 software. When a specific biomarker is represented by more than two independent studies, we conduct a meta-analysis. For the Pearson correlation coefficient, the inverse variance method was used to calculate the pooled correlation coefficients between the biomarkers and RA-ILD, along with their corresponding 95% confidence intervals (CI). To obtain variance-stabilized correlation coefficients, Pearson’s correlation coefficients were transformed into Fisher’s Z-scores before pooling the estimates (7). For continuous variables, the overall effect size was calculated using mean difference (MD) and CI. Median and interquartile ranges (IQRs) were converted to estimated means and standard deviations (8–10). The adjusted hazard ratio (HR) and 95% confidence interval (CI) were used to assess the prognosis of RA-ILD. Heterogeneity among studies was assessed using the Q test, and the inconsistency index (I^2^) was expressed as a percentage. A P-value ≤ 0.05 was considered statistically significant (11). A fixed-effect model was generally used to analyze trials with strong homogeneity (I^2^ ≤ 50%, P > 0.1). In cases of statistical heterogeneity (I^2^ > 50%, P < 0.1), a random-effects model was applied, followed by sensitivity. Subgroup analysis was conducted based on region and age (according to World Health Organization (WHO) standards, age ≥60 years was classified as elderly, and age <60 years was classified as non-elderly). Meta-regression analysis was also conducted to assess the impact of other potential confounding factors. Publication bias was evaluated using Egger’s test when five or more studies were available for meta-analysis. If publication bias was detected, the trim-and-fill method was used to assess funnel plot asymmetry (12).

### 2.2 Results

#### 2.2.1 Study selection

**Figure 1:**
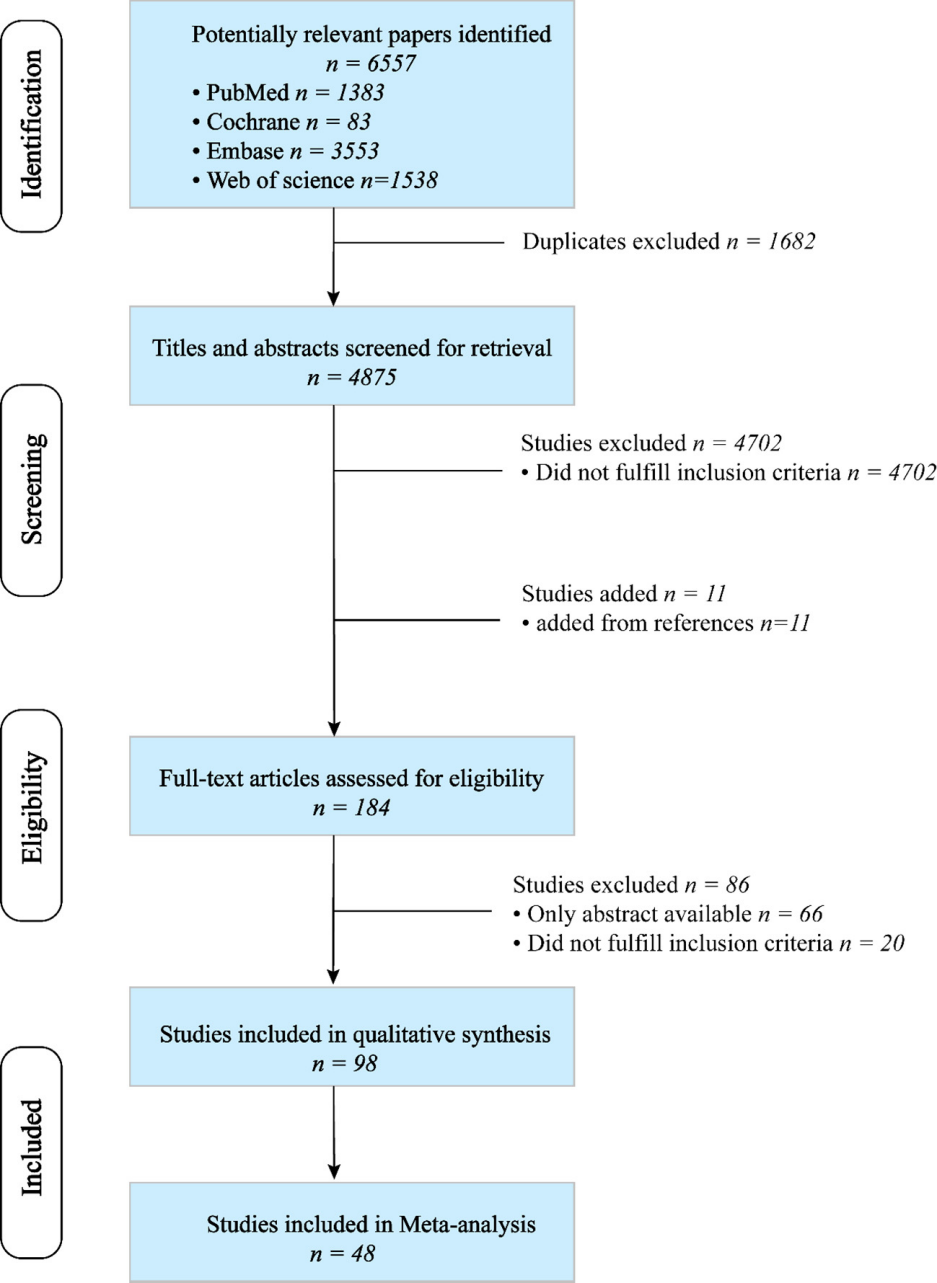
Study selection flowchart.

A total of 6,557 articles were retrieved through a search of four databases. After removing duplicates, 4,875 articles were identified. The titles and abstracts were screened, and the reference lists of relevant review articles were reviewed to identify potentially suitable articles. Among 184 articles reviewed, 86 were considered ineligible. Finally, 98 publications met the inclusion criteria (13–110), of which 48 were included in the meta-analysis (13–60). The detailed process of study selection is illustrated in **Figure 1**.

#### 2.2.2 Study characteristics

The analysis included studies from 13 different countries, primarily employing a case-control study design. The majority of the studies were conducted in China (n=30), followed by Japan (n=18), the United States (n=17), Spain (n=6), South Korea (n=5), Egypt (n=5), Italy (n=4), the United Kingdom (n=2), Turkey (n=2), France (n=2), Mexico (n=2), Sweden (n=1), Germany (n=1), and Saudi Arabia (n=1) with two studies spanning multiple countries. The diagnosis of Rheumatoid Arthritis (RA) generally adhered to the 1987 American College of Rheumatology (ACR) criteria or the 2010 ACR/European League Against Rheumatism (EULAR) criteria. Most studies assessing Interstitial Lung Disease (ILD) utilized High-Resolution Computed Tomography (HRCT), with a few also conducting surgical lung biopsies. The methodological quality of the included studies was evaluated using the Newcastle-Ottawa Scale (NOS) or the standards set by the Agency for Healthcare Research and Quality (AHRQ). Among them, 83 studies were of high quality, and 15 were of medium quality. More details on the characteristics of the study subjects can be found in **Supplementary Data Sheet 1**.

#### 2.2.3 Biomarkers of RA-ILD

Identified biomarkers were classified into four types: prediction, diagnosis, severity assessment, and prognosis evaluation. There were 11 biomarkers for predicting the occurrence of RA-ILD, 85 for differentiating RA from RA-ILD, 17 for assessing the severity of RA-ILD, and 14 for evaluating prognosis. The specifics are provided in **Table 1**.

**Table 1:** Summary of biomarkers associated with RA-ILD.

Biomarker category	Biomarkers associated with RA-ILD
Prediction	Multiple studies: MUC5B mutations, FS-ACPA Single study: Anti-PAD3/4 antibodies, anti-CCP, RF, THBS2, TIMP1, POSTN, CD19, rs6578890 in PPFIBP2, fragmented gelsolins,
Diagnosis	Multiple studies: KL-6, MMP-7, SP-D, RF, HE4, anti-CCP, CRP, ESR, CEA, CA19-9, CA-125, CA-153, CXCL10 Single study: MCP-1, CCL2, SDF-1 α, IL-18, CHI3L1, IL-36α, IL-36γ, qPCR (T/S ratio), VCAM-1, MCP-1, ADMA, D-dimer, UA, SIRI, sPD-1, cDKK-1, CA, GL, PIP, MMP-1, MMP-2, MMP-9, IL-1RA, sCD40L, CXCL9, EPC, PD-L1, lncRNA(NR_002819,NR_038935,ENST00000603415,ENST00000560199), PLR, Wnt5a, aCEP-1, LOXL2, CCL18, IL-17, CXCL12, CCL5, FGF4, FGF7, hsa-miR-214-5p, hsa-miR-7-5p, PARC, Hsp90, TGF-β1, sCD25, POSTN, CD16+MO, ARG1, TYMS, MKI67, OLFM4, BIRC5, MS4A4A, CLEC12A, LINC02967, HLA-DRB1, HLA-DRB1 SE, HLA-DQB1, IL-33, Anti-CarP, Anti-MAA, T cell subsets, NK cell subsets, IL-1α autoantibodies, anti-ARS antibodies, iPF,DLCO, decanoic acid, morpholine, glycerol, proteomic profiling, CD20+B cells, CD138+plasma cells
Severity assessment	Multiple studies: KL-6, MMP-7, HE4 Single study: anti-CCP, UA, IL-13, IL-13 Rα1, IL-13 Rα2, SP-D, CEA, CA19-9, CA-125, Wnt5a, CXCL10, MMP-9, TIMP-1, coinhibitory molecules on alveolar T cells
Prognosis evaluation	Multiple studies: KL-6 Single study: PADI2(rs2057094,rs2076615), PADI4(rs1748033), IL-6, IL-18, IL-11, MMP-13, CXCL11, Wnt5a, monocytes, neutrophils, KL-6 change rate over years, RF,HRCT

Anti-PAD3/4 antibodies, Anti-PAD3/4 antibodies; anti-CCP, anti-cyclic citrullinated peptide antibody; RF, rheumatoid factor; KL-6, krebs von den lungen-6; MMP-7, matrix metalloproteinase-7; SP-D, surfactant protein D; HE4, human epididymis protein 4; CRP, C-reactive protein; ESR, erythrocyte sedimentation rate; CEA, carcinoembryonic antigen; CA19-9, carbohydrate antigen 19-9; CA-125, cancer antigen 125; CA-153, cancer antigen 153; CXCL10, C-X-C motif chemokine ligand 10; MCP-1, monocyte chemoattractant protein-1; CCL2, chemokine (C-C motif) ligand 2; SDF-1 α, stromal cell-derived factor 1 alpha; IL-18, interleukin-18; CHI3L1, chitinase-3-like protein; IL-36α, interleukin-36 alpha; IL-36γ, Interleukin-36 alpha; qPCR (T/S ratio), quantitative polymerase chain reaction (T/S ratio); VCAM-1, vascular cell adhesion molecule-1; MCP-1, monocyte chemoattractant protein-1; ADMA, asymmetric dimethylarginine; UA, uric acid; SIRI, Simplified Index for the Robustness of Inflammation; sPD-1, soluble programmed cell death protein 1; cDKK-1, Circulating Dickkopf-1; CA, caprylic acid; GL, glycerol; PIP, piperidine; MMP-1, matrix metalloproteinase-1; MMP-2, matrix metalloproteinase-2; MMP-9, matrix metalloproteinase-9; IL-1RA, interleukin-1 receptor antagonists; sCD40L, soluble CD40 ligand; CXCL9, C-X-C motif chemokine ligand 9, EPC, endothelial progenitor cells; PD-L1, programmed cell death ligand 1; PLR, platelet-to-lymphocyte ratio; Wnt5a, non-canonical Wnt signaling representative ligand Wnt5a; aCEP-1, anti-C-terminal epitope antibody 1; LOXL2, lysyl oxidase-like 2; CCL18, C-C motif chemokine ligand 18; IL-17, interleukin-17; CXCL12, C-X-C motif chemokine ligand 12; CCL5, C-C motif chemokine ligand 5; FGF4, fibroblast growth factor 4; FGF7, fibroblast growth factor 7; PARC, pulmonary and activation-regulated chemokine; Hsp90, heat shock protein 90; TGF-β1, transforming growth factor beta 1; sCD25, soluble CD25; POSTN, periostin; CD16+MO, CD16-positive monocytes; ARG1, arginase 1; TYMS, thymidylate synthase; SORT1, sortilin 1; MKI67, marker of proliferation Ki-67; OLFM4, Olfactomedin 4; BIRC5, baculoviral IAP repeat containing 5; MS4A4A, membrane-spanning 4-domains subfamily A member 4A; CLEC12A, C-type lectin domain family 12 member A; LINC02967, long intergenic non-protein coding RNA 2967; HLA-DRB1 SE, human leukocyte antigen class II DRB1 shared epitope; IL-33, interleukin-33; Anti-CarP, anti-carbamylated protein antibodies; Anti-MAA, malondialdehyde-acetaldehyde; IL-1α autoantibodies, interleukin-1 alpha autoantibodies; anti-ARS antibodies, anti-aminoacyl-tRNA synthetase antibodie; iPF, immature platelet fraction; IL-13 Rα1, interleukin-13 receptor alpha 1; TIMP-1, tissue inhibitor of metalloproteinases; FS-ACPA, fine-specificity anti-citrullinated protein antibodies; THBS2, thrombospondin-2; TIMP1, tissue inhibitor of metalloproteinases 1; CD19, cluster of differentiation 19; DLCO, diffusing capacity of the lungs for carbon monoxide; HRCT, high-resolution computed tomography.

#### 2.2.4 Meta-analysis

##### 2.2.4.1 Biomarkers for the diagnosis of RA-ILD

Biomarkers identified in two or more eligible studies were included in the meta-analysis, including C-Reactive Protein (CRP), Erythrocyte Sedimentation Rate (ESR), Rheumatoid Factor (RF), Anti-Cyclic Citrullinated Peptide (Anti-CCP) antibody, Krebs von den Lungen-6 (KL-6), Surfactant Protein D (SP-D), Carcinoembryonic Antigen (CEA), Carbohydrate Antigen 19-9 (CA19-9), Cancer Antigen 125 (CA-125), Cancer Antigen 153 (CA-153), Matrix Metalloproteinase-7 (MMP-7), C-X-C Motif Chemokine Ligand 10 (CXCL-10), Platelet to Lymphocyte Ratio (PLR), and Neutrophil to Lymphocyte Ratio (NLR).

###### 2.2.4.1.1 CRP

**Figure 2:**
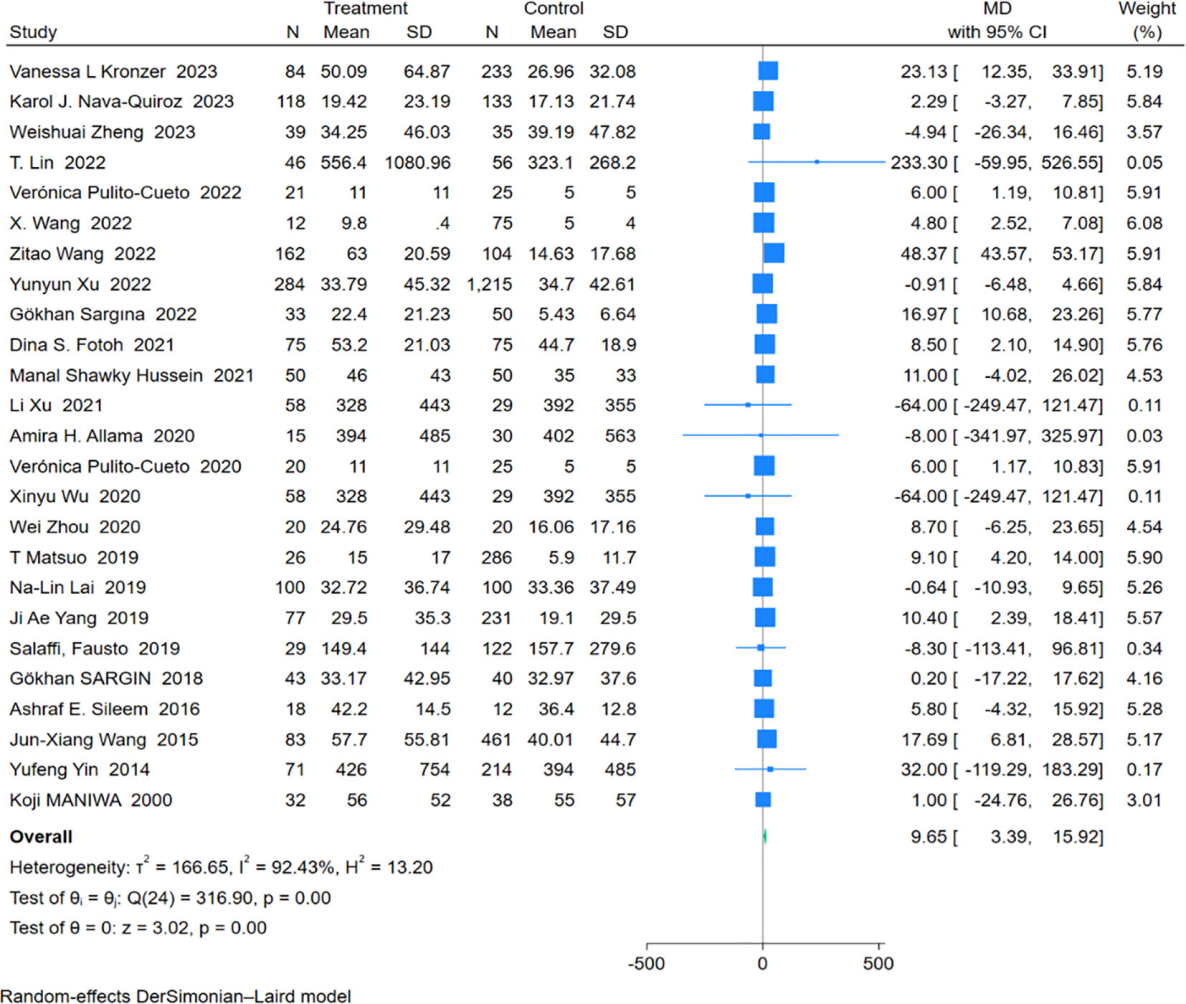
Forest plots of association between CRP and RA-ILD.

An analysis of 25 studies including 1,574 RA-ILD patients and 3,688 RA patients was conducted to compare CRP levels. The pooled effect size showed significantly higher CRP concentrations in the RA-ILD group compared to the RA group (MD = 9.65; 95% CI: 3.39-15.92; P < 0.001). There was high heterogeneity among the studies (I^2^ = 92.43%, P < 0.001). The forest plot of the pooled analysis is shown in **Figure 2**. Subgroup analysis by region showed significant differences in CRP concentrations between RA-ILD and RA groups in Asia (MD = 9.98; 95% CI: 0.06-19.89; P = 0.05), Europe (MD = 5.98; 95% CI: 2.58-9.39; P < 0.001), and Africa (MD = 8.10; 95% CI: 3.02-13.19; P < 0.001), but not in the Americas (MD = 12.18; 95% CI: -8.22-32.57; P = 0.24). Subgroup analysis by age showed significant differences in CRP concentrations between RA-ILD and RA groups among older adults (aged ≥60 years) (MD = 6.39; 95% CI: 3.19-9.59; P < 0.001), but not among younger individuals (aged <60 years) (MD = 16.94; 95% CI: -0.72-34.60; P = 0.06) (**Table 2**). Meta-regression analysis was conducted to identify sources of heterogeneity, revealing that total sample size (P=0.865) and gender ratio (P=0.192) were not sources of heterogeneity. Specific charts are available in **Supplementary Data Sheet 1**.

**Table 2:** Subgroup analysis aggregated data.

Biomarker	Subgroup	No. of studies	No. of patients	Pooled estimates		
				MD (95% CI)	P-value	I^2^ (%)
CRP	Region					
	Asia	17	4172	9.98 (0.06-19.89)	0.05	94.61
	America	2	568	12.18 (-8.22-32.57)	0.24	91.19
	Europe	3	242	5.98 (2.58-9.39)	<0.01	0.00
	Africa	3	280	8.10 (3.02-13.19)	<0.01	0.00
	Age					
	Elederly	17	1985	6.39 (3.19-9.59)	<0.01	58.52
	Non-elederly	8	3277	16.94 (-0.72-34.60)	0.06	95.31
ESR	Region					
	Asia	18	4486	6.31 (1.49-11.12)	<0.01	82.59
	America	2	883	8.94 (-8.39-26.27)	0.31	95.49
	Europe	3	242	3.88 (-4.12-11.88)	0.34	35.12
	Africa	2	250	10.40 (2.99-17.82)	0.01	43.36
	Age					
	Elederly	18	4223	5.60 (1.76-9.44)	<0.01	74.68
	Non-elederly	7	1638	9.95 (2.86-17.04)	0.01	79.98
Anti-CCP	Region					
	Asia	16	2804	72.79 (39.00-106.58)	<0.01	86.48
	America	6	645	0.37 (-83.92-84.66)	0.99	92.05
	Europe	1	460	30.43 (-14.84-75.70)	0.19	/
	Africa	2	250	94.11 (47.77-140.44)	<0.01	0.00
	Age					
	Elederly	15	2387	51.27 (8.51-94.04)	0.02	88.09
	Non-elederly	10	1772	71.53 (25.61-117.46)	<0.01	86.18
RF	Region					
	Asia	20	4477	194.62 (128.88-260.35)	<0.01	91.50
	America	4	217	194.50 (63.94-325.07)	<0.01	0.00
	Europe	2	330	61.47 (22.79-100.14)	<0.01	0.00
	Africa	2	250	19.07 (4.06-34.08)	0.01	0.00
	Age					
	Elederly	18	1691	109.76 (69.01-150.51)	<0.01	70.70
	Non-elederly	10	3583	215.63 (96.15-335.10)	<0.01	97.34
KL-6	Region					
	Asia	6	1944	534.53 (321.74-747.31)	<0.01	93.34
	Europe, Asia	1	147	320.00 (282.83-357.17)	<0.01	/
	Africa	2	250	790.38 (402.80-715.60)	<0.01	0.00
	Age					
	Elederly	7	1925	515.05 (363.42-666.68)	<0.01	93.09
	Non-elederly	2	416	663.26 (395.90-930.62)	<0.01	84.73
SP-D	Region					
	Asia	4	1366	89.59 (78.92-100.27)	<0.01	0.00
	America	1	136	8.87 (2.67-15.07)	0.01	/
	Europe, Asia	1	147	6.15 (5.57-6.73)	<0.01	/
	Africa	2	130	158.02 (117.76-198.29)	<0.01	19.36

###### 2.2.4.1.2 ESR

**Figure 3:**
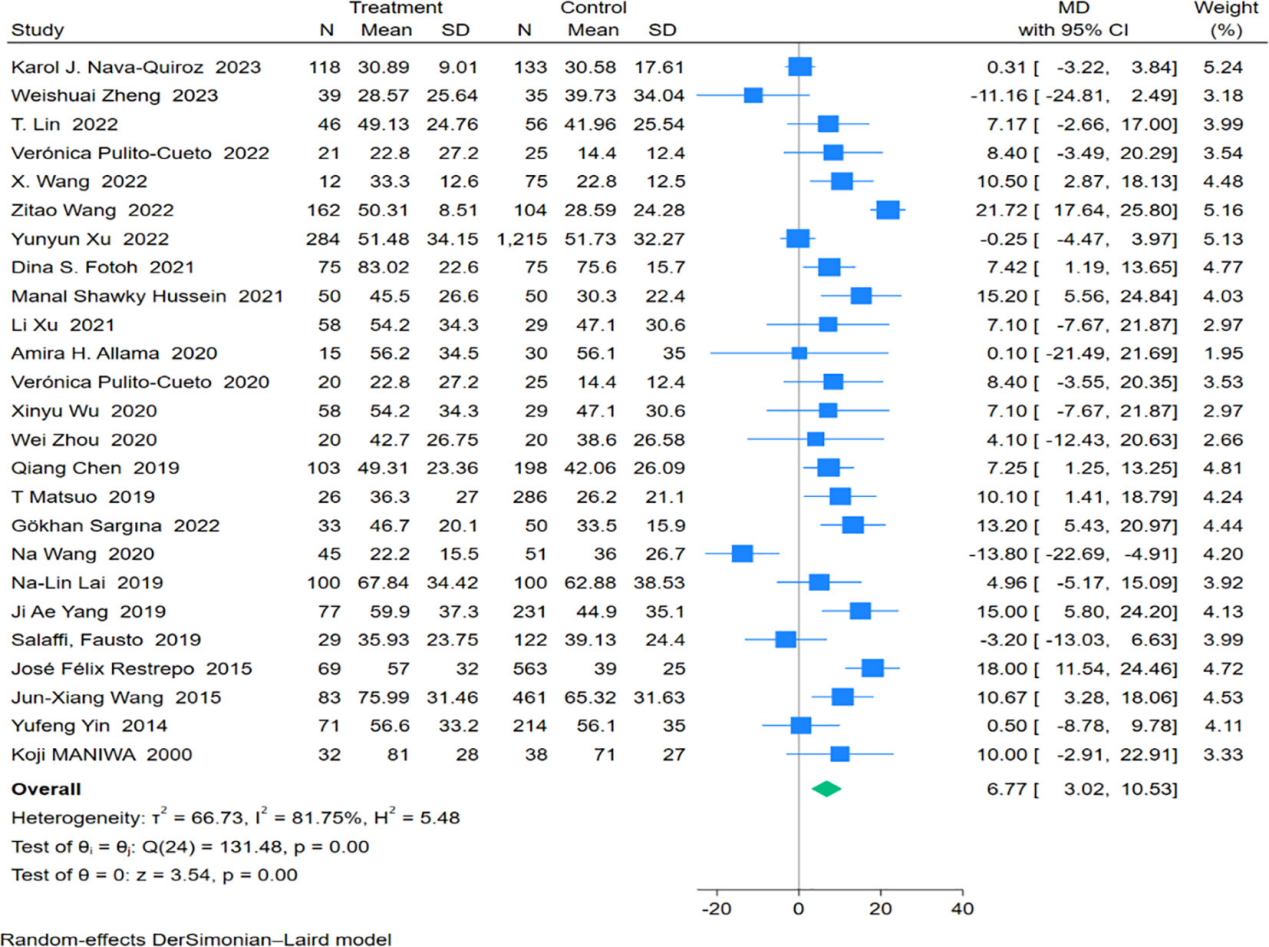
Forest plots of association between ESR and RA-ILD.

Twenty-five studies involving 1,646 patients with Rheumatoid Arthritis-Associated Interstitial Lung Disease (RA-ILD) and 4,215 patients with RA were analyzed. The pooled effect size indicated that ESR levels were significantly higher in the RA-ILD group compared to the RA group (Mean Difference (MD) = 6.77; 95% Confidence Interval (CI): 3.02-10.53; P < 0.001). There was substantial heterogeneity among the studies (I^2^ = 81.75%, P<0.001). The forest plot of the pooled analysis is shown in **Figure 3**. Subgroup analysis by region revealed that ESR levels were significantly higher in RA-ILD groups in Asia (MD = 6.31; 95% CI: 1.49-11.12; P = 0.01) and Africa (MD = 10.40; 95% CI: 2.99-17.82; P = 0.01), with no significant difference in the Americas (MD = 8.94; 95% CI: -8.39-26.27; P = 0.31) and Europe (MD = 3.88; 95% CI: -4.12-11.88; P = 0.34). Age-based subgroup analysis showed that both older (MD = 5.60; 95% CI: 1.76-9.44; P < 0.001) and younger individuals (MD = 9.95; 95% CI: 2.86-17.04; P < 0.001) in the RA-ILD group had significantly higher ESR levels compared to the RA group. (**Table 2**) Meta-regression analysis indicated that neither total sample size (P=0.996) nor gender ratio (P=0.538) were sources of heterogeneity. Specific charts are available in **Supplementary Data Sheet 1**.

###### 2.2.4.1.3 Anti-CCP antibody

**Figure 4:**
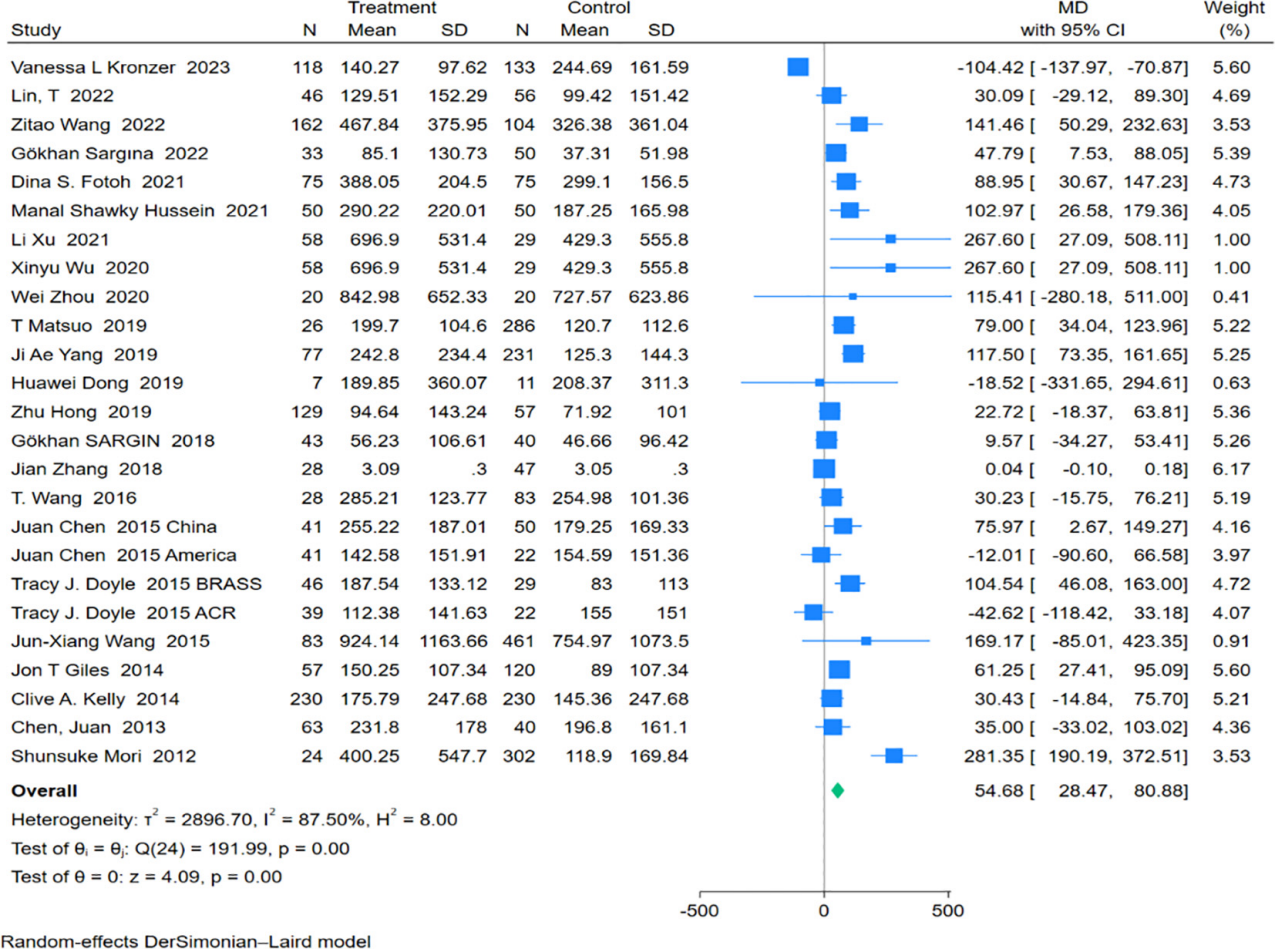
Forest plots of association between Anti-CCP Antibody and RA-ILD.

Twenty-five studies encompassing 1,582 RA-ILD patients and 2,577 RA patients were analyzed for anti-CCP antibody levels. The pooled effect size showed significantly higher levels of anti-CCP antibodies in the RA-ILD group compared to the RA group (MD = 54.68; 95% CI: 28.47-80.88; P < 0.001), with substantial heterogeneity observed (I^2^ = 87.50%, P<0.001). The forest plot of the pooled analysis is illustrated in **Figure 4**. Subgroup analyses by region indicated significant differences in anti-CCP antibody levels between RA-ILD and RA groups in Asia (MD = 72.79; 95% CI: 39.00-106.58; P < 0.001) and Africa (MD = 94.11; 95% CI: 47.77-140.44; P < 0.001), with no significant differences observed in the Americas (MD = 0.37; 95% CI: -83.92-84.66; P = 0.99) and Europe (MD = 30.43; 95% CI: -14.84-75.70; P = 0.19). Age-based subgroup analysis demonstrated significant differences in both older (MD = 51.27; 95% CI: 8.51-94.04; P =0.02) and younger participants (MD = 71.53; 95% CI: 25.61-117.46; P <0.001) in the RA-ILD group compared to the RA group. (**Table 2**) Meta-regression analysis showed that neither total sample size (P=0.296) nor gender ratio (P=0.722) were sources of heterogeneity. Detailed charts are included in the **Supplementary Data Sheet 1**.

###### 2.2.4.1.4 RF

**Figure 5:**
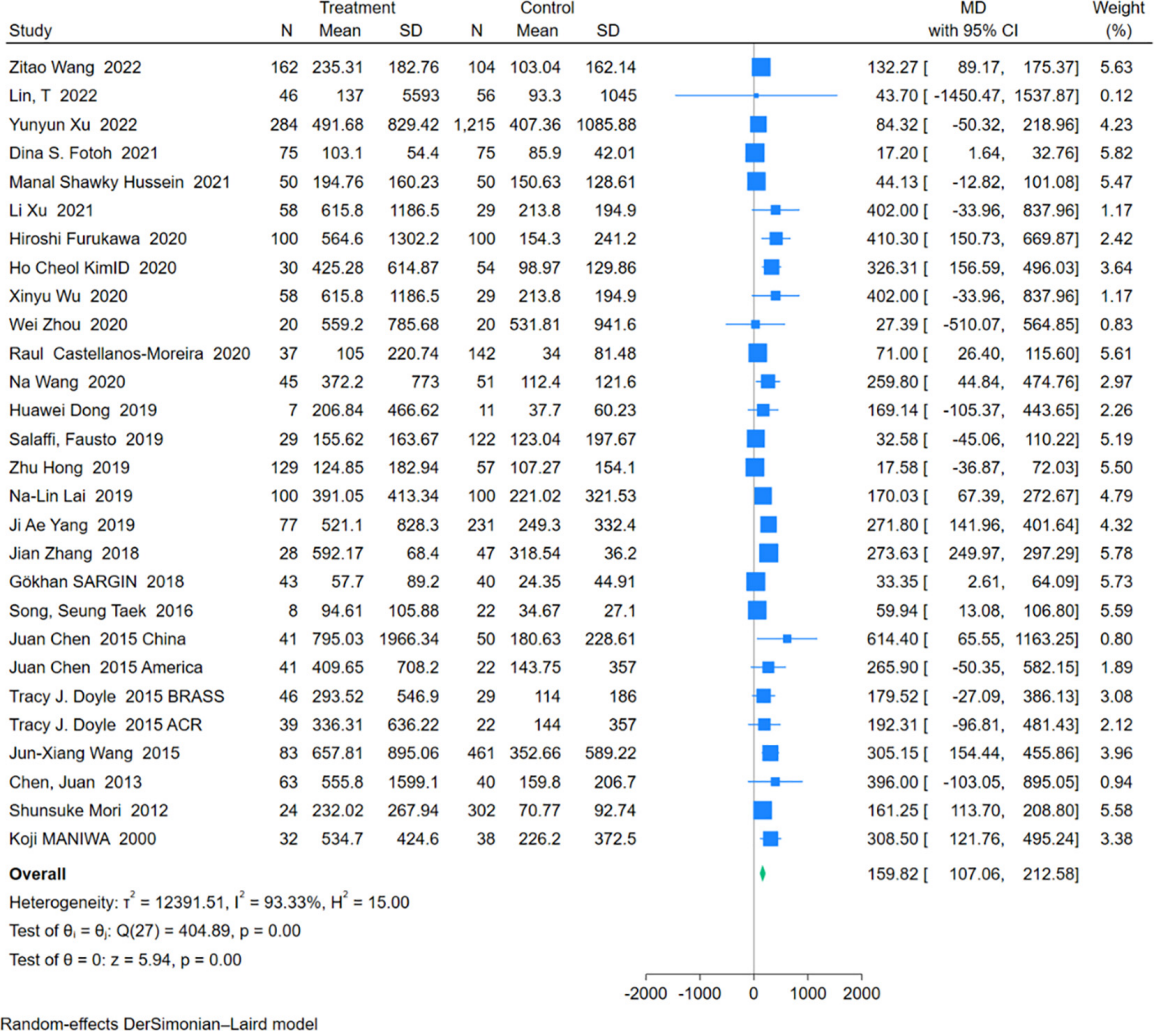
Forest plots of association between RF and RA-ILD.

An analysis of 28 studies including 1,755 RA-ILD patients and 3,519 RA patients was conducted to compare RF levels. The pooled effect size indicated significantly higher RF levels in the RA-ILD group compared to the RA group (MD = 159.82; 95% CI: 107.06-212.58; P < 0.001), with high heterogeneity (I^2^ = 93.33%, P<0.001). The forest plot of the pooled analysis is shown in **Figure 5**. Subgroup analysis by region showed significant differences in RF levels in Asia (MD = 194.62; 95% CI: 128.88-260.35; P < 0.001), the Americas (MD = 194.50; 95% CI: 63.94-325.07; P < 0.001), Europe (MD = 61.47; 95% CI: 22.79-100.14; P < 0.001), and Africa (MD = 19.07; 95% CI: 4.06-34.08; P =0.01) for the RA-ILD group compared to the RA group. Age-based subgroup analysis demonstrated significant differences in both older (MD = 109.76; 95% CI: 69.01-150.51; P < 0.001) and younger participants (MD = 215.63; 95% CI: 96.15-335.10; P < 0.001) in the RA-ILD group compared to the RA group. (**Table 2**) Meta-regression analysis showed that neither total sample size (P=0.296) nor gender ratio (P=0.722) were sources of heterogeneity. Detailed charts are included in the **Supplementary Data Sheet 1**.

###### 2.2.4.1.5 KL-6

**Figure 6:**
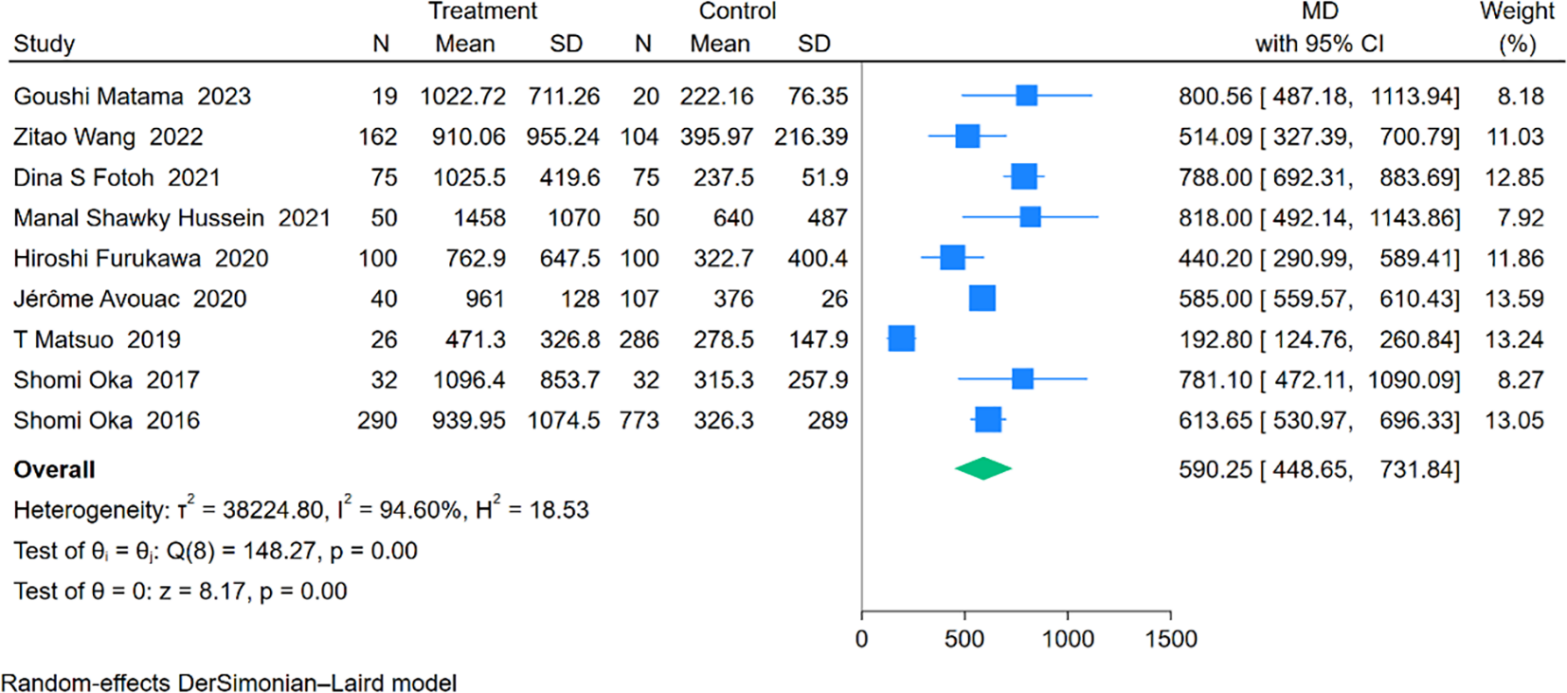
Forest plots of association between KL-6 and RA-ILD.

An analysis involving 9 studies compared KL-6 levels between 794 patients with RA-ILD and 1,547 patients with RA. The pooled effect size demonstrated significantly higher KL-6 concentrations in the RA-ILD group compared to the RA group (MD = 590.25; 95% CI: 448.65-731.84; P < 0.001), with substantial heterogeneity observed (I^2^ = 94.60%, P < 0.001). The forest plot of the pooled analysis is shown in **Figure 6**. Subgroup analyses by region showed significant differences, with reports from Africa (MD = 790.38; CI: 698.57-882.19; P < 0.001) and Asia (MD = 534.53; CI: 321.74-747.31; P < 0.001), including a study covering both Europe and Asia reporting an MD of 585.00 (CI: 559.57-610.43; P < 0.001). All regions reported significantly higher KL-6 levels in RA-ILD patients compared to RA patients. Age-based subgroup analysis revealed significant differences for both older (MD = 570.00; 95% CI: 401.96-738.03; P < 0.001) and younger individuals (MD = 663.26; 95% CI: 395.90-930.62; P < 0.001). (**Table 2**) Meta-regression indicates that the proportion of males is the source of heterogeneity(P=0.002). Detailed charts are available in the **Supplementary Data Sheet 1**.

###### 2.2.4.1.6 SP-D

**Figure 7:**
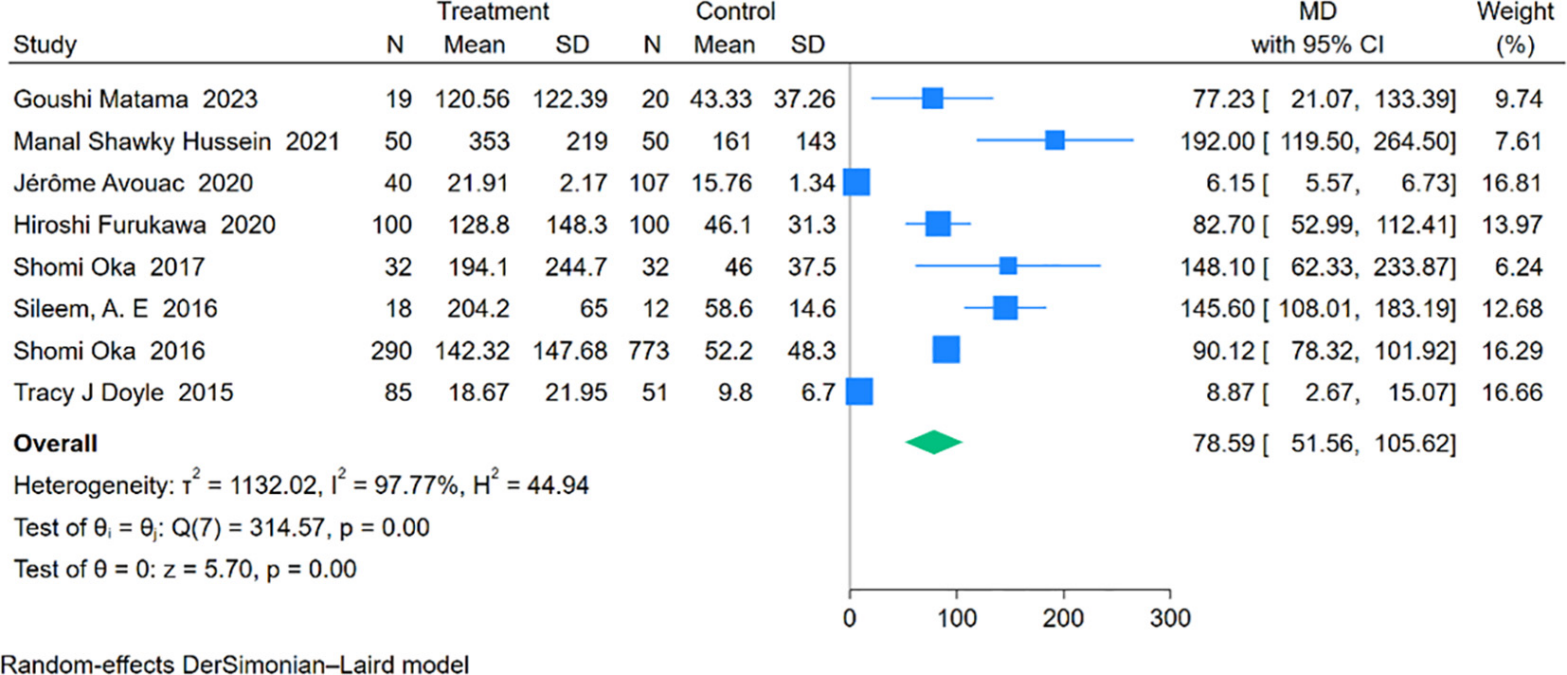
Forest plots of association between SP-D and RA-ILD.

Eight studies involving 634 RA-ILD patients and 1,145 RA patients were analyzed for SP-D concentrations. The pooled effect size indicated significantly higher SP-D levels in the RA-ILD group compared to the RA group (MD = 78.59; 95% CI: 51.56-105.62; P < 0.001), with notable heterogeneity (I^2^ = 97.77%, P < 0.001). The forest plot of the pooled analysis is shown in **Figure 7**. Subgroup analysis by region revealed significant differences in SP-D levels in Asia (MD = 89.59; 95% CI: 78.92-100.27; P < 0.001), the Americas (MD = 8.87; 95% CI: 2.67-15.07; P = 0.01), and Africa (MD = 158.02; 95% CI: 117.76-198.29; P < 0.001), with a study covering both Asia and Europe reporting an MD of 6.15 (CI: 5.57-6.73; P < 0.001). (**Table 2**) Detailed charts are available in the **Supplementary Data Sheet 1**.

###### 2.2.4.1.7 MMP-7

Three cohort comprising 167 RA-ILD patients and 123 RA patients reported plasma levels of MMP-7. The pooled effect size showed significantly higher MMP-7 concentrations in the RA-ILD group compared to the RA group (MD = 1.00; 95% CI: 0.74-1.25; P < 0.001), with low heterogeneity (I^2^ = 33.82%, P = 0.22). The forest plot of the pooled analysis is included in the **Supplementary Material**.

###### 2.2.4.1.8 Tumor markers

Four studies involving 383 patients with RA-ILD and 1,385 patients with RA were analyzed for CEA and CA19-9 concentrations. The pooled effect size demonstrated that CEA (MD = 1.66; 95% CI: 0.28-3.03; P = 0.02) and CA19-9 (MD = 21.92; 95% CI: 10.60-33.24; P < 0.001) levels were significantly higher in the RA-ILD group compared to the RA group, with high heterogeneity observed (CEA: I^2^ = 94.22%, P < 0.001; CA19-9: I^2^ = 94.74%, P < 0.001). The forest plot of the pooled analysis is included in the **Supplementary Material**. Three studies reported serum levels of CA-125 involving 355 RA-ILD patients and 1,338 RA patients. Compared to RA patients, there was no significant difference in CA-125 levels among RA-ILD patients (MD = 15.83; 95% CI: -7.60-39.26; P = 0.19), with high heterogeneity observed (I^2^ = 97.21%). Three studies reported serum levels of CA-153 involving 99 RA-ILD patients and 170 RA patients. No significant difference was observed in CA-153 levels between RA-ILD and RA patients (MD = 25.83; 95% CI: -4.45-56.11; P = 0.09), with high heterogeneity (I^2^ = 98.58%). Detailed charts are available in the **Supplementary Data Sheet 1**.

###### 2.2.4.1.9 Other biomarkers (CXCL-10, PLR, NLR)

Three cohort comprising 167 RA-ILD patients and 123 RA patients reported plasma levels of MMP-7. The pooled effect size showed significantly higher MMP-7 concentrations in the RA-ILD group compared to the RA group (MD = 1.00; 95% CI: 0.74-1.25; P < 0.001), with low heterogeneity (I^2^ = 33.82%, P = 0.22). The forest plot of the pooled analysis is included in the **Supplementary Material**. Three cohort reported serum levels of CXCL-10, involving 115 RA-ILD patients and 112 RA patients. A significant difference was observed in CXCL-10 levels between RA-ILD and RA patients (MD = 141.09; 95% CI: 81.24-200.94; P < 0.001), with low heterogeneity (I^2^ = 0.00%, P = 0.58). Two studies involving 387 RA-ILD patients and 1,413 RA patients analyzed the PLR and NLR. The pooled effect showed a significant difference in NLR between RA-ILD and RA patients (MD = 0.23; 95% CI: 0.12-0.35; P < 0.001), but not for PLR (MD = 0.13; 95% CI: -0.42-0.68; P = 0.64). NLR showed low heterogeneity (I^2^ = 61.16%, P = 0.11), while PLR showed high heterogeneity (I^2^ = 93.77%, P < 0.001). The forest plot of the pooled analysis is included in the **Supplementary Data Sheet 1**.

##### 2.2.4.2 Sensitivity analysis and publication bias

A sensitivity analysis, using a one-study-removed approach, indicated stability in the results for CRP, ESR, anti-CCP antibody, RF, KL-6, SP-D, CA-19-9, MMP-7, CXCL-10, but instability for CEA, CA-125, CA-153. Publication bias assessed using Egger’s test for biomarkers included in five or more studies showed no significant bias for CRP (P=0.71) and ESR (P=0.36); however, potential bias was suggested for anti-CCP antibody (P=0.0116), RF (P=0.0063), KL-6 (P=0.0402), and SP-D (P < 0.001), but trim-and-fill analyses indicated that the adjusted effect sizes remained consistent with the original findings. Detailed charts are available in the **Supplementary Data Sheet 1**.

#### 2.2.5 Biomarkers for the severity assessment of RA-ILD

Three biomarkers were involved in assessing the severity of RA-ILD, including KL-6, MMP-7, and HE4. Two studies involving a total of 237 RA-ILD patients analyzed the correlation of KL-6 with FVC and DLCO. The pooled effect showed a negative correlation between KL-6 and both FVC and DLCO (FVC: summary r = -0.26, 95% CI = -0.38 – -0.15, P < 0.001; DLCO: summary r = -0.33, 95% CI = -0.43 – -0.21, P < 0.001), with heterogeneity observed for FVC: I^2^ = 0.00%, P = 0.46, and DLCO: I^2^ = 76.40%, P = 0.04. Three studies involving a total of 243 RA-ILD patients analyzed the correlation between MMP-7 and DLCO. The pooled effect showed a negative correlation between MMP-7 and DLCO (summary r = -0.40, 95% CI = -0.49 – -0.28, P < 0.001), with low heterogeneity among studies (I^2^ = 0.00%, P = 0.72). Two studies involving a total of 94 RA-ILD patients analyzed the correlation between HE4 and FVC%. The pooled effect showed a negative correlation between HE4 and FVC% (summary r = -0.26, 95% CI = -0.44 – -0.06, P = 0.001), with low heterogeneity among studies (I^2^ = 0.00%, P = 0.42). Detailed charts are available in the **Supplementary Data Sheet 1**.

#### 2.2.6 Biomarkers for the prognostic assessment of RA-ILD

Two studies involving a total of 146 RA-ILD patients were analyzed for the prognosis of KL-6 in RA-ILD. The pooled effect size showed a correlation between KL-6 concentration and the prognosis of RA-ILD (HR = 3.01; 95% CI: 1.57-5.76; P < 0.001), with low heterogeneity among studies (I^2^ = 0.00%, P=0.98). Detailed charts are available in the **Supplementary Data Sheet 1**.

### 2.3 Discussion

This study systematically reviewed potential biomarkers in RA-ILD patients, categorizing them into four types: prediction, diagnosis, severity assessment, and prognosis evaluation. Quantitative research results were then meta-analyzed to explore the relationship between potential biomarkers and RA-ILD.

We have reviewed biomarkers that can be used to predict the occurrence of RA-ILD. However, due to limited relevant studies, we did not conduct a meta-analysis. Among them, mutations in the MUC5B gene have shown promising prospects for predicting the occurrence of RA-ILD. A study by P. A. Juge (45) found that MUC5B promoter variants are strong risk factors for the development of RA-ILD, especially in patients with radiological evidence of UIP pattern. However, the generalizability of these research findings awaits further exploration due to regional and ethnic limitations. Identifying biomarkers that can early detect the occurrence of ILD in RA patients is of great significance for early intervention and improving treatment efficacy in RA-ILD. Therefore, further research on biomarkers predicting the occurrence of RA-ILD is needed.

Our study found that several biomarkers, such as CHI3L1, IL-18, IL-36α, VCAM-1, MCP-1, CCL18, and PLR, showed promising potential in distinguishing RA from RA-ILD. However, due to the limited number of relevant studies, we did not conduct a meta-analysis. The study by Rui Yu et al. (64) demonstrated that serum CHI3L1 levels were elevated in RA-ILD patients, suggesting its potential as a non-invasive biomarker for RA-ILD detection. Interestingly, CHI3L1 also showed promise in differentiating RA-ILD from IPF. In both IPF and RA-ILD patients, MMP-7 and ACPA levels were significantly higher compared to RA patients, and serum CHI3L1 levels could differentiate IPF from RA-ILD (245.8 ± 180.2 ng/mL vs. 116.0 ± 58.3 ng/mL, p<0.001) and predict survival, offering potential value in identifying highly specific biomarkers. Similarly, the study by Verónica Pulito-Cueto et al. (22) found that serum VCAM-1, MCP-1, and ADMA levels were elevated in RA-ILD patients compared to those with RA and IPF, suggesting these biomarkers as useful tools for identifying ILD in RA patients and differentiating RA-ILD from IPF, aiding in the early diagnosis of RA-ILD. Although research on such biomarkers is still limited, they have shown significant value in distinguishing RA from RA-ILD and even RA-ILD from IPF. Their high specificity offers a valuable reference for future large-scale, multicenter clinical studies.

Our meta-analysis results show that in RA-ILD patients, CRP, ESR, anti-CCP antibodies, RF, KL-6, SP-D, CEA, CA19-9, MMP-7, CXCL-10, and NLR are all significantly higher than in RA patients, which indicates promising prospects for distinguishing between RA and RA-ILD patients. However, PLR, CA-125, and CA-153 did not show significant differences in pooled effect sizes between RA-ILD patients and RA patients, indicating the need for further research to explore their relationship with RA-ILD patients.

Previous findings (111, 112) have associated CRP, ESR, anti-CCP antibodies, and RF with RA-ILD. We included more studies and participants, and the pooled results were consistent with previous studies, making the results more reliable. However, our subgroup analysis indicates that biomarker levels vary by region and age among RA-ILD patients. Significant differences in ESR and anti-CCP antibody concentrations were observed between RA-ILD and RA patients in Asia and Africa but not in the Americas and Europe. CRP levels were significantly higher in RA-ILD patients in Asia, Europe, and Africa, but not in the Americas. RF concentrations were significantly higher in RA-ILD patients across all regions, suggesting its potential utility. Nevertheless, the subgroup analysis for the Americas and Europe should be considered cautiously due to the limited number of related studies. These biomarkers’ performance may be influenced by factors such as environmental conditions, genetics, ethnicity, lifestyle, and differences in detection methods across regions. In Asian and African populations, certain genetic variations may exist that affect the immune system’s production of anti-CCP antibodies and regulate inflammatory responses. This genetic polymorphism may lead to significant differences in ESR and anti-CCP antibody levels. Environmental factors are also key contributors—air pollution and the high incidence of infectious diseases in certain regions could impact immune function and inflammatory responses, potentially leading to elevated ESR and changes in anti-CCP antibody levels. Therefore, well-designed studies are needed to further verify the differences between RA-ILD and RA patients in various regions. Additionally, CRP levels were notably higher in the elderly RA-ILD patient group (age ≥60) compared to RA patients, a difference not observed in younger groups, possibly due to physiological changes or reduced immunity in older RA-ILD patients. However, since CRP and ESR are acute-phase reactants, their levels may fluctuate significantly throughout the disease course. Therefore, caution should be exercised when using them as biomarkers for RA-ILD. It is essential to consider the patient’s disease status or combine these markers with other indicators to comprehensively assess pulmonary involvement in RA patients.

Biomarkers related to interstitial lung disease encompass various types related to alveolar epithelial cell damage, fibroproliferation, extracellular matrix remodeling, and immune dysfunction (113), such as SP-D (114), KL-6 (115), and MMP-7 (116). Studie (34, 54) have shown that these biomarkers are elevated in RA-ILD. Our meta-analysis found significant differences in KL-6, SP-D, and MMP-7 between RA-ILD and RA patients. However, meta-regression indicates that the proportion of males is the source of heterogeneity for KL-6, suggesting that there may be differences in KL-6 levels between different genders. However, further exploratory research is still needed to verify this.

New biomarkers are gradually being identified. Tumor markers, mainly produced by malignant cells for screening or monitoring cancer progression, have been found elevated in RA-ILD patients (44, 47), independent of actual cancer presence, drawing widespread attention to their potential role in RA-ILD. Our meta-analysis indicates significant differences in CEA and CA19-9 between RA-ILD and RA patients, whereas no significant differences were observed for CA-125 and CA-153. This provides a reference for selecting precise tumor markers to differentiate between RA and RA-ILD. Blood cell-based indices like Neutrophil-to-Lymphocyte Ratio (NLR) and Platelet-to-Lymphocyte Ratio (PLR) have been reported as biomarkers of systemic inflammatory response, playing a significant role in various cancers, autoimmune rheumatic diseases, and cardiovascular diseases (117–119). Some studies have shown that PLR and NLR are significantly elevated in RA-ILD patients (18, 42). Our meta-analysis reveals a significant difference in NLR between RA-ILD and RA patients, but not for PLR. However, due to the limited number of studies included and the instability of results in sensitivity analysis, the pooled effects of tumor markers and NLR/PLR should be interpreted with caution, and more high-quality research is needed for validation.

Our meta-analysis highlights the differences between individual biomarkers in RA-ILD and RA patients, yet no single marker seems sufficient for diagnosing RA-ILD alone. Studies by Jérôme Avouac et al. (34) suggest that combining KL-6 with SP-D could enhance diagnostic capability for RA-ILD, offering a new approach to selecting suitable biomarkers for RA-ILD. We identified 78 potential biomarkers for distinguishing between RA and RA-ILD and selected 11 with significant differences through meta-analysis, laying the foundation for using multiple biomarkers in combination.

IL-13 is also a fibrogenic cytokine, and IL-13Rα1 and IL-13Rα2 are its two receptors. As transmembrane receptors, IL-13Rα1 can combine with IL-4Rα to form a stable complex (120), which activates the JAK/STAT signaling pathway, inducing TGF-β production and ultimately leading to fibrosis. A study by Manal Shawky Hussein et al. (25) demonstrated that serum IL-13 levels, along with IL-13Rα1 and IL-13Rα2, are positively correlated with HRCT scores (P < 0.001), suggesting that these markers may be used to assess the severity of interstitial lung disease in RA patients, showing high clinical value. However, due to the limited number of related studies, we did not conduct a meta-analysis.

The outcomes of our meta-analysis indicate that MMP-7 is moderately correlated with diffusing capacity of the lungs for carbon monoxide (DLCO), while KL-6 is weakly correlated with forced vital capacity (FVC) and DLCO, and HE4 is weakly correlated with percentage of predicted forced vital capacity (FVC%). This suggests that MMP-7, KL-6, and HE4 can reflect changes in lung function to some extent. For patients with idiopathic pulmonary fibrosis (IPF), FVC and DLCO are the most sensitive parameters for evaluating clinical course. Additionally, there is a strong correlation between progressively declining FVC and DLCO values and the clinical severity of ILD (121). ILD-induced deterioration in lung function affects prognosis and increases mortality rates. Early detection, timely treatment, and appropriate intervention are crucial for improving clinical symptoms and survival in RA-ILD patients (84), but lung function tests are costly, have poor repeatability, and low compatibility for critically ill patients. Selecting appropriate biomarkers to assess changes in lung function in patients has significant clinical value. However, the number of studies included in this meta-analysis is limited, and further exploratory research is needed to validate the results and select suitable biomarkers to assess the severity of RA-ILD.

A study by Natalia Mena-Vázquez et al. (25) found that during follow-up, 13 out of 35 RA-ILD patients (37.1%) experienced lung disease progression. Cox regression analysis revealed that the only variable associated with RA-ILD progression was IL-18 (pg/mL) (p= 0.227; p = 0.004). IL-18 is a member of the IL-1 cytokine superfamily, primarily produced by macrophages. Animal studies have shown that IL-18 can induce lung inflammation (122); in humans, elevated IL-18 levels have been observed in patients with idiopathic pulmonary fibrosis (123), ILD-related inflammatory myopathies (124), and RA-ILD (39). Although further research is needed to determine the exact role of this cytokine in RA-ILD, it may be associated with poorer prognosis in RA-ILD patients and provides a potential direction for future studies.

The findings of our meta-analysis indicate that KL-6 can serve as a prognostic predictor for RA-ILD patients. However, due to the limited number of included studies, the results should be interpreted with caution. Compared to interstitial lung disease associated with other connective tissue diseases, RA-ILD patients have a poorer prognosis (125). Prognostic evaluation at the time of diagnosis is clinically significant. However, there are currently few biomarkers available for RA-ILD prognostic assessment, and there is a lack of accurate, convenient, and cost-effective biomarkers. Further exploratory research is needed to identify and validate suitable biomarkers for assessing RA-ILD prognosis.

Our study has limitations. Firstly, some biomarker meta-analyses results exhibited high heterogeneity, and we could not identify sources of heterogeneity using meta-regression or subgroup analysis, possibly due to differences in environment, genetics, ethnicity, lifestyle, and testing methods. Secondly, although we observed differences in biomarker levels between RA-ILD and RA patients, the studies we included were observational studies and cannot determine cause and effect. Additionally, many studies included are retrospective, single-center, and small-scale, and some biomarkers had few studies, potentially affecting the stability of our results. Thus, more large-scale, multicenter, prospective cohort studies are needed to draw definitive conclusions or reinforce the findings of this study.

### 2.4 Conclusion

This review comprehensively summarizes biomarkers related to disease prediction, diagnosis, severity assessment, and prognosis evaluation in RA-ILD patients. These biomarkers show promising clinical applications and hold significant importance for early diagnosis, improving treatment efficacy, and enhancing the prognosis of RA-ILD patients. However, large-sample, multicenter, prospective cohort studies are still needed to validate these findings.

## Data Availability

The original contributions presented in the study are included in the article/**Supplementary Material**. Further inquiries can be directed to the corresponding author.
