# Supplementary material for: Biomarkers of rheumatoid arthritis-associated interstitial lung disease: a systematic review and meta-analysis
Source: Front Immunol. 2024 Oct 29;15:1455346. doi: 10.3389/fimmu.2024.1455346 (PMC11554464; doi:10.3389/fimmu.2024.1455346)
Supplement: Supplementary file 1 [file DataSheet1.pdf]

Supplementary Material

Supplementary Material

1. Search Strategy 2
2. Supplementary Table 1: Characteristics of the included studies 6
3. Meta-analysis results of the diagnosis of RA-ILD 20
   - CRP 20
   - ESR 25
   - Anti-CCP Antibody 30
   - RF 35
   - KL-6 40
   - SP-D 43
   - CEA 45
   - CA19-9 45
   - CA-125 46
   - CA-153 47
   - MMP-7 47
   - CXCL-10 48
   - PLR 49
   - NLR 49
4. Meta-analysis results of the Severity assessment of RA-ILD 50
   - KL-6 50
   - MMP-7 51
   - HE4 51
5. Meta-analysis results of the Prognostic assessment of RA-ILD 52
   - KL-6 52

1. **Search Strategy**

Search strategies for the different databases ran on **October 7, 2023.** This strategy is adapted to identify trials in other electronic databases

PubMed (1383)

Search number	Query	Search Details	Results
33	((((("Arthritis, Rheumatoid"[Mesh]) OR (RA[Title/Abstract])) OR ((felty*[Title/Abstract]) AND (syndrome[Title/Abstract]))) OR ((((((rheumatoid[Title/Abstract]) OR (reumatoid[Title/Abstract])) OR (rheumatic[Title/Abstract])) OR (rheumat*[Title/Abstract])) OR (reumat*[Title/Abstract])) AND (((((arthrit*[Title/Abstract]) OR (artrit*[Title/Abstract])) OR (diseas*[Title/Abstract])) OR (condition*[Title/Abstract])) OR (nodule*[Title/Abstract])))) AND (((((((((("Lung Diseases, Interstitial"[Mesh]) OR ("Bronchiolitis Obliterans"[Mesh])) OR (ILD[Title/Abstract])) OR (sarcoidosis*[Title/Abstract])) OR (alveolitis*[Title/Abstract])) OR (granulomatosis[Title/Abstract])) OR ((bronchiolitis[Title/Abstract]) AND (obliterans[Title/Abstract]))) OR ((diffuse*[Title/Abstract]) AND (parenchymal*[Title/Abstract]))) OR ((interstitial*[Title/Abstract]) AND ((((lung*[Title/Abstract]) OR (pulmonary*[Title/Abstract])) OR (pneumon*[Title/Abstract])) OR (fibros*[Title/Abstract])))) OR (((pulmonary*[Title/Abstract]) OR (lung*[Title/Abstract])) AND ((fibros*[Title/Abstract]) OR (fibrot*[Title/Abstract]))))) AND (((((((("Biomarkers"[Mesh]) OR (proteomics[Title/Abstract])) OR (genetic markers[Title/Abstract])) OR (metabolomics[Title/Abstract])) OR (immune[Title/Abstract])) OR (biological marker[Title/Abstract])) OR (biosignature[Title/Abstract])) OR (biomarker*[Title/Abstract])) OR (marker*[Title/Abstract]))	("arthritis, rheumatoid"[MeSH Terms] OR "RA"[Title/Abstract] OR ("felty*"[Title/Abstract] AND "syndrome"[Title/Abstract]) OR (("rheumatoid"[Title/Abstract] OR "reumatoid"[Title/Abstract] OR "rheumatic"[Title/Abstract] OR "rheumat*"[Title/Abstract] OR "reumat*"[Title/Abstract]) AND ("arthrit*"[Title/Abstract] OR "artrit*"[Title/Abstract] OR "diseas*"[Title/Abstract] OR "condition*"[Title/Abstract] OR "nodule*"[Title/Abstract]))) AND ("lung diseases, interstitial"[MeSH Terms] OR "Bronchiolitis Obliterans"[MeSH Terms] OR "ILD"[Title/Abstract] OR "sarcoidosis*"[Title/Abstract] OR "alveolitis*"[Title/Abstract] OR "granulomatosis"[Title/Abstract] OR ("bronchiolitis"[Title/Abstract] AND "obliterans"[Title/Abstract]) OR ("diffuse*"[Title/Abstract] AND "parenchymal*"[Title/Abstract]) OR ("interstitial*"[Title/Abstract] AND ("lung*"[Title/Abstract] OR "pulmonary*"[Title/Abstract] OR "pneumon*"[Title/Abstract] OR "fibros*"[Title/Abstract])) OR (("pulmonary*"[Title/Abstract] OR "lung*"[Title/Abstract]) AND ("fibros*"[Title/Abstract] OR "fibrot*"[Title/Abstract]))) AND ("Biomarkers"[MeSH Terms] OR "proteomics"[Title/Abstract] OR "genetic markers"[Title/Abstract] OR "metabolomics"[Title/Abstract] OR "immune"[Title/Abstract] OR "biological marker"[Title/Abstract] OR "biosignature"[Title/Abstract] OR "biomarker*"[Title/Abstract]	1,383

		OR "marker*"[Title/Abstract])	
32	(((((((("Biomarkers"[Mesh]) OR (proteomics[Title/Abstract])) OR (genetic markers[Title/Abstract])) OR (metabolomics[Title/Abstract])) OR (immune[Title/Abstract])) OR (biological marker[Title/Abstract])) OR (biosignature[Title/Abstract])) OR (biomarker*[Title/Abstract])) OR (marker*[Title/Abstract])	"Biomarkers"[MeSH Terms] OR "proteomics"[Title/Abstract] OR "genetic markers"[Title/Abstract] OR "metabolomics"[Title/Abstract] OR "immune"[Title/Abstract] OR "biological marker"[Title/Abstract] OR "biosignature"[Title/Abstract] OR "biomarker*"[Title/Abstract] OR "marker*"[Title/Abstract]	2,560,316
31	marker*[Title/Abstract]	"marker*"[Title/Abstract]	935,690
30	biomarker*[Title/Abstract]	"biomarker*"[Title/Abstract]	425,576
29	biosignature[Title/Abstract]	"biosignature"[Title/Abstract]	542
28	biological marker[Title/Abstract]	"biological marker"[Title/Abstract]	3,399
27	immune[Title/Abstract]	"immune"[Title/Abstract]	853,944

26	metabolomics[Title/Abstract]	"metabolomics"[Title/Abstract]	41,873
25	genetic markers[Title/Abstract]	"genetic markers"[Title/Abstract]	17,453
24	proteomics[Title/Abstract]	"proteomics"[Title/Abstract]	68,306
23	"Biomarkers"[Mesh]	"Biomarkers"[MeSH Terms]	886,095
22	(((((((((("Lung Diseases, Interstitial"[Mesh]) OR ("Bronchiolitis Obliterans"[Mesh])) OR (ILD[Title/Abstract])) OR (sarcoidosis*[Title/Abstract])) OR (alveolitis*[Title/Abstract])) OR (granulomatosis[Title/Abstract])) OR ((bronchiolitis[Title/Abstract]) AND (obliterans[Title/Abstract]))) OR ((diffuse*[Title/Abstract]) AND (parenchymal*[Title/Abstract]))) OR ((interstitial*[Title/Abstract]) AND ((((lung*[Title/Abstract]) OR (pulmonary*[Title/Abstract])) OR (pneumon*[Title/Abstract])) OR (fibros*[Title/Abstract])))) OR (((pulmonary*[Title/Abstract]) OR (lung*[Title/Abstract])) AND ((fibros*[Title/Abstract]) OR (fibrot*[Title/Abstract])))	"lung diseases, interstitial"[MeSH Terms] OR "Bronchiolitis Obliterans"[MeSH Terms] OR "ILD"[Title/Abstract] OR "sarcoidosis*"[Title/Abstract] OR "alveolitis*"[Title/Abstract] OR "granulomatosis"[Title/Abstract] OR ("bronchiolitis"[Title/Abstract] AND "obliterans"[Title/Abstract]) OR ("diffuse*"[Title/Abstract] AND "parenchymal*"[Title/Abstract]) OR ("interstitial*"[Title/Abstract] AND ("lung*"[Title/Abstract] OR "pulmonary*"[Title/Abstract] OR "pneumon*"[Title/Abstract] OR "fibros*"[Title/Abstract])) OR (("pulmonary*"[Title/Abstract] OR "lung*"[Title/Abstract]) AND ("fibros*"[Title/Abstract] OR "fibrot*"[Title/Abstract]))	193,380
21	((pulmonary*[Title/Abstract]) OR (lung*[Title/Abstract])) AND ((fibros*[Title/Abstract]) OR (fibrot*[Title/Abstract]))	("pulmonary*"[Title/Abstract] OR "lung*"[Title/Abstract]) AND ("fibros*"[Title/Abstract] OR "fibrot*"[Title/Abstract])	66,262
20	(fibros*[Title/Abstract]) OR (fibrot*[Title/Abstract])	"fibros*"[Title/Abstract] OR "fibrot*"[Title/Abstract]	276,668
19	(pulmonary*[Title/Abstract]) OR (lung*[Title/Abstract])	"pulmonary*"[Title/Abstract] OR "lung*"[Title/Abstract]	1,253,962
18	(interstitial*[Title/Abstract]) AND ((((lung*[Title/Abstract]) OR (pulmonary*[Title/Abstract])) OR (pneumon*[Title/Abstract])) OR (fibros*[Title/Abstract]))	"interstitial*"[Title/Abstract] AND ("lung*"[Title/Abstract] OR "pulmonary*"[Title/Abstract] OR "pneumon*"[Title/Abstract] OR "fibros*"[Title/Abstract])	52,297
17	(((lung*[Title/Abstract]) OR (pulmonary*[Title/Abstract])) OR (pneumon*[Title/Abstract])) OR (fibros*[Title/Abstract])	"lung*"[Title/Abstract] OR "pulmonary*"[Title/Abstract] OR "pneumon*"[Title/Abstract] OR "fibros*"[Title/Abstract]	1,622,134
16	interstitial*[Title/Abstract]	"interstitial*"[Title/Abstract]	116,377
15	(diffuse*[Title/Abstract]) AND (parenchymal*[Title/Abstract])	"diffuse*"[Title/Abstract] AND "parenchymal*"[Title/Abstract]	2,461
14	(bronchiolitis[Title/Abstract]) AND (obliterans[Title/Abstract])	"bronchiolitis"[Title/Abstract] AND "obliterans"[Title/Abstract]	4,039
13	granulomatosis[Title/Abstract]	"granulomatosis"[Title/Abstract]	12,470
12	alveolitis*[Title/Abstract]	"alveolitis*"[Title/Abstract]	4,344
11	sarcoidosis*[Title/Abstract]	"sarcoidosis*"[Title/Abstract]	28,648
10	ILD[Title/Abstract]	"ILD"[Title/Abstract]	7,095
9	"Bronchiolitis Obliterans"[Mesh]	"Bronchiolitis Obliterans"[MeSH Terms]	4,302

8	"Lung Diseases, Interstitial"[Mesh]	"lung diseases, interstitial"[MeSH Terms]	85,455
7	((("Arthritis, Rheumatoid"[Mesh]) OR (RA[Title/Abstract])) OR ((felty*[Title/Abstract]) AND (syndrome[Title/Abstract]))) OR ((((((rheumatoid[Title/Abstract]) OR (reumatoid[Title/Abstract])) OR (rheumatic[Title/Abstract])) OR (rheumat*[Title/Abstract])) OR (reumat*[Title/Abstract])) AND (((((arthrit*[Title/Abstract]) OR (artrit*[Title/Abstract])) OR (diseas*[Title/Abstract])) OR (condition*[Title/Abstract])) OR (nodule*[Title/Abstract])))	"arthritis, rheumatoid"[MeSH Terms] OR "RA"[Title/Abstract] OR ("felty*"[Title/Abstract] AND "syndrome"[Title/Abstract]) OR (("rheumatoid"[Title/Abstract] OR "reumatoid"[Title/Abstract] OR "rheumatic"[Title/Abstract] OR "rheumat*"[Title/Abstract] OR "reumat*"[Title/Abstract]) AND ("arthrit*"[Title/Abstract] OR "artrit*"[Title/Abstract] OR "diseas*"[Title/Abstract] OR "condition*"[Title/Abstract] OR "nodule*"[Title/Abstract]))	254,481
6	(((((rheumatoid[Title/Abstract]) OR (reumatoid[Title/Abstract])) OR (rheumatic[Title/Abstract])) OR (rheumat*[Title/Abstract])) OR (reumat*[Title/Abstract])) AND (((((arthrit*[Title/Abstract]) OR (artrit*[Title/Abstract])) OR (diseas*[Title/Abstract])) OR (condition*[Title/Abstract])) OR (nodule*[Title/Abstract]))	("rheumatoid"[Title/Abstract] OR "reumatoid"[Title/Abstract] OR "rheumatic"[Title/Abstract] OR "rheumat*"[Title/Abstract] OR "reumat*"[Title/Abstract]) AND ("arthrit*"[Title/Abstract] OR "artrit*"[Title/Abstract] OR "diseas*"[Title/Abstract] OR "condition*"[Title/Abstract] OR "nodule*"[Title/Abstract])	181,938
5	((((arthrit*[Title/Abstract]) OR (artrit*[Title/Abstract])) OR (diseas*[Title/Abstract])) OR (condition*[Title/Abstract])) OR (nodule*[Title/Abstract])	"arthrit*"[Title/Abstract] OR "artrit*"[Title/Abstract] OR "diseas*"[Title/Abstract] OR "condition*"[Title/Abstract] OR "nodule*"[Title/Abstract]	7,222,386
4	((((rheumatoid[Title/Abstract]) OR (reumatoid[Title/Abstract])) OR (rheumatic[Title/Abstract])) OR (rheumat*[Title/Abstract])) OR (reumat*[Title/Abstract])	"rheumatoid"[Title/Abstract] OR "reumatoid"[Title/Abstract] OR "rheumatic"[Title/Abstract] OR "rheumat*"[Title/Abstract] OR "reumat*"[Title/Abstract]	216,876
3	(felty*[Title/Abstract]) AND (syndrome[Title/Abstract])	"felty*"[Title/Abstract] AND "syndrome"[Title/Abstract]	782
2	RA[Title/Abstract]	"RA"[Title/Abstract]	92,942
1	"Arthritis, Rheumatoid"[Mesh]	"arthritis, rheumatoid"[MeSH Terms]	126,408

2. **Supplementary Table 1: Characteristics of the included studies**

First author	Year	Region	RA Classification criteria	Study design	Biomarker	Detection method	RA-ILD			RA without ILD			Quality score
							sample size	Average age (years)[a]	M/F	sample size	Average age (years)[a]	M/F	
Kronzer, V	2023	USA	1987 ACR or ACR/EULAR 2010 criteria	Case-control	FS-ACPA	Bio-Rad Laboratories, Hercules, CA, USA;Luminex 200 Luminex, Austin, TX, USA	84	67±10	25/59	233	66±11	47/186	8
Mena-Vázquez, N	2023	Spain	ACR/EULAR 2010 criteria	Cohort study	anti-CCP，MCP-1, CCL2, SDF-1 α, IL-18	NA	35	69.7±9.3	20/15	35	66.6±7.0	20/15	8
Nava-Quiroz, K	2023	Mexico	NA	Cross-sectional	PADI2(rs2057094,rs2076615), PADI4(rs1748033)	Maxim Biotech, CA, USA	118	61.50 (53.00,67.00)	38/80	133	54 (44,62)	10/123	5
Yu, R	2023	China	ACR/EULAR 2010 criteria	Case-control study	CHI3L1	YHLO Biotech Co,Shenzhen, China	60	68.00 (62.00,71.75)	21/39	179	48.00 (37.00, 57.00)	149/30	7
Zheng, W	2023	China	ACR/EULAR 2010 criteria	Case-control study	IL-36α，IL-36γ	R&D Systems, Minneapolis, MN, USA	39	65.05±13.24	22/17	35	61.63±11.71	23/12	7
Brink, M	2022	Sweden	1987 ACR	Cohort study	FS-ACPA	EuroDiagnostica, Malmo , Sweden	50	63.90±10.35	29/21	791	56.98±13.85	548/243	9
Chen, J	2022	China	1987 ACR	Cohort study	CXCL11，MMP-13	eBioscience, Procarta	38	47.6±16.6	8/30	22	38.9 ± 16	4/18	8
Chen, N	2022	China	1987 ACR or ACR/EULAR 2010	Cohort study	RF	Enzyme-linked immunosorbent assay (AESKU)	75	65.7±8.4	14/29	NA	NA	NA	8

			criteria										
Hayashi, S	2022	Japan	1987 ACR	Cohort study	rs6578890 in PPFIBP2	NA	57	66.5	13/44	249	58.6	27/222	7
Lin, T	2022	China	ACR/EULAR 2010 criteria	Case-control	HE4	Electrochemiluminescence Immunoassay Roche Diagnostics GmbH, Mannheim	46 RA-ILD	63.35 ± 8.16	15/31	56	58.29 ± 8.83	27/29	9
Matson, S	2022	United Kingdom	ACR 2010 criteria	Cohort study	MUC5B mutations	Thermo Fisher Scientific	NA	NA	NA	184	56.4 (44.2-66.6)	18/166	8
Natalini, J	2022	USA	1987 ACR	Cross-sectional study	qPCR (T/S ratio)	Qubit Fluorometric Assay (Invitrogen)	54	68.5 (62, 75)	51/3	92	66 (61, 72)	87/15	11
Pulito-Cueto, V	2022	Spain	ACR 2010 criteria	Case-control	VCAM-1, MCP-1, ADMA	VCAM-1: BMS232, Invitrogen, Vienna, Austria; MCP-1: BMS281, Invitrogen, Vienna, Austria; ADMA: K7860, Immundiagnostik AG, Bensheim, Germany	21	66.5 ± 10.1	12/9	25	60.1 ± 11.8	10/15	7
Qin, Y	2022	China	1987 ACR	Case-control	KL-6，D-dimer，CA19-9	Kaeser 6600 Chemiluminescence Immunoassay	75	62.84 ± 8.71	30/45	78	45.15 ± 13.31	20/58	8
Venerito, V	2022	Italy	ACR/EULAR 2010 criteria	Cohort study	HRCT	NA	30	72(65,78)	11/19				8
Wang, X	2022	China	ACR/EULAR 2010 criteria	Case-control	IL-11	Enzyme-Linked Immunosorbent Assay:Enzyme-Linked Immunosorbent Assay	31	59.84 ± 7.10	6/13	75	58.83 ± 8.76	5/35	7
Wang, Z	20	China	ACR/EULA	Case-	UA,KL-6	Enzyme-Linked	162	54.66 ±		104	63.85 ±	20/8	9

	2022		R 2010 criteria	control		Immunosorbent Assay		3.94	28/134		13.20	4	
Xu, Y	2022	China	ACR/EULAR 2010 criteria	Case-control	SIRI	Sysmex, Tokyo, Japan	284	68.95 ± 9.44	104/180	1215	57.95 ± 11.97	274/941	9
Fotoh, D	2021	Egypt	ACR 2010 criteria	Case-control	KL-6	Latex-enhanced immunoturbidimetric assay(SEKISUI Medical Co., Ltd. (Japan))	75	47.4±10.5	40/35	75	45.3±11.5	39/36	7
Hammoda, R	2021	Egypt	ACR/EULAR 2010 criteria	Case-control	KL-6	Enzyme-linked immunosorbent assay (MyBioSource, San Diego, United States)	64	43.9±9.6	7/18	25	47.7±11.8	15/49	7
Hussein, M	2021	Egypt	ACR/EULAR 2010 criteria	Case-control	IL-13,IL-13 Rα1,IL-13 Rα2	Enzyme-linked immunosorbent assay (ELISA)	50	67.27 ± 10.87	15/35	50	55.76 ± 8.43	3/47	8
Liang, L	2021	China	ACR/EULAR 2010 criteria	Cohort study	HE4	Roche Cobas Electrochemiluminescence Analyzer (Hoffmann-La Roche AG,Basel, Switzerland)	48	67(57,73)	12/36	120	62(53,70)	9/111	7
Mena-Vázquez, N	2021	Spain	ACR/EULAR 2010 criteria	Case-control	DLCO	NA	41	67.9 ± 7.8	21/20	41	67.6 ± 8.8	22/19	9
Moon, J	2021	South Korea	ACR/EULAR 2010 criteria	Cohort study	MMP-7,SP-D，KL-6	Nanopia KL-6（Sekisui Medical Co.，LTD，Tokyo, Japan);MSD R-PLEX（Meso Scale Discovery）	153	66.35±8.22	46/107	NA	NA	NA	7
Nakazawa, M	2021	Japan	NA	Case-control	coinhibitory molecules on alveolar T cells	NA	8	NA	NA	8	NA	NA	4

Saku, A	2021	Japan	1987 ACR or ACR/EULAR 2010 criteria	Case-control	monocyte and neutrophil counts	NA	72	68.6	42/30	NA	NA	NA	8
Tanaka, N	2021	Japan	ACR/EULAR 2010 criteria	Cohort study	KL-6 change rate over years	SEKISUI MEDICAL CO.,LTD.	125	NA	22/11	NA	NA	NA	9
Tyker, A	2021	USA	ACR/EULAR 2010 criteria	Case-control	RF	NA	70	63.3 ±10.2	18/24	NA	NA	NA	9
Xu, L	2021	China	ACR/EULAR 2010 criteria	Case-control	sPD 1	Enzyme-linked immunosorbent assay (ELISA)(Invitrogen)	58	65.7 ± 9.4	18/36	29	61.8 ± 9.2	4/25	9
Xue, J	2021	China	ACR/EULAR 2010 criteria	Cohort study	cDKK-1	Enzyme-linked immunosorbent assay (ELISA)	35	60.4 ± 1.6	17/18	67	NA	NA	7
Zheng, M	2021	China	1987 ACR	Case-control	KL-6,CA19-9,CA125,CEA	Bayer Centaur Automated Chemiluminescence Immunoassay System;Chemiluminescence Immunoassay (CLIA)(LumipulseG1200)	24	62.9 ± 11.8	14/10	26	56.6 ± 10.3	6/20	8
Allam, A	2020	Saudi Arabia	ACR/EULAR 2010 criteria	Case-control	ACPA	Enzyme-Linked Immunosorbent Assay (ELISA)(Euroimmun、Lubeck)（Behring）	15	58.07±15.06	4/11	30	55.93±10	9/21	7
Avouac, J	2020	France, Japan,	1987 ACR or	Cohort study	SP-D,CCL-18,KL-6	Enzyme-Linked Immunosorbent Assay	40	71±15	18/22	107	62±12	27/80	8

		Switzerland	ACR/EULAR 2010 criteria			(ELISA)（Sekisui Medical Co., Tokyo, Japan）							
Del Angel-Pablo, A	2020	Mexico	ACR/EULAR 2010 criteria	Case-control	HLA-DRB1, HLA-DRB1 SE, HLA-DQB1	LABScreen PRA（One Lambda，West Hills，CA，USA）	65	61(37–85)	12/53	82	53.50(25–80)	1/81	6
Furukawa, H	2020	Japan	1987 ACR or ACR/EULAR 2010 criteria	Case-control	Decanoic acid, morpholine, glycerol	MetaboAnalyst 4.0	100	67.3 ±8.5	24/76	100	66.2 ±8.9	24/76	9
Kass, D	2020	USA	1987 ACR	Cohort study	MMP-1,MMP-2,MMP-7,MMP-9,IL-1RA,sCD40L，CXCL9	Luminex xMAP、bead-based multiplex Enzyme-Linked Immunosorbent Assay (ELISA) (EMD Millipore)	22	49 (44-55)	5/17	49	65 (58-75)	18/31	7
Kim, H	2020	South Korea	ACR/EULAR 2010 criteria	Cohort study	KL-6	Nanoopia KL-6（SEKISUI MEDICAL，Tokyo）	84	65.8± 7.9	16/14	NA	NA	NA	8
Pulito-Cueto, V	2020	Spain	ACR 2010 criteria	Case-control	EPC	Direct flow cytometry	20	66.8±10.2	11/9	25	60.1±11.8	10/15	6
Wu, X	2020	China	1987 ACR	Cross-sectional	PD-L1	Enzyme-Linked Immunosorbent Assay (ELISA)(Invitrogen,United States)	58	65.7±9.4	22/36	29	61.8±9.2	4/25	8
Zhou, W	2020	China	ACR/EULAR 2010 criteria	Case-control	lncRNA(NR_002819,NR_038935,ENST00000603415,ENST00000560199)	TRIzol (Invitrogen, Carlsbad, CA, USA) 从PBMC;N ano D rop 1 0 0 0	20	57.65±6.99	0/20	20	52.6±8.62	0/20	6
Chen, Q	20	China	1987 ACR	Case-	PLR	Automated blood cell	103	60.94±1	24/	198	59.80±	39/1	7

	19		or ACR/EULAR 2010 criteria	control		counting system (Sysmex, Tokyo, Japan)		0.35	79		11.21	59	
Matsuo, T	2019	Japan	1987 ACR or ACR/EULAR 2010 criteria	Cross-sectional	IL-18,anti-CCP,KL-6	Enzyme-linked immunosorbent assay (ELISA) (MBL, Nagoya, Japan)	26	69.8±9.5	5/21	286	62.9±12.7	36/250	9
Yu, M	2019	China	1987 ACR	Cohort study	Wnt5a	Enzyme-Linked Immunosorbent Assay (ELISA)	40	60.14 ± 1.67	12/17	NA	NA	NA	7
Alunno, A	2018	Italy	1987 ACR	Cohort study	ACPA	Enzyme-Linked Immunosorbent Assay (ELISA)（EliA - Thermo Scientific）	79	NA	NA	21	NA	NA	8
Fu, Q	2018	China	ACR/EULAR 2010 criteria	Cross-sectional	LOXL2	根 Enzyme-Linked Immunosorbent Assay (ELISA)（Catalog number CSB-EL013041HU, CUSABIO, China）	49	65.86 ± 11.92	19/30	43	61.51 ± 11.24	13/30	10
Wu, X	2018	USA	NA	Cohort study	Proteomic Profiling	SOMAscan assay	39	65±9	11/28	36	57± 12	3/33	6
Oka, S	2017	Japan	1987 ACR	Case-control	hsa-miR-214-5p, hsa-miR-7-5p	miR-CURY RNA Isolation Kit Biofluids（Exiqon，Vedbaek，Denmark）; miRCURY LNA Universal cDNA Synthesis Kit II (Exiqon)	32	70.2 (7.4)	11/21	32	57.7 (13.8)	2/30	6

Lee, Y	2016	South Korea	ACR/EULAR 2010 criteria	Cohort study	KL-6,IL-6	Enzyme-Linked Immunosorbent Assay (ELISA):KL-6 (Eidia Co. Ltd., Tokyo, Japan), SP-A (BioVendor Laboratory Medicine, Inc., Brno, Czech Republic), MMP-7 (R&D Systems, Minneapolis, Minnesota, USA), IL-6 (R&D Systems, Minneapolis, Minnesota, USA), and IL-32 (Cusabio, Inc., China)	51	66±9	9/6	NA	NA	NA	7
Sileem, A	2016	Egypt	ACR/EULAR 2010 criteria	Case-control	SP-D	Enzyme-Linked Immunosorbent Assay (ELISA)(Massachusetts, Endogen)	18	49±11	8/10	12	47±12	4/8	6
Suhara, K	2016	Japan	1987 ACR	Case-control	Fragmented gelsolins	FluoroPhoreStar 3000（Anatech，Tokyo, Japan）	12	62 (54,68)	4/1	NA	NA	NA	6
Wang, T	2016	China	1987 ACR	Case-control	CEA,CA15-3,CA19-9,CA125	Electrochemiluminescence Immunoassay	28	63.36 ± 11.26	16/12	83	54.10 ± 15.52	30/53	7
Chen, J	2015	China	1987 ACR	Cohort study	MMP-7, CXCL10	Enzyme-Linked Immunosorbent Assay (ELISA)（eBioscience、Procarta）	41	53.02 ± 14.20	12/29	50	43.4 ± 15.54	9/41	8
		USA（Validation	1987 ACR	Cohort study	MMP-7, CXCL10	Enzyme-Linked Immunosorbent Assay (ELISA)	41	65.27 ± 10.80	11/30	22	50.32 ± 7.82	6/16	

		Cohort Study ）				（eBioscience、Procarta）							
Doyle, T	2015	USA	1987 ACR	Cohort study	MMP-7,PARC,SP-D	Multiplex bead-based immunoassay	46	65 ± 10	23/23	29	53 ± 12	1/29	6
		USA （Validation Cohort Study ）	1987 ACR	Cohort study	MMP-7,PARC,SP-D	Multiplex bead-based immunoassay	39	64 ± 14	26/13	22	50 ± 8	6/16	
Inui, Naoki;	2008	Japan	1987 ACR	Case-control	anti-CCP	Enzyme-Linked Immunosorbent Assay (ELISA)（ Diastat ；Axis-Shield Diagnostics Limited，Dundee, United Kingdom）	18	NA	10/8	36	NA	11/25	7
Nakashita, T	2014	Japan	NA	Cohort study	KL-6	NA	58	66.1±9.1	26/32	108	57.8±12.6	19/86	9
Harlow, L	2013	USA	1987 ACR	Cross-sectional	Hsp90	Solid-phase ELISA (Enzyme-Linked Immunosorbent Assay)	58	NA	NA	27	NA	NA	10
Ji, Y	2008	China	1987 ACR	Case-control	TGF-β1, MMP-9, TIMP-1	Enzyme-linked immunosorbent assay (ELISA)(Shanghai Lingtian Biotechnology Co., Ltd.)	28	54．0±7．8	5/23	29	54．0±7．8	7/22	7
Atkins, S	2006	USA	1987 ACR	Case-control study	CD20+B cellsand CD138+plasm	NA	18	NA	10/8	11	NA	6/5	6

					a cells							
Turesson, C	2005	USA	1987 ACR	Case-control study	T cell subsets	NA	15	64 (57,72)	6/9	16	64 (57,69)	8/8	7
Oyama, T	1999	Japan	1987 ACR	Cohort study	KL-6	Sandwich Enzyme-Linked Immunosorbent Assay (ELISA)	32	62.94-12.4	4/3	NA	NA	NA	8
Oyama, T	1997	Japan	NA	Cohort study	KL-6	Sandwich Enzyme-Linked Immunosorbent Assay (ELISA)	177	60.6±11.6	35/142	9	NA	NA	8
S. Cao	2023	China	ACR/EULAR 2010 criteria	Cohort study	sCD25	BIO-RAD and Enzyme-Linked Immunosorbent Assay (ELISA) method	15	59.99 ± 15.47	16/56	57	NA	NA	
	2023	China（Validation Cohort Study）	ACR/EULAR 2010 criteria	Cohort study	sCD25	BIO-RAD and Enzyme-Linked Immunosorbent Assay (ELISA) method	181	60.73 ± 10.98	25/197	41	NA	NA	7
Goushi Matama	2023	Japan	ACR/EULAR 2010 criteria	Case-control trial	POSTN	Enzyme-Linked Immunosorbent Assay (ELISA)	19	70(65,76)	6/13	20	66(60,73)	4/16	7
Jill A. Poole	2023	USA	1987 ACR	Case-control	CD16+MO	NA	15	73(57,78)	13/2	15	73(68,79)	14/1	6
Anna Wierczeiko	2023	Germany	1987 ACR	Case-control	ARG1, TYMS, MKI67, OLFM4, BIRC5, MS4A4A, CLEC12A, LINC02967	PAXgene Blood miRNA Kit (PreAnalytiX)	7	NA	NA	5	NA	NA	6
Yasmine S. Makarema	2023	Spain	ACR/EULAR 2010 criteria	Cross-sectional	CXCL10	Double Antibody Sandwich Enzyme-Linked Immunosorbent	33	52.52 ± 10.21	1/32	40	43.70 ± 10.35	3/37	6

						Assay							
P.-A. Juge	2018	France	1987 ACR or ACR/EULAR 2010 criteria	Case-control	MUC5B mutations	TaqMan Genotyping Assay (Applied Biosystems)	620	69.0±10.8	565/345	614	60.4±12.6	540/446	9
Liangyu Chen	2024	China	NA	Case-control	THBS2, TIMP1, POSTN, CD19	NA	6	63.67 ± 3.71	3/3	6	56.50 ± 6.72	4/2	9
Hiroshi Furukawa	2012	Japan	1987 ACR	Case-control	HLA-DRB1, HLA-DRB1 SE, HLA-DQB1	WAKFlow HLA Typing Kit (Wakunaga, Hiroshima, Japan)	129	69.5±7.8)	42/87	321	61.7 ±11.2)	47/274	7
Shomi Oka	2016	Japan	1987 ACR	Case-control	HLA-DRB1, HLA-DQB1	PCR-SSOP technology (WAKFlow HLA Typing Kit, Wakunaga, Hiroshima, Japan), and Bio-Plex 200 System (Bio-Rad, Hercules, CA, USA).	290	69.8±9.2	96/194	773	61.3±12.2	117/656	8
Zhu Xiangyang	2016	China	1987 ACR	Case-control	IL-33,MMP-3	Enzyme-Linked Immunosorbent Assay (ELISA) (R&D Systems, Minneapolis, Minnesota, USA).	121	51 ± 17	19/102	47	47 ± 14	8/39	6
Alessia Alunno	2018	Italy	ACR/EULAR 2010 criteria	Cohort study	aCEP-1	Enzyme-Linked Immunosorbent Assay (ELISA)（Euroimmun，Luebeck，Germany）	183	NA	NA	37	NA	NA	7
Raul	20	Spain	ACR/EULA	Cross-	Anti-CarP	Enzyme-Linked	37	67.3±10.	12/	142	57.7±1	22/1	10

Castellanos-Moreira	20		R 2010 criteria	sectional		Immunosorbent Assay (ELISA)		1	25	RA-noILD	2.9	16	
Chase S. Correia	2019	USA	ACR/EULAR 2010 criteria	Cohort study	anti-CCP	Enzyme-Linked Immunosorbent Assay (ELISA)	2030	79.2 (77.4–81.0)	NA	NA	NA	NA	8
Bryant R. England, MD	2019	USA	1987 ACR	Case-control	Anti-MAA	Enzyme-Linked Immunosorbent Assay (ELISA)	90	67.0±9.9	86/4	1439	62.8±11.3	1283/156	7
Samia Faddaa	2018	Egypt	ACR/EULAR 2010 criteria	Core sectional study	ACPA	Enzyme-Linked Immunosorbent Assay (ELISA)	63	50±9.2	8/55	25	48.4 ± 8.2	5/20	9
Jon T. Giles	2014	USA	1987 ACR	Cohort study	Anti-PAD3/4 antibodies	NA	58	58±8	43/15	118	61±9	29/89	8
Jon T Giles	2014	USA	1987 ACR	Cross-sectional	FS-ACPA	NA	57	61±9	28/29	120	58±8	43/77	8
Clive A. Kelly	2014	United Kingdom	1987 ACR	Case-control study	anti-CCP	NA	230	NA	75/155	230	NA	60/170	7
Na-Lin Lai	2019	China	1987 ACR	Case-control	T cell subsets, NK cell subsets	Westergren Erythrocyte Sedimentation Rate (ESR) Method,immunoturbidimetry	100	63.89±8.99	43/57	100	53.26±13.24	25/75	7
Maniwa, K	2000	Japan	1987 ACR	Case-control	IL-1α autoantibodies	Radioimmunoassay (RIA)	32	63± 8	15/17	38	56± 16	10/28	7
Matsushita, Masakazu	2018	Japan	ACR/EULAR 2010 criteria	Case-control	anti-ARS antibodies	Myositis Profile Euroline Blot Euroimmun AG，Lübeck,Germany）	228	62.9±14.0	44/184	56	NA	NA	7
Jake G. Natalini	2021	USA	1987 ACR	Cohort study	anti-CCP, RF	Enzyme-Linked Immunosorbent Assay (ELISA)	2328	64 (58,71)	2065/263	NA	NA	NA	7

Restrepo, José Félix	2015	USA	1987 ACR	Case-control study	anti-CCP, RF	Solid-phase Enzyme Immunoassay (EIA) (TheraTest, Inc., Chicago, Illinois, USA)	69	60.2±9.7	34/35	563	52.9±13.5	127/439	7
Sargın, Gökhan	2022	Turkey	ACR/EULAR 2010 criteria	Cross-sectional	iPF	Mindray BC-6800 Hematology Analyzer, Hamburg, Germany	33	62.1±8.9	12/21	50	57.6 ± 10.7	10/40	9
Ji Ae Yang	2019	South Korea	1987 ACR	Cohort study	RF,anti-CCP,ESR,CRP	immunoturbidimetric assay,Chemiluminescent Microparticle Immunoassay	77	56.6±13.1	19/58	231	57.1±11.7	57/174	8
Yufeng Yin	2014	China	1987 ACR	Case-control	anti-CCP	Enzyme-Linked Immunosorbent Assay (ELISA) (Euroimmun, Lübeck)，Turbiidimetric Assay (Berlin, Germany)	71	58.3±11.2	21/50	214	49.5±13.4	53/161	7
Seung Taek Song	2016	South Korea	1987 ACR	Cross-sectional	RF	Immunoassay by Turbidimetry,Immunoassay by Turbidimetry	8	62.8 ± 8.8	3/5	22	49.2 ± 10.0	0/22	11
Huawei Dong	2019	USA	1987 ACR	Cohort study	RF,anti-CCP	Turbidimetric Method	7	52(47-63)	3/4	11	49(38-56)	4/11	7
Fausto Salaffi, MD, PhD	2019	Italy	1987 ACR	Case-control	RF,anti-CCP	NA	29	66.58 ±10.26	NA	122	54.66 ±7.80	NA	8
Hong Zhu	2019	China	ACR/EULAR 2010 criteria	Case-control	Anti-CarP	NA	129	60±10	29	57	48±13	7	8
Na Wang	2020	China	ACR/EULAR 2010 criteria	Cohort study	MUC5B mutations	NA	45	59.5±8.7	18/27	51	46.1±12.2	14/37	8
Jun-Xiang	20	China	ACR/EULA	Case-	anti-CCP	NA	83	59.60±9.	29/	461	50.54±	88/3	8

Wang	15		R 2010 criteria	control				66	54		13.76	73	
Juan Chen	2013	China	ACR/EULAR 2010 criteria	Case-control	anti-CCP	Enzyme-Linked Immunosorbent Assay (ELISA)	63	53.0±14.8	17/46	40	42.9 ±12.4	10/30	8
Shunsuke Mori	2012	Japan	1987 ACR	Cross-sectional	anti-CCP,RF,HLA-DRB1 SE	Enzyme-Linked Immunosorbent Assay (ELISA)	24	72.5 (64.0,76.3)	12/12	302	59.0 (52.0,68.0)	70/232	11
Gökhan SARGIN	2018	Turkey	ACR/EULAR 2010 criteria	Case-control	CEA,CA-153,CA19-9,CA-125	NA	43	60.1±11.5	13/30	40	58.5±12.7	7/33	8
Ting Wang	2015	China	ACR/EULAR 2010 criteria	Cross-sectional study	CEA,CA-153,CA19-9,CA-125	NA	25	63.56±11.90	14/11	16	56.19±12.11	6/10	9
Jian Zhang	2018	China	ACR/EULAR 2010 criteria	Case-control	CEA,CA-153,CA19-9	Immunoassay by turbidimetry,Enzyme-Linked Immunosorbent Assay (ELISA)	28	NA	11/17	47	NA	19/28	8

Abbreviation: ACR, american college of rheumatology; EULAR, european alliance of associations for rheumatology; RA, rheumatoid arthritis; ILD, interstitial lung disease; RA-ILD, rheumatoid arthritis-interstitial lung disease; Anti-PAD3/4 antibodies, Anti-PAD3/4 antibodies ; anti-CCP, anti-cyclic citrullinated peptide antibody; RF, rheumatoid factor; KL-6, krebs von den lungen-6; MMP-7, matrix metalloproteinase-7; SP-D, surfactant protein D; HE4, human epididymis protein 4; CRP, C-reactive protein; ESR, erythrocyte sedimentation rate; CEA, carcinoembryonic antigen; CA19-9, carbohydrate antigen 19-9; CA-125, cancer antigen 125; CA-153, cancer antigen 153; CXCL10, C-X-C motif chemokine ligand 10; MCP-1, monocyte chemoattractant protein-1; CCL2, chemokine (C-C motif) ligand 2; SDF-1 α, stromal cell-derived factor 1 alpha; IL-18, interleukin-18; CHI3L1, chitinase-3-like protein; IL-36α, interleukin-36 alpha; IL-36γ, Interleukin-36 alpha; qPCR (T/S ratio), quantitative polymerase chain reaction (T/S ratio); VCAM-1, vascular cell adhesion molecule-1; MCP-1, monocyte chemoattractant protein-1; ADMA, asymmetric dimethylarginine; UA, uric acid; SIRI, Simplified Index for the Robustness of Inflammation; sPD-1, soluble programmed cell death protein 1; cDKK-1, Circulating Dickkopf-1; CA, caprylic acid; GL, glycerol; PIP, piperidine; MMP-1, matrix metalloproteinase-1; MMP-2, matrix metalloproteinase-2; MMP-9, matrix metalloproteinase-9; IL-1RA, interleukin-1 receptor antagonists; sCD40L, soluble CD40 ligand; CXCL9, C-X-C motif chemokine ligand 9, EPC, endothelial progenitor cells; PD-L1, programmed cell death ligand 1; PLR, platelet-to-lymphocyte ratio; Wnt5a, non-canonical Wnt signaling representative ligand Wnt5a; aCEP-1, anti-C-terminal epitope antibody 1; LOXL2, lysyl oxidase-like 2; CCL18, C-C motif chemokine ligand 18; IL-17, interleukin-17; CXCL12, C-X-C motif chemokine ligand 12;

CCL5, C-C motif chemokine ligand 5; FGF4, fibroblast growth factor 4; FGF7, fibroblast growth factor 7; PARC, pulmonary and activation-regulated chemokine; Hsp90, heat shock protein 90; TGF-β1, transforming growth factor beta 1; sCD25, soluble CD25; POSTN, periostin; CD16+MO, CD16-positive monocytes; ARG1, arginase 1; TYMS, thymidylate synthase; SORT1, sortilin 1; MKI67, marker of proliferation Ki-67; OLFM4, Olfactomedin 4; BIRC5, baculoviral IAP repeat containing 5; MS4A4A, membrane-spanning 4-domains subfamily A member 4A; CLEC12A, C-type lectin domain family 12 member A; LINC02967, long intergenic non-protein coding RNA 2967; HLA-DRB1 SE, human leukocyte antigen class II DRB1 shared epitope; IL-33, interleukin-33; Anti-CarP, anti-carbamylated protein antibodies; Anti-MAA, malondialdehyde-acetaldehyde; IL-1α autoantibodies, interleukin-1 alpha autoantibodies; anti-ARS antibodies, anti-aminoacyl-tRNA synthetase antibodie; iPF, immature platelet fraction; IL-13 Rα1, interleukin-13 receptor alpha 1; TIMP-1, tissue inhibitor of metalloproteinases; FS-ACPA, fine-specificity anti-citrullinated protein antibodies; THBS2, thrombospondin-2; TIMP1, tissue inhibitor of metalloproteinases 1; CD19, cluster of differentiation 19; DLCO, diffusing capacity of the lungs for carbon monoxide; HRCT, high-resolution computed tomography

a: Values are presented as median (range), Median (IQR) or mean ± SD.

3. **Meta-analysis results of the diagnosis of RA-ILD**

1 CRP

Study	Treatment N	Treatment Mean	Treatment SD	Control N	Control Mean	Control SD	MD with 95% CI	Weight (%)
**Africa**								
Dina S. Fotoh 2021	75	53.2	21.03	75	44.7	18.9	8.50 [2.10, 14.90]	5.76
Manal Shawky Hussein 2021	50	46	43	50	35	33	11.00 [-4.02, 26.02]	4.53
Ashraf E. Sileem 2016	18	42.2	14.5	12	36.4	12.8	5.80 [-4.32, 15.92]	5.28
Heterogeneity: τ^2 = 0.00, I^2 = 0.00%, H^2 = 1.00							8.10 [3.02, 13.19]	
Test of $\theta_i = \theta_j$: Q(2) = 0.36, p = 0.84								
Test of θ = 0: z = 3.12, p = 0.00								
**America**								
Vanessa L Kronzer 2023	84	50.09	64.87	233	26.96	32.08	23.13 [12.35, 33.91]	5.19
Karol J. Nava-Quiroz 2023	118	19.42	23.19	133	17.13	21.74	2.29 [-3.27, 7.85]	5.84
Heterogeneity: τ^2 = 198.01, I^2 = 91.19%, H^2 = 11.34							12.18 [-8.22, 32.57]	
Test of $\theta_i = \theta_j$: Q(1) = 11.34, p = 0.00								
Test of θ = 0: z = 1.17, p = 0.24								
**Asia**								
Weishuai Zheng 2023	39	34.25	46.03	35	39.19	47.82	-4.94 [-26.34, 16.46]	3.57
T. Lin 2022	46	556.4	1080.96	56	323.1	268.2	233.30 [-59.95, 526.55]	0.05
X. Wang 2022	12	9.8	.4	75	5	4	4.80 [2.52, 7.08]	6.08
Zitao Wang 2022	162	63	20.59	104	14.63	17.68	48.37 [43.57, 53.17]	5.91
Yunyun Xu 2022	284	33.79	45.32	1,215	34.7	42.61	-0.91 [-6.48, 4.66]	5.84
Gökhan Sargına 2022	33	22.4	21.23	50	5.43	6.64	16.97 [10.68, 23.26]	5.77
Li Xu 2021	58	328	443	29	392	355	-64.00 [-249.47, 121.47]	0.11
Amira H. Allama 2020	15	394	485	30	402	563	-8.00 [-341.97, 325.97]	0.03
Xinyu Wu 2020	58	328	443	29	392	355	-64.00 [-249.47, 121.47]	0.11
Wei Zhou 2020	20	24.76	29.48	20	16.06	17.16	8.70 [-6.25, 23.65]	4.54
T Matsuo 2019	26	15	17	286	5.9	11.7	9.10 [4.20, 14.00]	5.90
Na-Lin Lai 2019	100	32.72	36.74	100	33.36	37.49	-0.64 [-10.93, 9.65]	5.26
Ji Ae Yang 2019	77	29.5	35.3	231	19.1	29.5	10.40 [2.39, 18.41]	5.57
Gökhan SARGIN 2018	43	33.17	42.95	40	32.97	37.6	0.20 [-17.22, 17.62]	4.16
Jun-Xiang Wang 2015	83	57.7	55.81	461	40.01	44.7	17.69 [6.81, 28.57]	5.17
Yufeng Yin 2014	71	426	754	214	394	485	32.00 [-119.29, 183.29]	0.17
Koji MANIWA 2000	32	56	52	38	55	57	1.00 [-24.76, 26.76]	3.01
Heterogeneity: τ^2 = 273.16, I^2 = 94.61%, H^2 = 18.55							9.98 [0.06, 19.89]	
Test of $\theta_i = \theta_j$: Q(16) = 296.75, p = 0.00								
Test of θ = 0: z = 1.97, p = 0.05								
**Europe**								
Verónica Pulito-Cueto 2022	21	11	11	25	5	5	6.00 [1.19, 10.81]	5.91
Verónica Pulito-Cueto 2020	20	11	11	25	5	5	6.00 [1.17, 10.83]	5.91
Salaffi, Fausto 2019	29	149.4	144	122	157.7	279.6	-8.30 [-113.41, 96.81]	0.34
Heterogeneity: τ^2 = 0.00, I^2 = 0.00%, H^2 = 1.00							5.98 [2.58, 9.39]	
Test of $\theta_i = \theta_j$: Q(2) = 0.07, p = 0.97								
Test of θ = 0: z = 3.45, p = 0.00								
**Overall**							9.65 [3.39, 15.92]	
Heterogeneity: τ^2 = 166.65, I^2 = 92.43%, H^2 = 13.20								
Test of $\theta_i = \theta_j$: Q(24) = 316.90, p = 0.00								
Test of θ = 0: z = 3.02, p = 0.00								
Test of group differences: Q_b(3) = 1.12, p = 0.77								

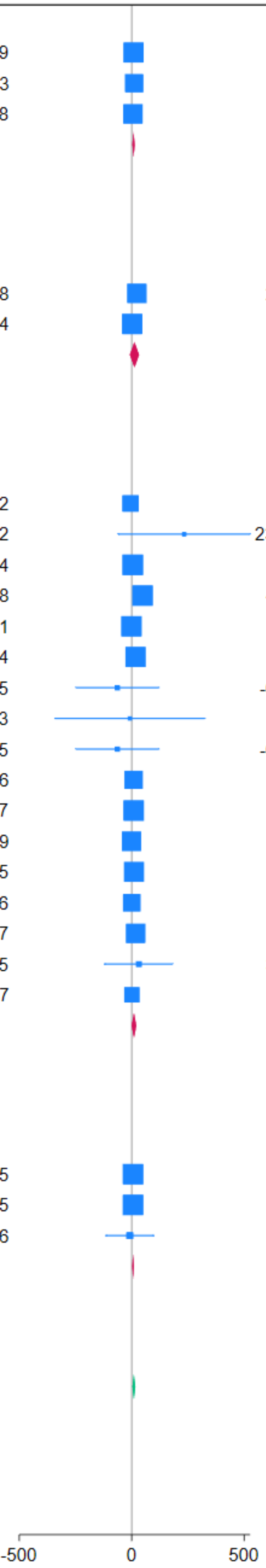

Random-effects DerSimonian–Laird model

Association Between CRP and RA-ILD: Subgroup Analysis by Region

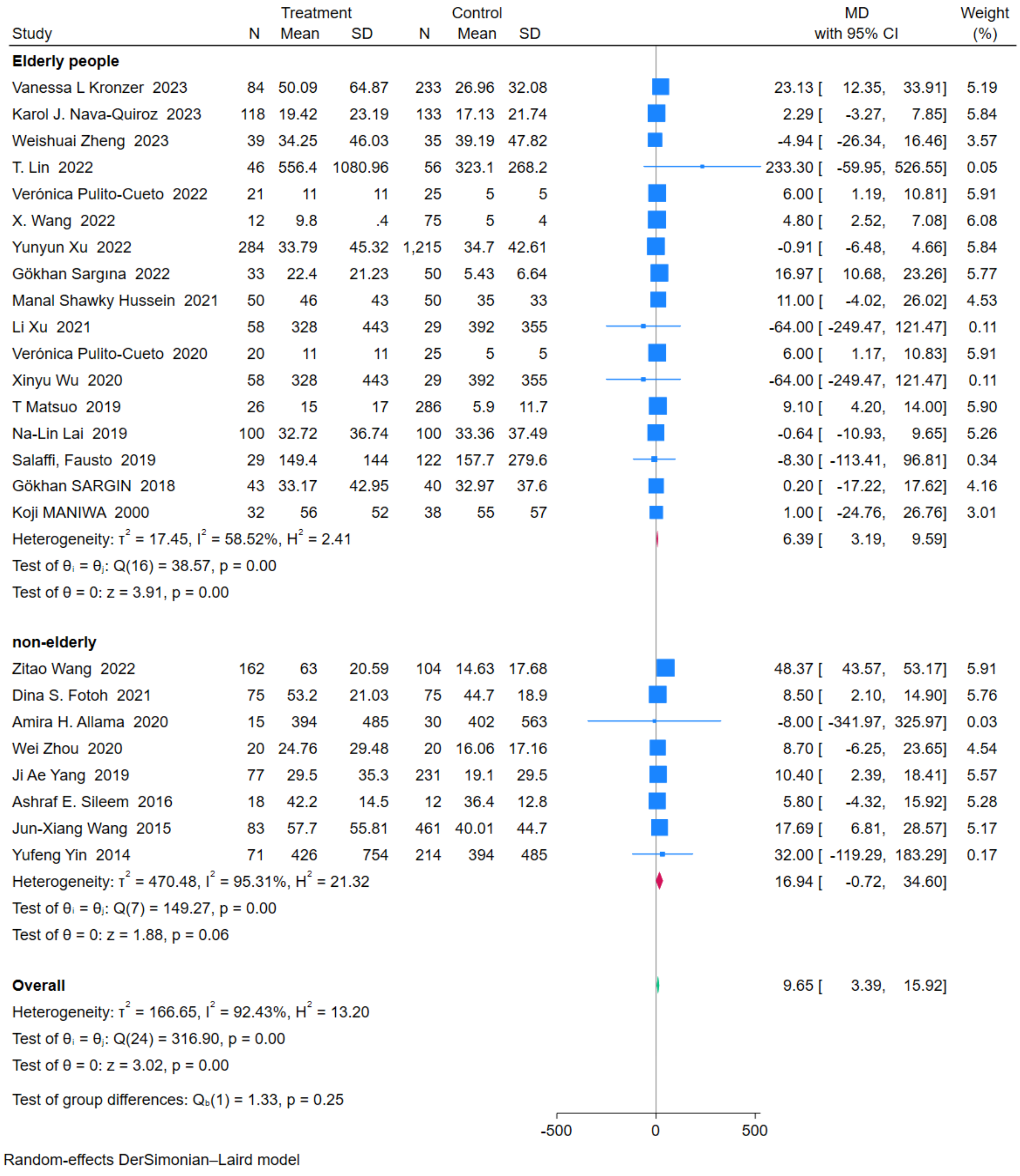

Association Between CRP and RA-ILD: Subgroup Analysis by Age

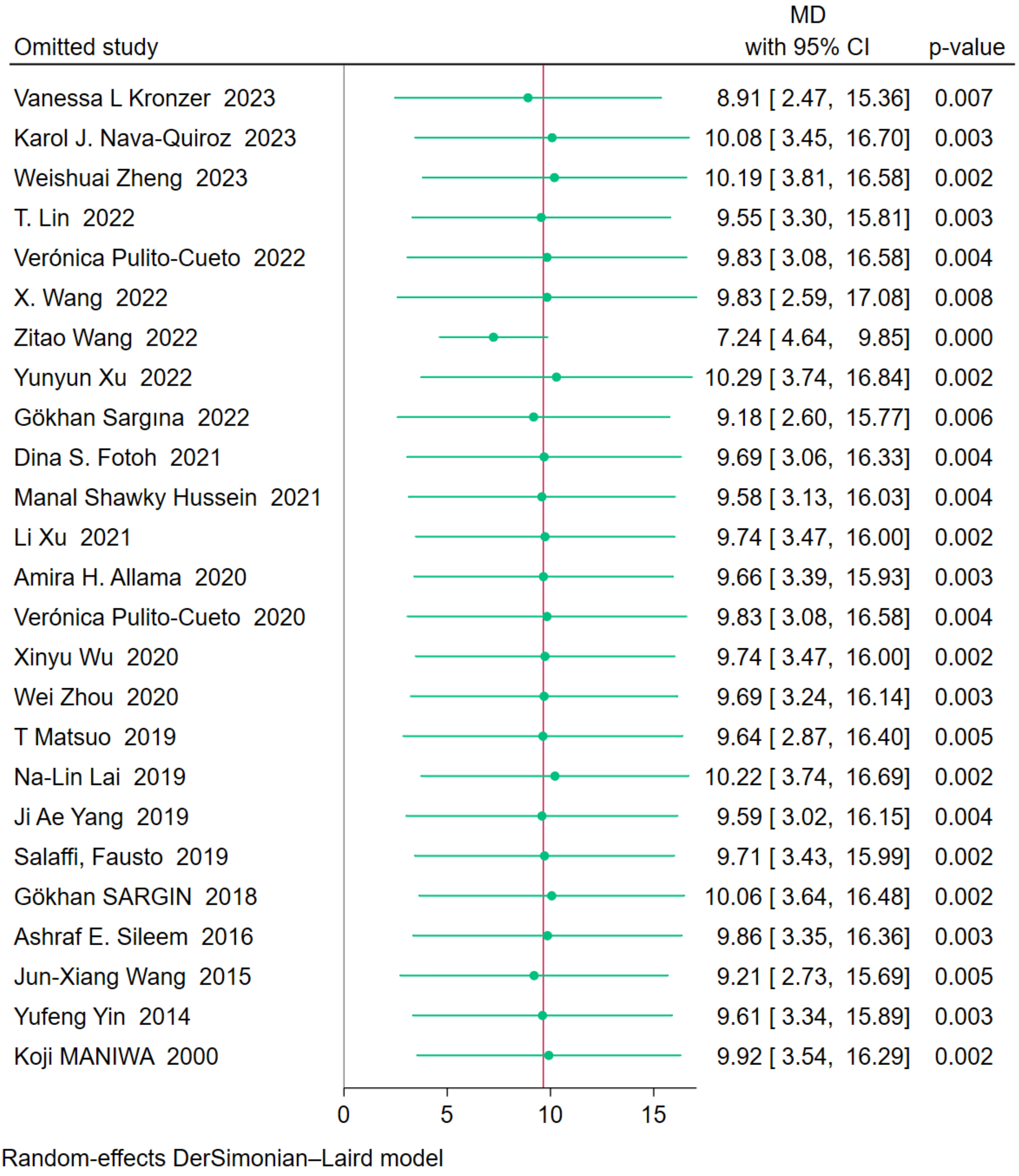

Sensitivity Analysis of CRP as a Biomarker for RA-ILD

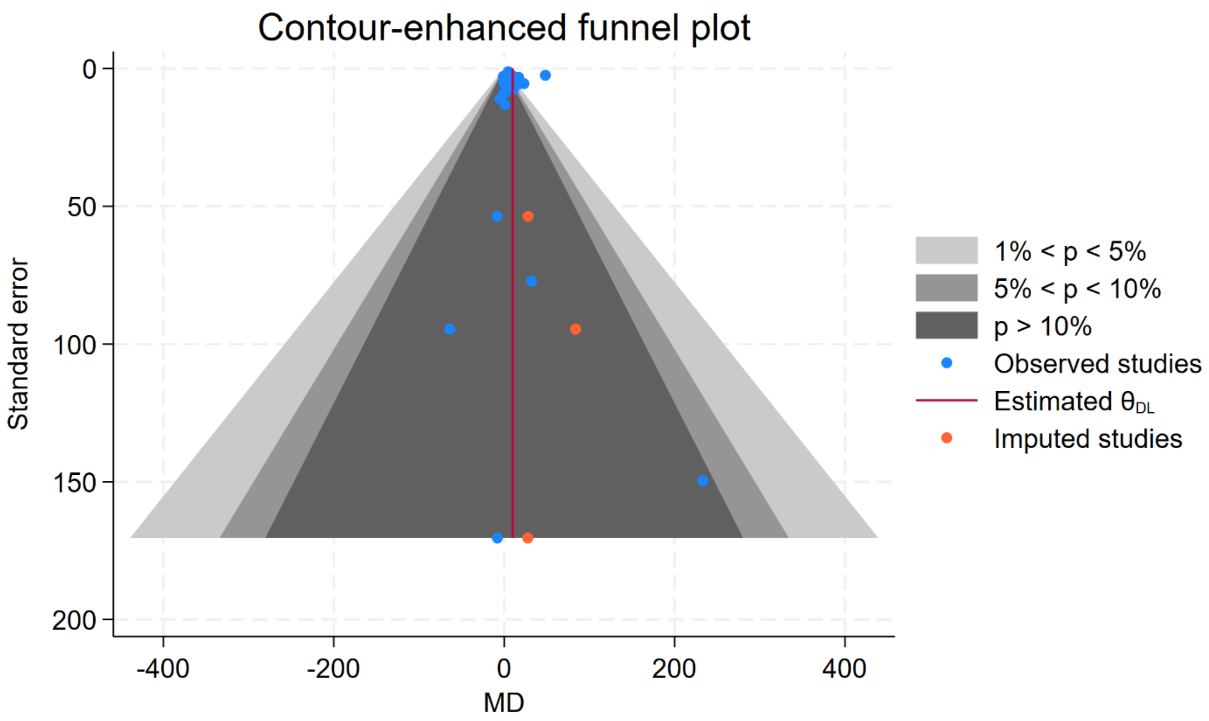

Snip-and-fill funnel plot of CRP as a Biomarker for RA-ILD

2 ESR

Study	Treatment N	Treatment Mean	Treatment SD	Control N	Control Mean	Control SD	MD with 95% CI	Weight (%)
**Africa**								
Dina S. Fotoh 2021	75	83.02	22.6	75	75.6	15.7	7.42 [1.19, 13.65]	4.77
Manal Shawky Hussein 2021	50	45.5	26.6	50	30.3	22.4	15.20 [5.56, 24.84]	4.03
Heterogeneity: τ^2 = 13.12, I^2 = 43.36%, H^2 = 1.77							10.40 [2.99, 17.82]	
Test of $\theta_i = \theta_j$: Q(1) = 1.77, p = 0.18								
Test of $\theta = 0$: z = 2.75, p = 0.01								
**America**								
Karol J. Nava-Quiroz 2023	118	30.89	9.01	133	30.58	17.61	0.31 [-3.22, 3.84]	5.24
José Félix Restrepo 2015	69	57	32	563	39	25	18.00 [11.54, 24.46]	4.72
Heterogeneity: τ^2 = 149.41, I^2 = 95.49%, H^2 = 22.18							8.94 [-8.39, 26.27]	
Test of $\theta_i = \theta_j$: Q(1) = 22.18, p = 0.00								
Test of $\theta = 0$: z = 1.01, p = 0.31								
**Asia**								
Weishuai Zheng 2023	39	28.57	25.64	35	39.73	34.04	-11.16 [-24.81, 2.49]	3.18
T. Lin 2022	46	49.13	24.76	56	41.96	25.54	7.17 [-2.66, 17.00]	3.99
X. Wang 2022	12	33.3	12.6	75	22.8	12.5	10.50 [2.87, 18.13]	4.48
Zitao Wang 2022	162	50.31	8.51	104	28.59	24.28	21.72 [17.64, 25.80]	5.16
Yunyun Xu 2022	284	51.48	34.15	1,215	51.73	32.27	-0.25 [-4.47, 3.97]	5.13
Li Xu 2021	58	54.2	34.3	29	47.1	30.6	7.10 [-7.67, 21.87]	2.97
Amira H. Allama 2020	15	56.2	34.5	30	56.1	35	0.10 [-21.49, 21.69]	1.95
Xinyu Wu 2020	58	54.2	34.3	29	47.1	30.6	7.10 [-7.67, 21.87]	2.97
Wei Zhou 2020	20	42.7	26.75	20	38.6	26.58	4.10 [-12.43, 20.63]	2.66
Qiang Chen 2019	103	49.31	23.36	198	42.06	26.09	7.25 [1.25, 13.25]	4.81
T Matsuo 2019	26	36.3	27	286	26.2	21.1	10.10 [1.41, 18.79]	4.24
Gökhan Sargına 2022	33	46.7	20.1	50	33.5	15.9	13.20 [5.43, 20.97]	4.44
Na Wang 2020	45	22.2	15.5	51	36	26.7	-13.80 [-22.69, -4.91]	4.20
Na-Lin Lai 2019	100	67.84	34.42	100	62.88	38.53	4.96 [-5.17, 15.09]	3.92
Ji Ae Yang 2019	77	59.9	37.3	231	44.9	35.1	15.00 [5.80, 24.20]	4.13
Jun-Xiang Wang 2015	83	75.99	31.46	461	65.32	31.63	10.67 [3.28, 18.06]	4.53
Yufeng Yin 2014	71	56.6	33.2	214	56.1	35	0.50 [-8.78, 9.78]	4.11
Koji MANIWA 2000	32	81	28	38	71	27	10.00 [-2.91, 22.91]	3.33
Heterogeneity: τ^2 = 80.76, I^2 = 82.59%, H^2 = 5.74							6.31 [1.49, 11.12]	
Test of $\theta_i = \theta_j$: Q(17) = 97.63, p = 0.00								
Test of $\theta = 0$: z = 2.57, p = 0.01								
**Europe**								
Verónica Pulito-Cueto 2022	21	22.8	27.2	25	14.4	12.4	8.40 [-3.49, 20.29]	3.54
Verónica Pulito-Cueto 2020	20	22.8	27.2	25	14.4	12.4	8.40 [-3.55, 20.35]	3.53
Salaffi, Fausto 2019	29	35.93	23.75	122	39.13	24.4	-3.20 [-13.03, 6.63]	3.99
Heterogeneity: τ^2 = 17.63, I^2 = 35.12%, H^2 = 1.54							3.88 [-4.12, 11.88]	
Test of $\theta_i = \theta_j$: Q(2) = 3.08, p = 0.21								
Test of $\theta = 0$: z = 0.95, p = 0.34								
**Overall**							6.77 [3.02, 10.53]	
Heterogeneity: τ^2 = 66.73, I^2 = 81.75%, H^2 = 5.48								
Test of $\theta_i = \theta_j$: Q(24) = 131.48, p = 0.00								
Test of $\theta = 0$: z = 3.54, p = 0.00								
Test of group differences: $Q_b(3)$ = 1.52, p = 0.68								

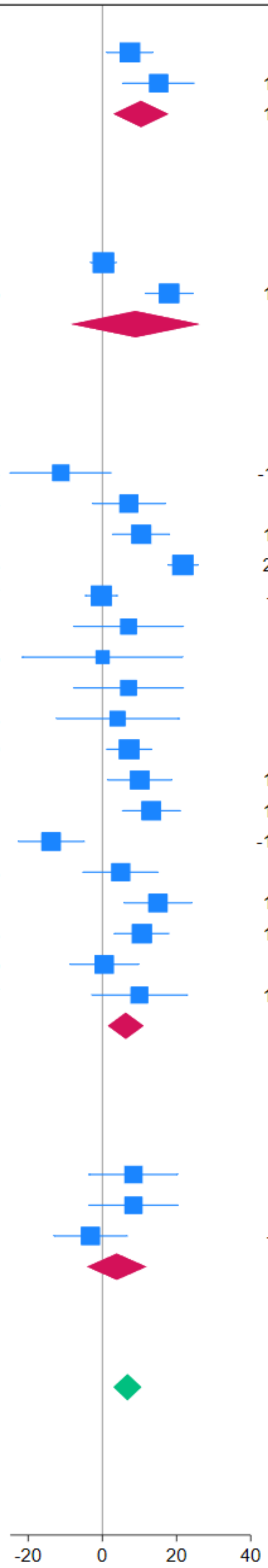

Random-effects DerSimonian–Laird model

Association Between ESR and RA-ILD: Subgroup Analysis by Region

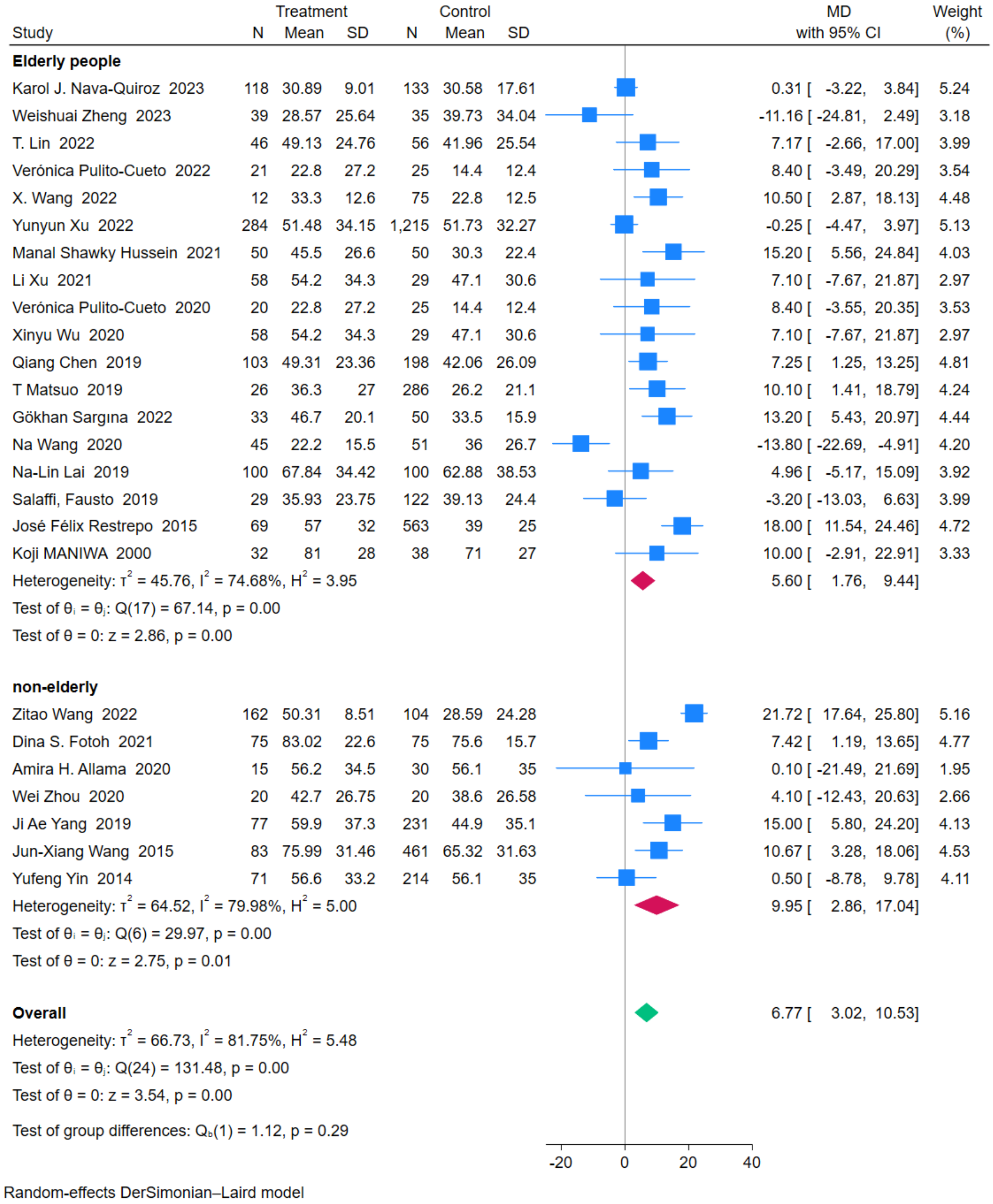

Association Between ESR and RA-ILD: Subgroup Analysis by Age

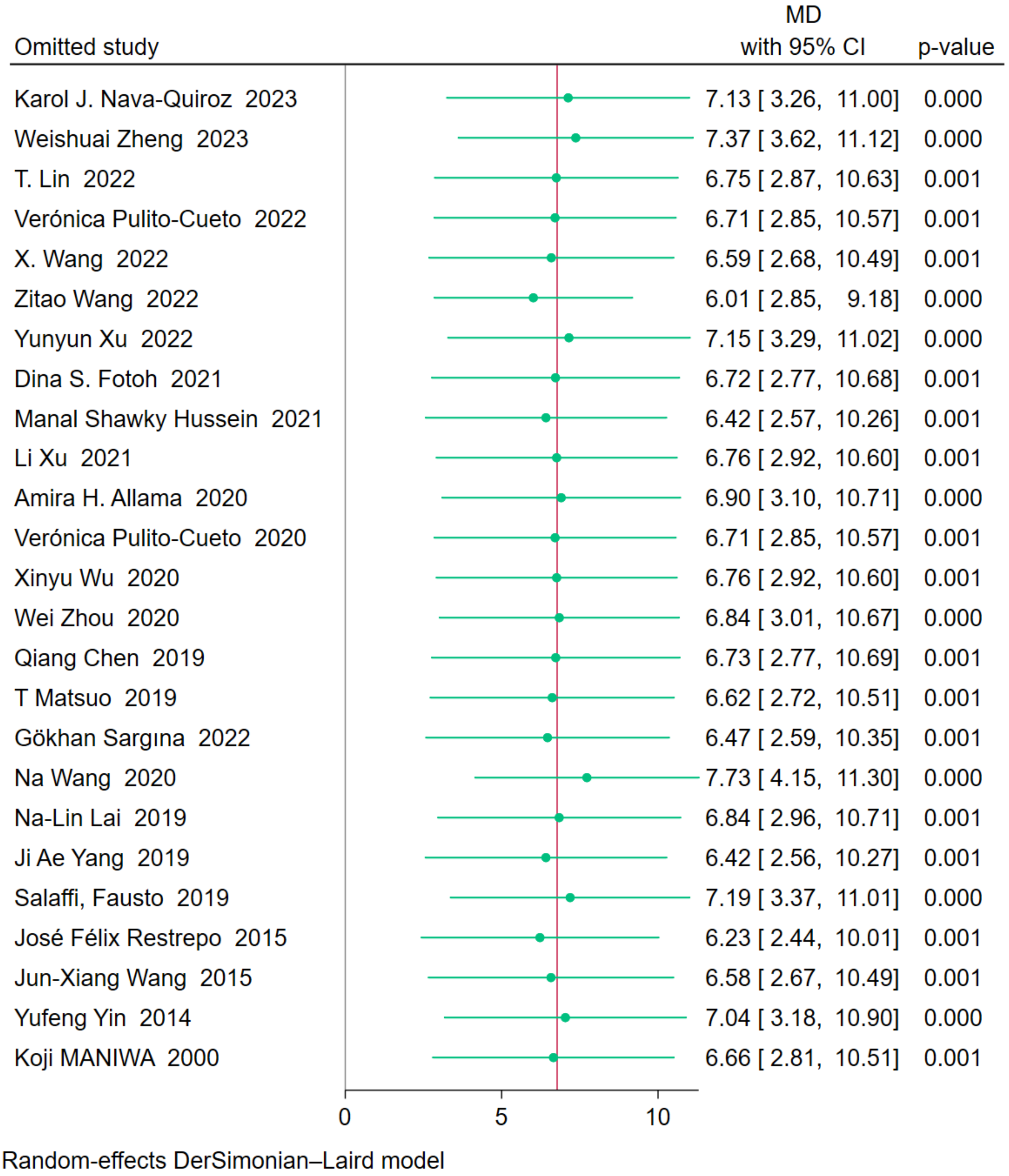

Sensitivity Analysis of ESR as a Biomarker for RA-ILDextensive lung involvement

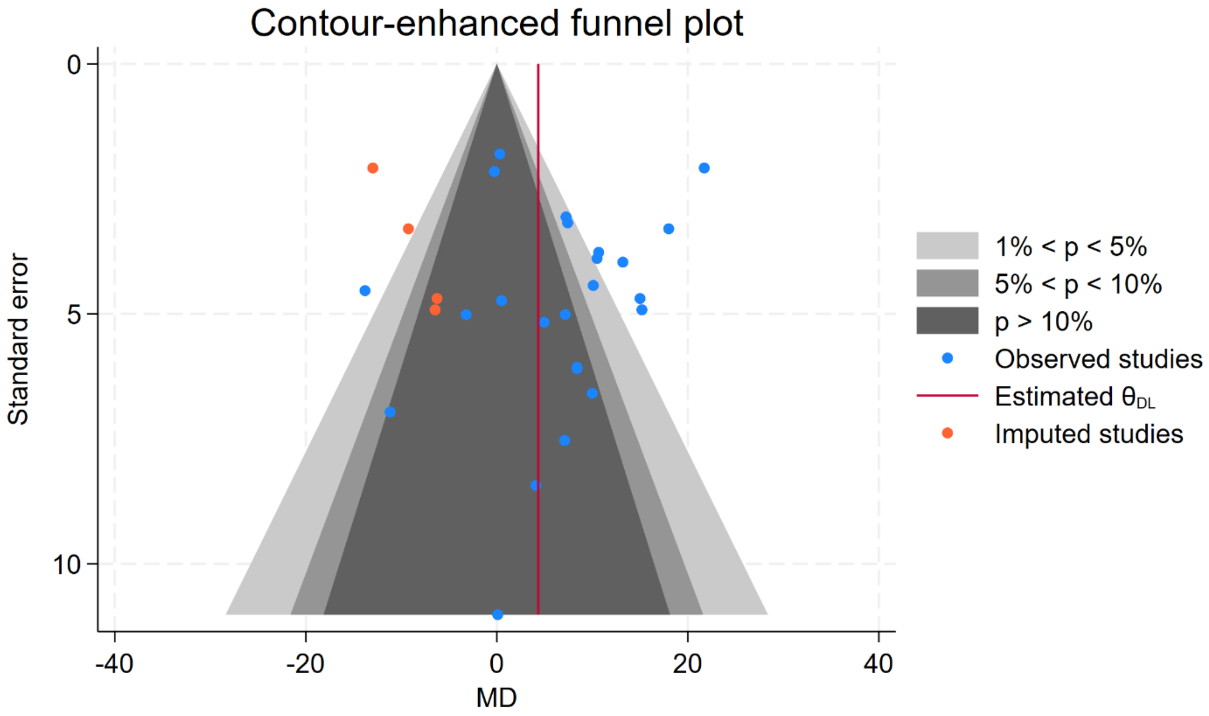

Snip-and-fill funnel plot of ESR as a Biomarker for RA-ILD

3 Anti-CCP Antibody

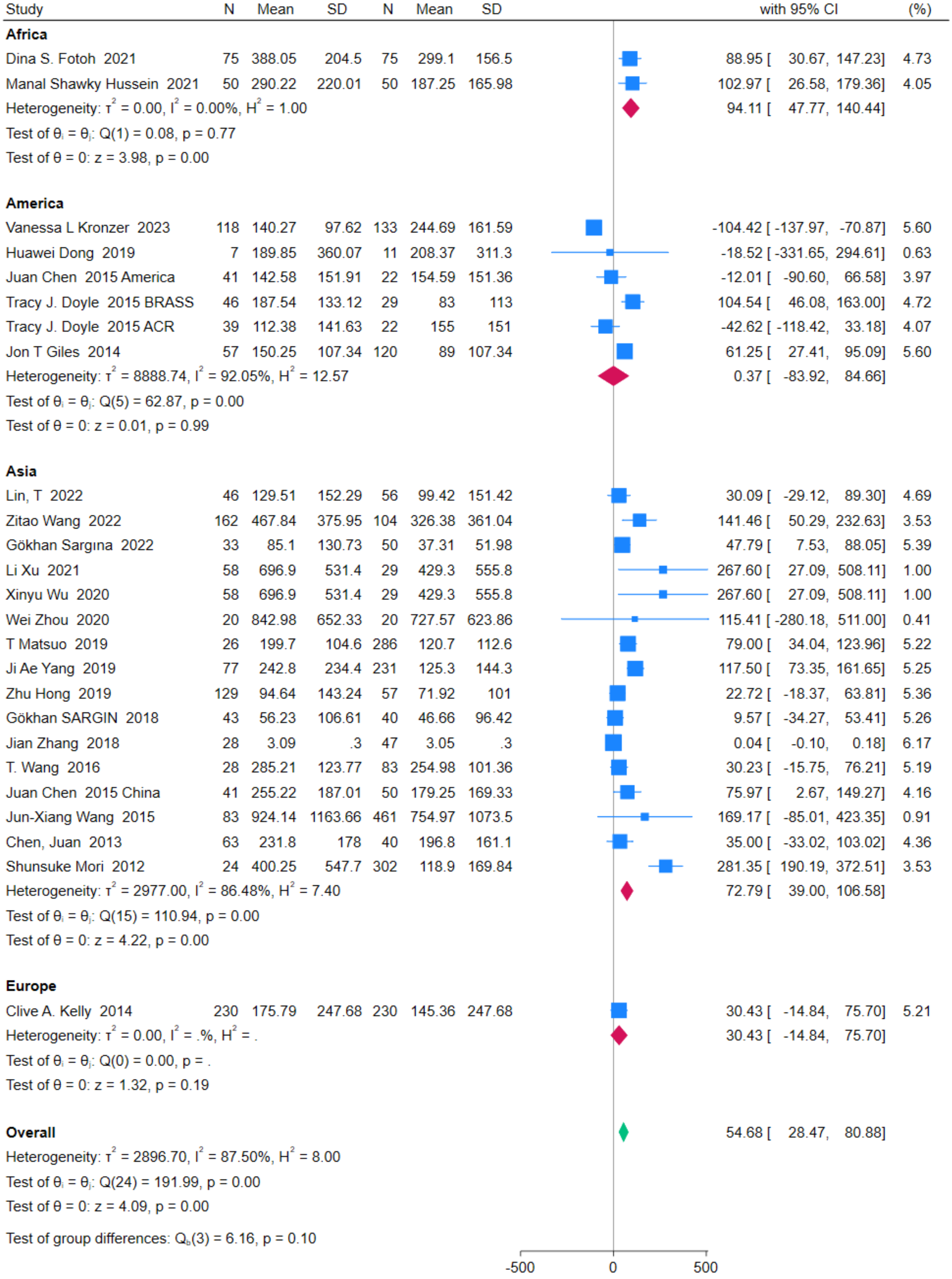

Study	Treatment N	Treatment Mean	Treatment SD	Control N	Control Mean	Control SD	MD with 95% CI	Weight (%)
**Africa**								
Dina S. Fotoh 2021	75	388.05	204.5	75	299.1	156.5	88.95 [30.67, 147.23]	4.73
Manal Shawky Hussein 2021	50	290.22	220.01	50	187.25	165.98	102.97 [26.58, 179.36]	4.05
Heterogeneity: τ^2 = 0.00, I^2 = 0.00%, H^2 = 1.00							94.11 [47.77, 140.44]	
Test of $\theta_i = \theta_j$: Q(1) = 0.08, p = 0.77								
Test of θ = 0: z = 3.98, p = 0.00								
**America**								
Vanessa L Kronzer 2023	118	140.27	97.62	133	244.69	161.59	-104.42 [-137.97, -70.87]	5.60
Huawei Dong 2019	7	189.85	360.07	11	208.37	311.3	-18.52 [-331.65, 294.61]	0.63
Juan Chen 2015 America	41	142.58	151.91	22	154.59	151.36	-12.01 [-90.60, 66.58]	3.97
Tracy J. Doyle 2015 BRASS	46	187.54	133.12	29	83	113	104.54 [46.08, 163.00]	4.72
Tracy J. Doyle 2015 ACR	39	112.38	141.63	22	155	151	-42.62 [-118.42, 33.18]	4.07
Jon T Giles 2014	57	150.25	107.34	120	89	107.34	61.25 [27.41, 95.09]	5.60
Heterogeneity: τ^2 = 8888.74, I^2 = 92.05%, H^2 = 12.57							0.37 [-83.92, 84.66]	
Test of $\theta_i = \theta_j$: Q(5) = 62.87, p = 0.00								
Test of θ = 0: z = 0.01, p = 0.99								
**Asia**								
Lin, T 2022	46	129.51	152.29	56	99.42	151.42	30.09 [-29.12, 89.30]	4.69
Zitao Wang 2022	162	467.84	375.95	104	326.38	361.04	141.46 [50.29, 232.63]	3.53
Gökhan Sargına 2022	33	85.1	130.73	50	37.31	51.98	47.79 [7.53, 88.05]	5.39
Li Xu 2021	58	696.9	531.4	29	429.3	555.8	267.60 [27.09, 508.11]	1.00
Xinyu Wu 2020	58	696.9	531.4	29	429.3	555.8	267.60 [27.09, 508.11]	1.00
Wei Zhou 2020	20	842.98	652.33	20	727.57	623.86	115.41 [-280.18, 511.00]	0.41
T Matsuo 2019	26	199.7	104.6	286	120.7	112.6	79.00 [34.04, 123.96]	5.22
Ji Ae Yang 2019	77	242.8	234.4	231	125.3	144.3	117.50 [73.35, 161.65]	5.25
Zhu Hong 2019	129	94.64	143.24	57	71.92	101	22.72 [-18.37, 63.81]	5.36
Gökhan SARGIN 2018	43	56.23	106.61	40	46.66	96.42	9.57 [-34.27, 53.41]	5.26
Jian Zhang 2018	28	3.09	.3	47	3.05	.3	0.04 [-0.10, 0.18]	6.17
T. Wang 2016	28	285.21	123.77	83	254.98	101.36	30.23 [-15.75, 76.21]	5.19
Juan Chen 2015 China	41	255.22	187.01	50	179.25	169.33	75.97 [2.67, 149.27]	4.16
Jun-Xiang Wang 2015	83	924.14	1163.66	461	754.97	1073.5	169.17 [-85.01, 423.35]	0.91
Chen, Juan 2013	63	231.8	178	40	196.8	161.1	35.00 [-33.02, 103.02]	4.36
Shunsuke Mori 2012	24	400.25	547.7	302	118.9	169.84	281.35 [190.19, 372.51]	3.53
Heterogeneity: τ^2 = 2977.00, I^2 = 86.48%, H^2 = 7.40							72.79 [39.00, 106.58]	
Test of $\theta_i = \theta_j$: Q(15) = 110.94, p = 0.00								
Test of θ = 0: z = 4.22, p = 0.00								
**Europe**								
Clive A. Kelly 2014	230	175.79	247.68	230	145.36	247.68	30.43 [-14.84, 75.70]	5.21
Heterogeneity: τ^2 = 0.00, I^2 = .%, H^2 = .							30.43 [-14.84, 75.70]	
Test of $\theta_i = \theta_j$: Q(0) = 0.00, p = .								
Test of θ = 0: z = 1.32, p = 0.19								
**Overall**							54.68 [28.47, 80.88]	
Heterogeneity: τ^2 = 2896.70, I^2 = 87.50%, H^2 = 8.00								
Test of $\theta_i = \theta_j$: Q(24) = 191.99, p = 0.00								
Test of θ = 0: z = 4.09, p = 0.00								
Test of group differences: Q_b(3) = 6.16, p = 0.10								

Random-effects DerSimonian–Laird model

Association Between Anti-CCP Antibody and RA-ILD: Subgroup Analysis by Region

Study	Treatment N	Treatment Mean	Treatment SD	Control N	Control Mean	Control SD	MD with 95% CI	Weight (%)
**Elderly people**								
Vanessa L Kronzer 2023	118	140.27	97.62	133	244.69	161.59	-104.42 [-137.97, -70.87]	5.60
Lin, T 2022	46	129.51	152.29	56	99.42	151.42	30.09 [-29.12, 89.30]	4.69
Gökhan Sargına 2022	33	85.1	130.73	50	37.31	51.98	47.79 [7.53, 88.05]	5.39
Manal Shawky Hussein 2021	50	290.22	220.01	50	187.25	165.98	102.97 [26.58, 179.36]	4.05
Li Xu 2021	58	696.9	531.4	29	429.3	555.8	267.60 [27.09, 508.11]	1.00
Xinyu Wu 2020	58	696.9	531.4	29	429.3	555.8	267.60 [27.09, 508.11]	1.00
T Matsuo 2019	26	199.7	104.6	286	120.7	112.6	79.00 [34.04, 123.96]	5.22
Zhu Hong 2019	129	94.64	143.24	57	71.92	101	22.72 [-18.37, 63.81]	5.36
Gökhan SARGIN 2018	43	56.23	106.61	40	46.66	96.42	9.57 [-34.27, 53.41]	5.26
T. Wang 2016	28	285.21	123.77	83	254.98	101.36	30.23 [-15.75, 76.21]	5.19
Juan Chen 2015 America	41	142.58	151.91	22	154.59	151.36	-12.01 [-90.60, 66.58]	3.97
Tracy J. Doyle 2015 BRASS	46	187.54	133.12	29	83	113	104.54 [46.08, 163.00]	4.72
Tracy J. Doyle 2015 ACR	39	112.38	141.63	22	155	151	-42.62 [-118.42, 33.18]	4.07
Clive A. Kelly 2014	230	175.79	247.68	230	145.36	247.68	30.43 [-14.84, 75.70]	5.21
Shunsuke Mori 2012	24	400.25	547.7	302	118.9	169.84	281.35 [190.19, 372.51]	3.53
Heterogeneity: $\tau^2 = 5622.18$, $I^2 = 88.09\%$, $H^2 = 8.39$							51.27 [8.51, 94.04]	
Test of $\theta_i = \theta_j$: Q(14) = 117.52, p = 0.00								
Test of $\theta = 0$: z = 2.35, p = 0.02								
**non-elderly**								
Zitao Wang 2022	162	467.84	375.95	104	326.38	361.04	141.46 [50.29, 232.63]	3.53
Dina S. Fotoh 2021	75	388.05	204.5	75	299.1	156.5	88.95 [30.67, 147.23]	4.73
Wei Zhou 2020	20	842.98	652.33	20	727.57	623.86	115.41 [-280.18, 511.00]	0.41
Ji Ae Yang 2019	77	242.8	234.4	231	125.3	144.3	117.50 [73.35, 161.65]	5.25
Huawei Dong 2019	7	189.85	360.07	11	208.37	311.3	-18.52 [-331.65, 294.61]	0.63
Jian Zhang 2018	28	3.09	.3	47	3.05	.3	0.04 [-0.10, 0.18]	6.17
Juan Chen 2015 China	41	255.22	187.01	50	179.25	169.33	75.97 [2.67, 149.27]	4.16
Jun-Xiang Wang 2015	83	924.14	1163.66	461	754.97	1073.5	169.17 [-85.01, 423.35]	0.91
Jon T Giles 2014	57	150.25	107.34	120	89	107.34	61.25 [27.41, 95.09]	5.60
Chen, Juan 2013	63	231.8	178	40	196.8	161.1	35.00 [-33.02, 103.02]	4.36
Heterogeneity: $\tau^2 = 3268.02$, $I^2 = 86.18\%$, $H^2 = 7.24$							71.53 [25.61, 117.46]	
Test of $\theta_i = \theta_j$: Q(9) = 65.12, p = 0.00								
Test of $\theta = 0$: z = 3.05, p = 0.00								
**Overall**							54.68 [28.47, 80.88]	
Heterogeneity: $\tau^2 = 2896.70$, $I^2 = 87.50\%$, $H^2 = 8.00$								
Test of $\theta_i = \theta_j$: Q(24) = 191.99, p = 0.00								
Test of $\theta = 0$: z = 4.09, p = 0.00								
Test of group differences: $Q_b(1) = 0.40$, p = 0.53								

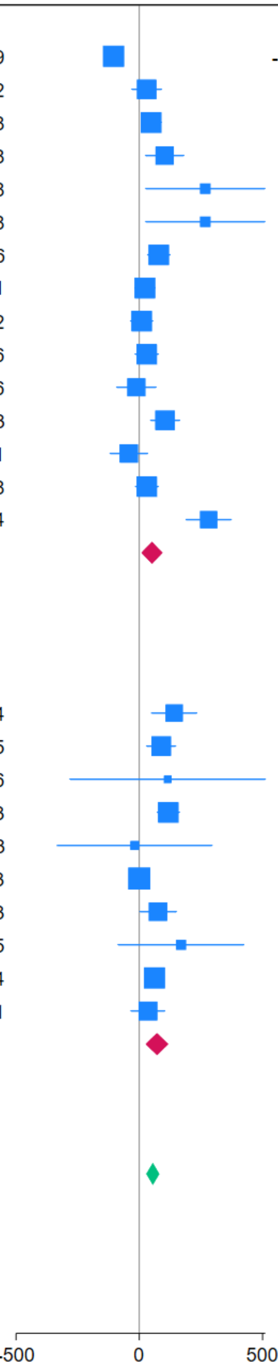

Random-effects DerSimonian–Laird model

Association Between Anti-CCP Antibody and RA-ILD: Subgroup Analysis by Age

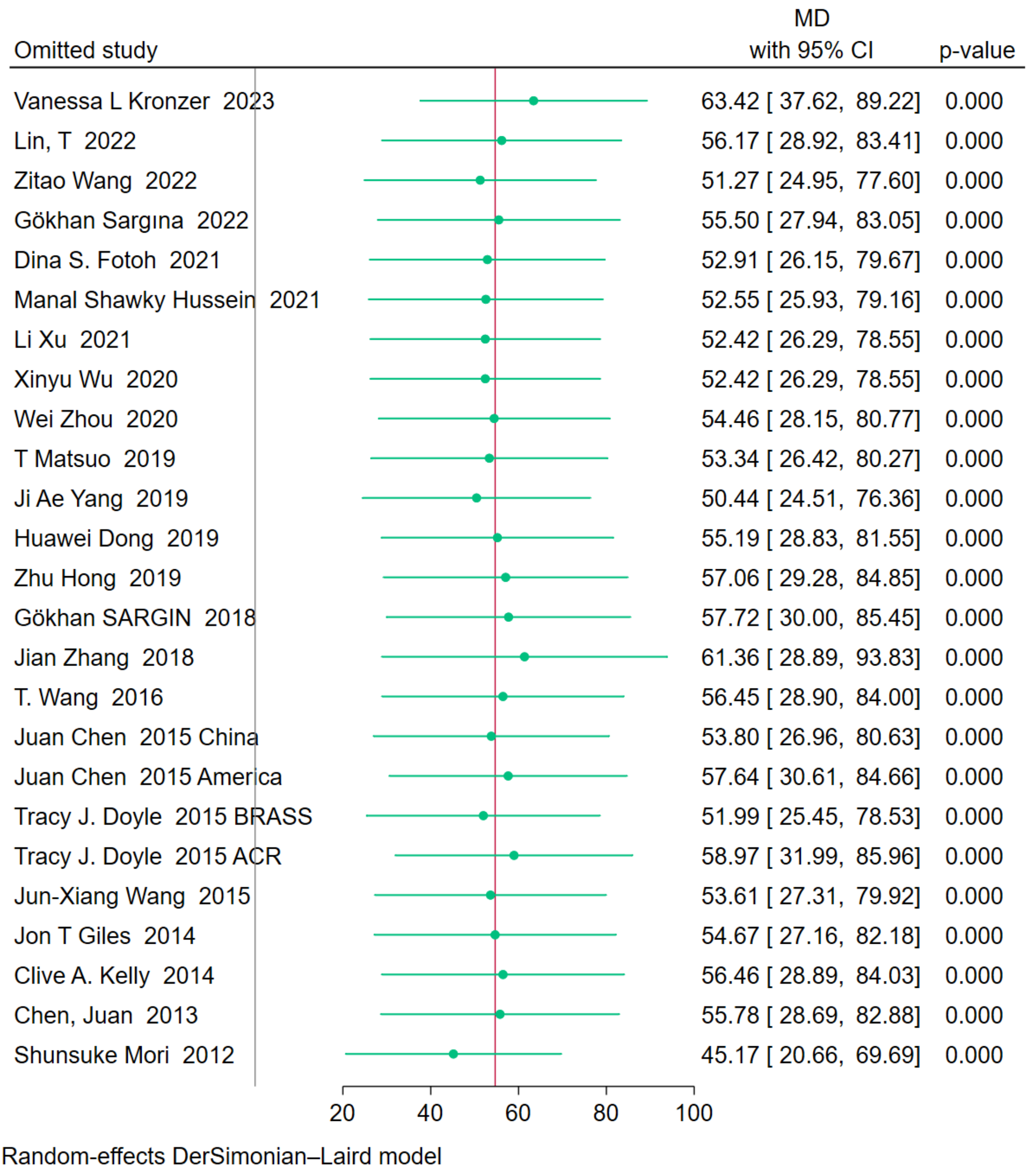

Sensitivity Analysis of Anti-CCP Antibody as a Biomarker for RA-ILD FVC

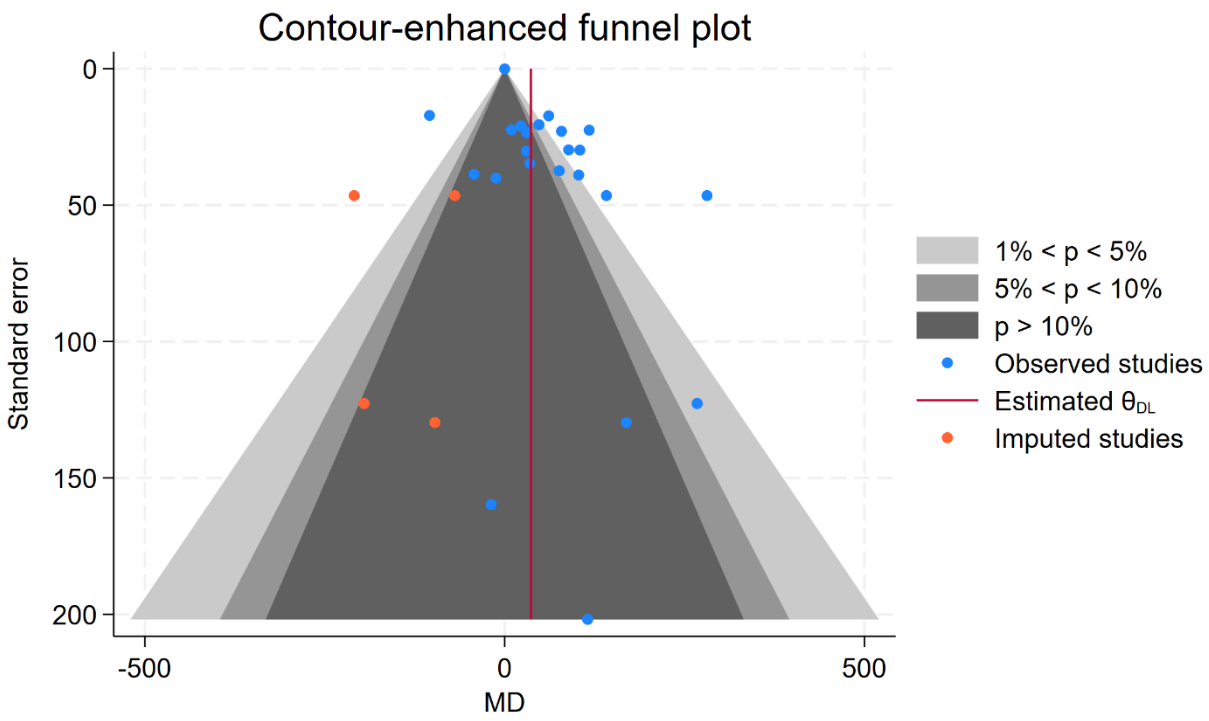

Snip-and-fill funnel plot of Anti-CCP Antibody as a Biomarker for RA-ILD

4 RF

Study	Treatment N	Treatment Mean	Treatment SD	Control N	Control Mean	Control SD	MD with 95% CI	Weight (%)
**Africa**								
Dina S. Fotoh 2021	75	103.1	54.4	75	85.9	42.01	17.20 [1.64, 32.76]	5.82
Manal Shawky Hussein 2021	50	194.76	160.23	50	150.63	128.61	44.13 [-12.82, 101.08]	5.47
Heterogeneity: τ^2 = 0.00, I^2 = 0.00%, H^2 = 1.00							19.07 [4.06, 34.08]	
Test of $\theta_i = \theta_j$: Q(1) = 0.80, p = 0.37								
Test of θ = 0: z = 2.49, p = 0.01								
**America**								
Huawei Dong 2019	7	206.84	466.62	11	37.7	60.23	169.14 [-105.37, 443.65]	2.26
Juan Chen 2015 America	41	409.65	708.2	22	143.75	357	265.90 [-50.35, 582.15]	1.89
Tracy J. Doyle 2015 BRASS	46	293.52	546.9	29	114	186	179.52 [-27.09, 386.13]	3.08
Tracy J. Doyle 2015 ACR	39	336.31	636.22	22	144	357	192.31 [-96.81, 481.43]	2.12
Heterogeneity: τ^2 = 0.00, I^2 = 0.00%, H^2 = 1.00							194.50 [63.94, 325.07]	
Test of $\theta_i = \theta_j$: Q(3) = 0.25, p = 0.97								
Test of θ = 0: z = 2.92, p = 0.00								
**Asia**								
Zitao Wang 2022	162	235.31	182.76	104	103.04	162.14	132.27 [89.17, 175.37]	5.63
Lin, T 2022	46	137	5593	56	93.3	1045	43.70 [-1450.47, 1537.87]	0.12
Yunyun Xu 2022	284	491.68	829.42	1,215	407.36	1085.88	84.32 [-50.32, 218.96]	4.23
Li Xu 2021	58	615.8	1186.5	29	213.8	194.9	402.00 [-33.96, 837.96]	1.17
Hiroshi Furukawa 2020	100	564.6	1302.2	100	154.3	241.2	410.30 [150.73, 669.87]	2.42
Ho Cheol KimID 2020	30	425.28	614.87	54	98.97	129.86	326.31 [156.59, 496.03]	3.64
Xinyu Wu 2020	58	615.8	1186.5	29	213.8	194.9	402.00 [-33.96, 837.96]	1.17
Wei Zhou 2020	20	559.2	785.68	20	531.81	941.6	27.39 [-510.07, 564.85]	0.83
Na Wang 2020	45	372.2	773	51	112.4	121.6	259.80 [44.84, 474.76]	2.97
Zhu Hong 2019	129	124.85	182.94	57	107.27	154.1	17.58 [-36.87, 72.03]	5.50
Na-Lin Lai 2019	100	391.05	413.34	100	221.02	321.53	170.03 [67.39, 272.67]	4.79
Ji Ae Yang 2019	77	521.1	828.3	231	249.3	332.4	271.80 [141.96, 401.64]	4.32
Jian Zhang 2018	28	592.17	68.4	47	318.54	36.2	273.63 [249.97, 297.29]	5.78
Gökhan SARGIN 2018	43	57.7	89.2	40	24.35	44.91	33.35 [2.61, 64.09]	5.73
Song, Seung Taek 2016	8	94.61	105.88	22	34.67	27.1	59.94 [13.08, 106.80]	5.59
Juan Chen 2015 China	41	795.03	1966.34	50	180.63	228.61	614.40 [65.55, 1163.25]	0.80
Jun-Xiang Wang 2015	83	657.81	895.06	461	352.66	589.22	305.15 [154.44, 455.86]	3.96
Chen, Juan 2013	63	555.8	1599.1	40	159.8	206.7	396.00 [-103.05, 895.05]	0.94
Shunsuke Mori 2012	24	232.02	267.94	302	70.77	92.74	161.25 [113.70, 208.80]	5.58
Koji MANIWA 2000	32	534.7	424.6	38	226.2	372.5	308.50 [121.76, 495.24]	3.38
Heterogeneity: τ^2 = 13426.67, I^2 = 91.50%, H^2 = 11.76							194.62 [128.88, 260.35]	
Test of $\theta_i = \theta_j$: Q(19) = 223.48, p = 0.00								
Test of θ = 0: z = 5.80, p = 0.00								
**Europe**								
Raul Castellanos-Moreira 2020	37	105	220.74	142	34	81.48	71.00 [26.40, 115.60]	5.61
Salaffi, Fausto 2019	29	155.62	163.67	122	123.04	197.67	32.58 [-45.06, 110.22]	5.19
Heterogeneity: τ^2 = 0.00, I^2 = 0.00%, H^2 = 1.00							61.47 [22.79, 100.14]	
Test of $\theta_i = \theta_j$: Q(1) = 0.71, p = 0.40								
Test of θ = 0: z = 3.12, p = 0.00								
**Overall**							159.82 [107.06, 212.58]	
Heterogeneity: τ^2 = 12391.51, I^2 = 93.33%, H^2 = 15.00								
Test of $\theta_i = \theta_j$: Q(27) = 404.89, p = 0.00								
Test of θ = 0: z = 5.94, p = 0.00								
Test of group differences: Q_b(3) = 34.48, p = 0.00								

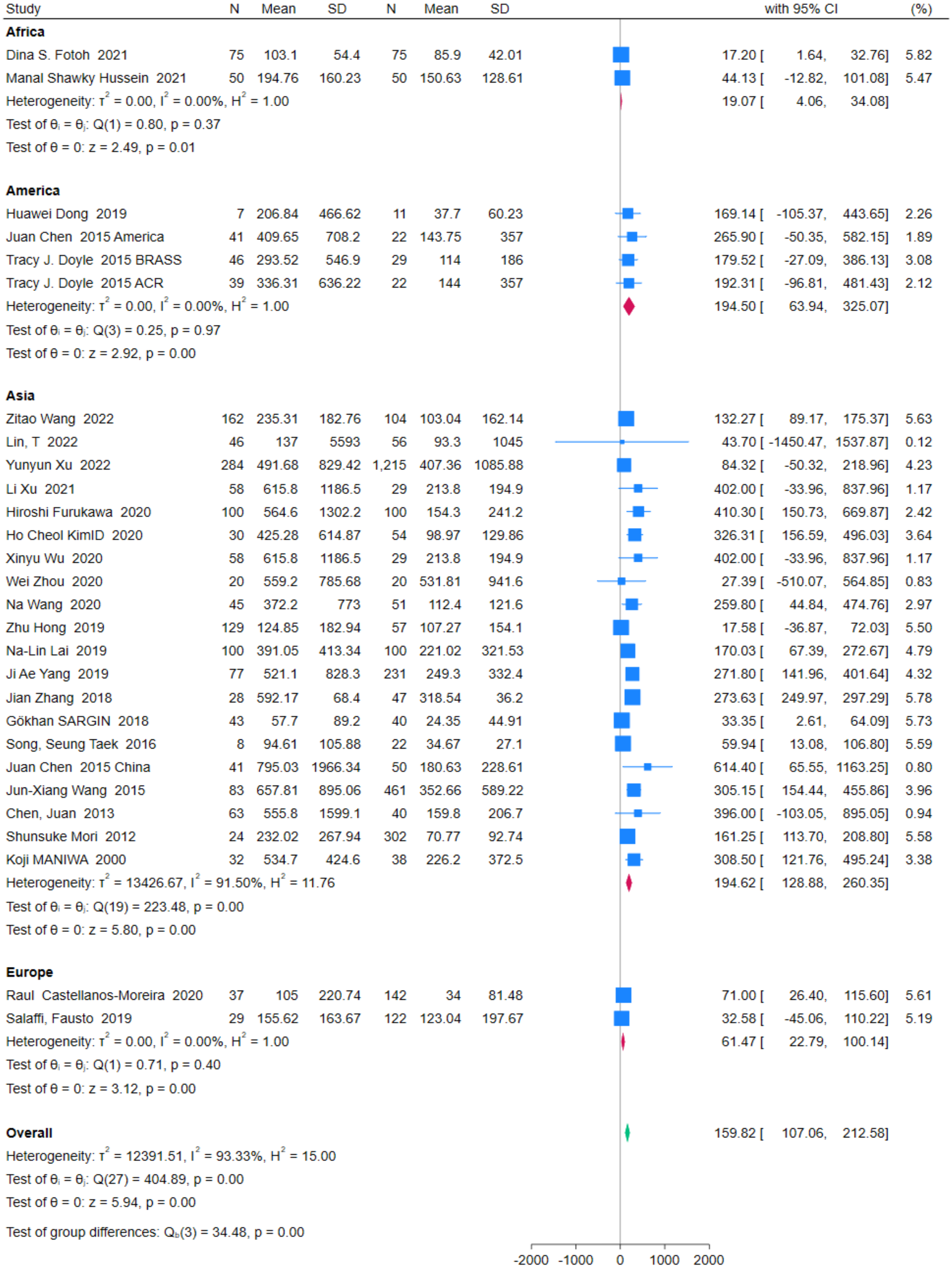

Random-effects DerSimonian–Laird model

Association Between RF and RA-ILD: Subgroup Analysis by Region

Study	Treatment N	Treatment Mean	Treatment SD	Control N	Control Mean	Control SD	MD with 95% CI	Weight (%)
**Elderly people**								
Lin, T 2022	46	137	5593	56	93.3	1045	43.70 [-1450.47, 1537.87]	0.12
Yunyun Xu 2022	284	491.68	829.42	1,215	407.36	1085.88	84.32 [-50.32, 218.96]	4.23
Manal Shawky Hussein 2021	50	194.76	160.23	50	150.63	128.61	44.13 [-12.82, 101.08]	5.47
Li Xu 2021	58	615.8	1186.5	29	213.8	194.9	402.00 [-33.96, 837.96]	1.17
Hiroshi Furukawa 2020	100	564.6	1302.2	100	154.3	241.2	410.30 [150.73, 669.87]	2.42
Ho Cheol KimID 2020	30	425.28	614.87	54	98.97	129.86	326.31 [156.59, 496.03]	3.64
Xinyu Wu 2020	58	615.8	1186.5	29	213.8	194.9	402.00 [-33.96, 837.96]	1.17
Raul Castellanos-Moreira 2020	37	105	220.74	142	34	81.48	71.00 [26.40, 115.60]	5.61
Salaffi, Fausto 2019	29	155.62	163.67	122	123.04	197.67	32.58 [-45.06, 110.22]	5.19
Zhu Hong 2019	129	124.85	182.94	57	107.27	154.1	17.58 [-36.87, 72.03]	5.50
Na-Lin Lai 2019	100	391.05	413.34	100	221.02	321.53	170.03 [67.39, 272.67]	4.79
Gökhan SARGIN 2018	43	57.7	89.2	40	24.35	44.91	33.35 [2.61, 64.09]	5.73
Song, Seung Taek 2016	8	94.61	105.88	22	34.67	27.1	59.94 [13.08, 106.80]	5.59
Juan Chen 2015 America	41	409.65	708.2	22	143.75	357	265.90 [-50.35, 582.15]	1.89
Tracy J. Doyle 2015 BRASS	46	293.52	546.9	29	114	186	179.52 [-27.09, 386.13]	3.08
Tracy J. Doyle 2015 ACR	39	336.31	636.22	22	144	357	192.31 [-96.81, 481.43]	2.12
Shunsuke Mori 2012	24	232.02	267.94	302	70.77	92.74	161.25 [113.70, 208.80]	5.58
Koji MANIWA 2000	32	534.7	424.6	38	226.2	372.5	308.50 [121.76, 495.24]	3.38
Heterogeneity: τ^2 = 3576.90, I^2 = 70.70%, H^2 = 3.41							109.76 [69.01, 150.51]	
Test of $\theta_i = \theta_j$: Q(17) = 58.01, p = 0.00								
Test of θ = 0: z = 5.28, p = 0.00								
**non-elderly**								
Zitao Wang 2022	162	235.31	182.76	104	103.04	162.14	132.27 [89.17, 175.37]	5.63
Dina S. Fotoh 2021	75	103.1	54.4	75	85.9	42.01	17.20 [1.64, 32.76]	5.82
Wei Zhou 2020	20	559.2	785.68	20	531.81	941.6	27.39 [-510.07, 564.85]	0.83
Na Wang 2020	45	372.2	773	51	112.4	121.6	259.80 [44.84, 474.76]	2.97
Huawei Dong 2019	7	206.84	466.62	11	37.7	60.23	169.14 [-105.37, 443.65]	2.26
Ji Ae Yang 2019	77	521.1	828.3	231	249.3	332.4	271.80 [141.96, 401.64]	4.32
Jian Zhang 2018	28	592.17	68.4	47	318.54	36.2	273.63 [249.97, 297.29]	5.78
Juan Chen 2015 China	41	795.03	1966.34	50	180.63	228.61	614.40 [65.55, 1163.25]	0.80
Jun-Xiang Wang 2015	83	657.81	895.06	461	352.66	589.22	305.15 [154.44, 455.86]	3.96
Chen, Juan 2013	63	555.8	1599.1	40	159.8	206.7	396.00 [-103.05, 895.05]	0.94
Heterogeneity: τ^2 = 24576.65, I^2 = 97.34%, H^2 = 37.65							215.63 [96.15, 335.10]	
Test of $\theta_i = \theta_j$: Q(9) = 338.87, p = 0.00								
Test of θ = 0: z = 3.54, p = 0.00								
**Overall**							159.82 [107.06, 212.58]	
Heterogeneity: τ^2 = 12391.51, I^2 = 93.33%, H^2 = 15.00								
Test of $\theta_i = \theta_j$: Q(27) = 404.89, p = 0.00								
Test of θ = 0: z = 5.94, p = 0.00								
Test of group differences: $Q_b(1)$ = 2.70, p = 0.10								

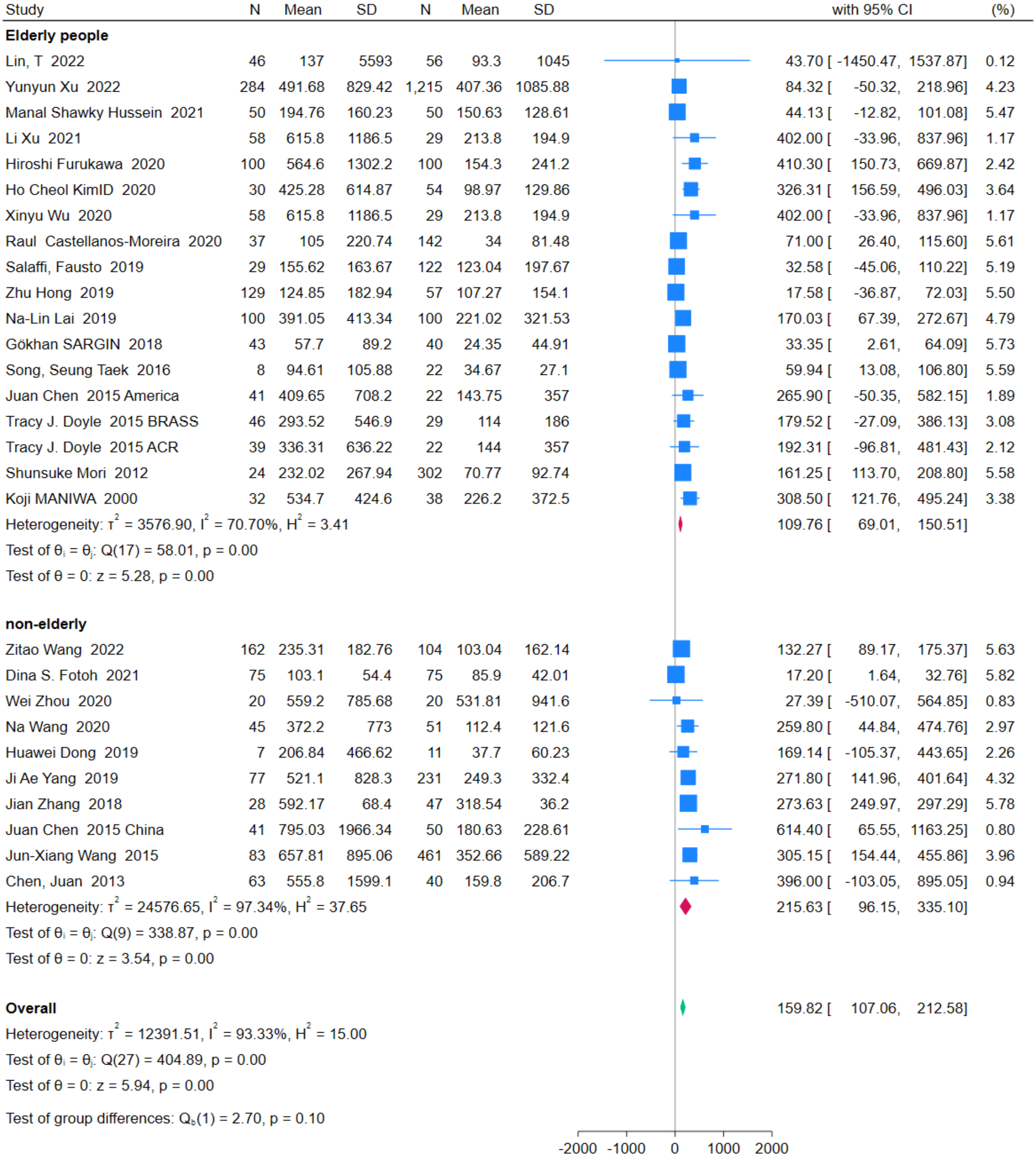

Random-effects DerSimonian–Laird model

Association Between RF and RA-ILD: Subgroup Analysis by Age

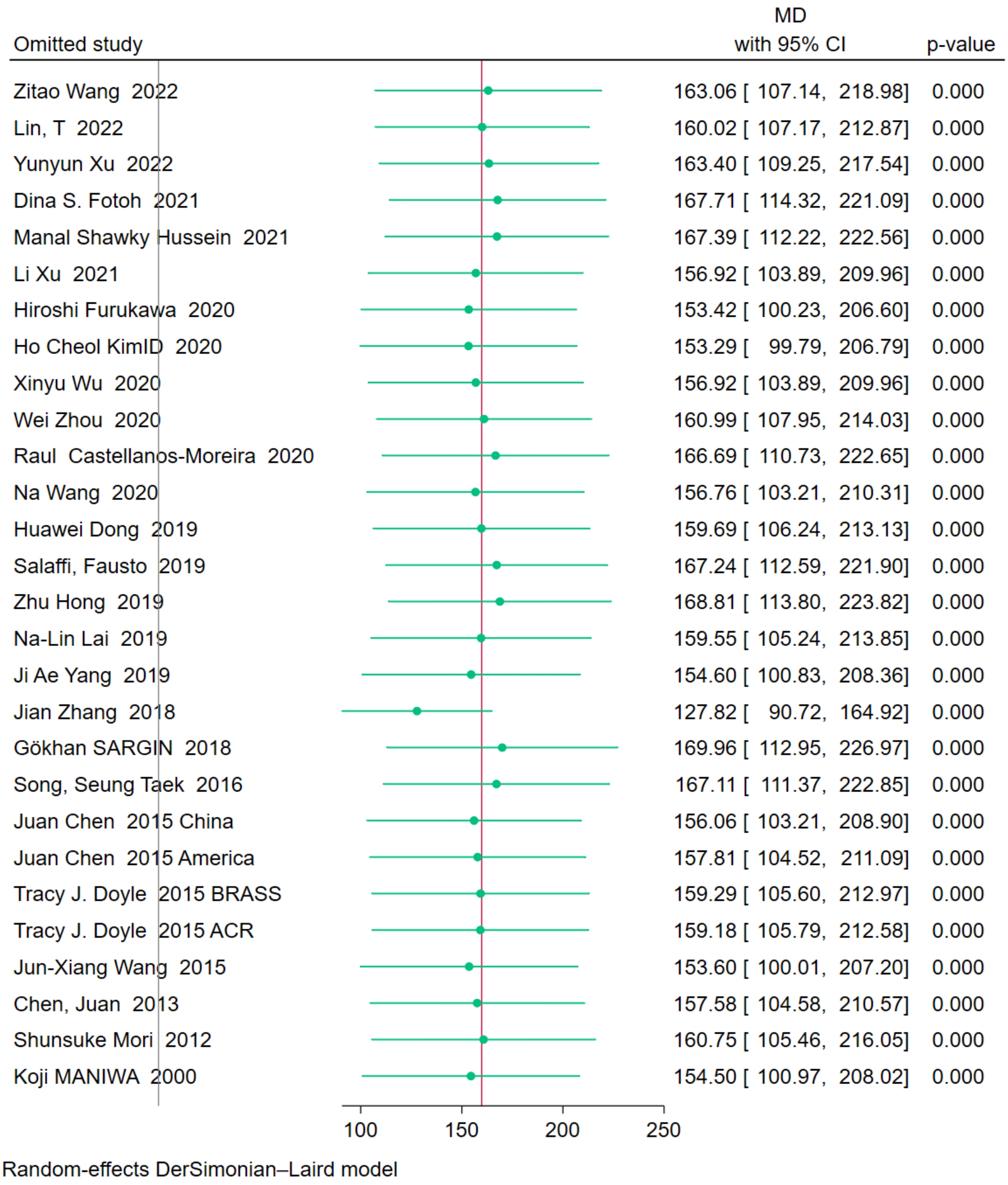

Sensitivity Analysis of RF as a Biomarker for RA-ILD

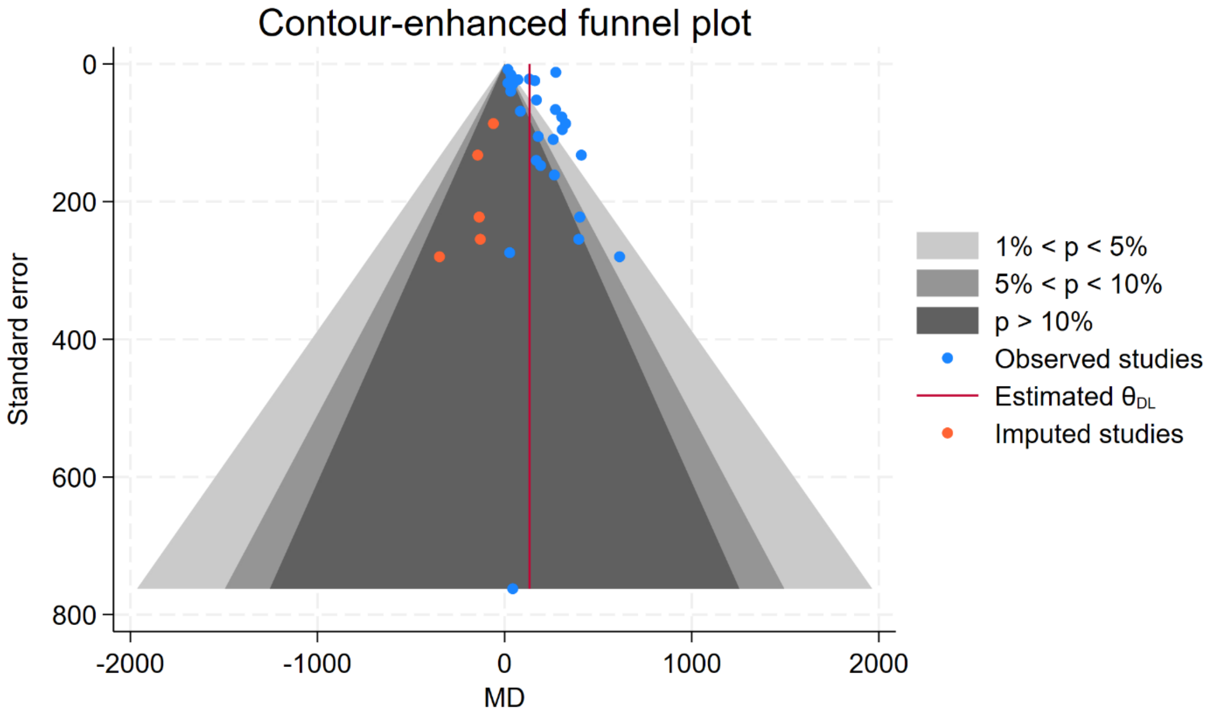

Snip-and-fill funnel plot of RF as a Biomarker for RA-ILD

5 KL-6

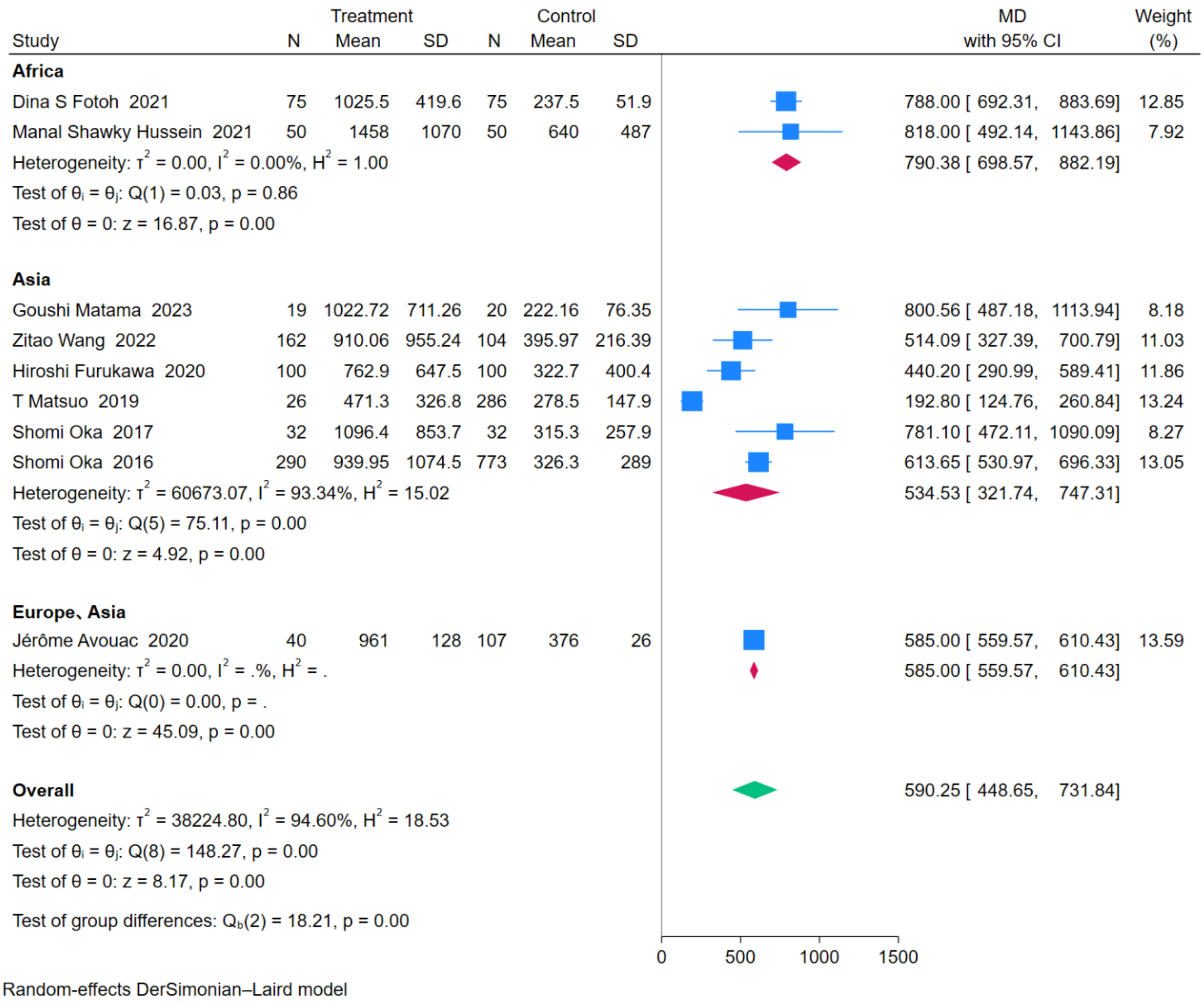

Association Between KL-6 and RA-ILD: Subgroup Analysis by Region ESR

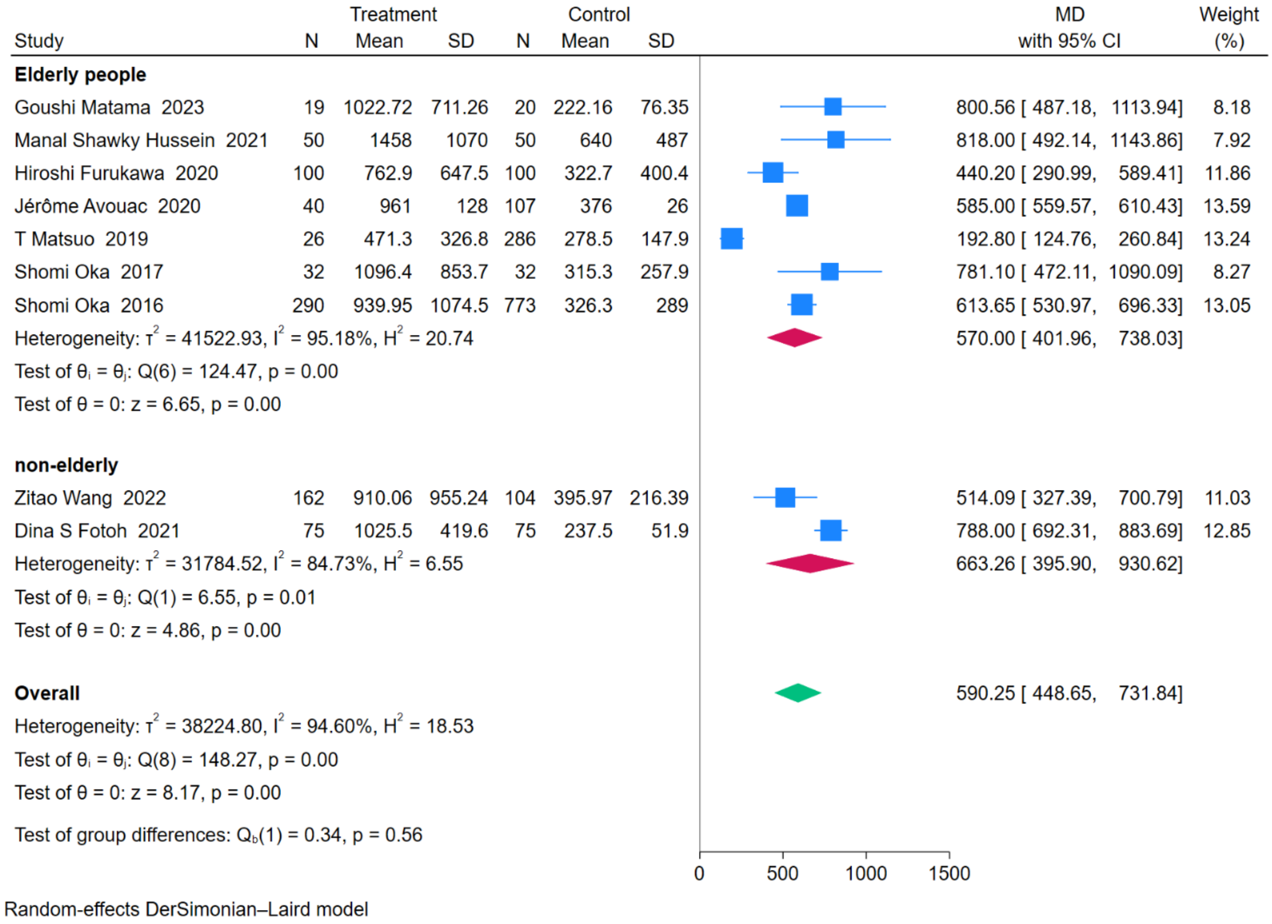

Association Between KL-6 and RA-ILD: Subgroup Analysis by Age

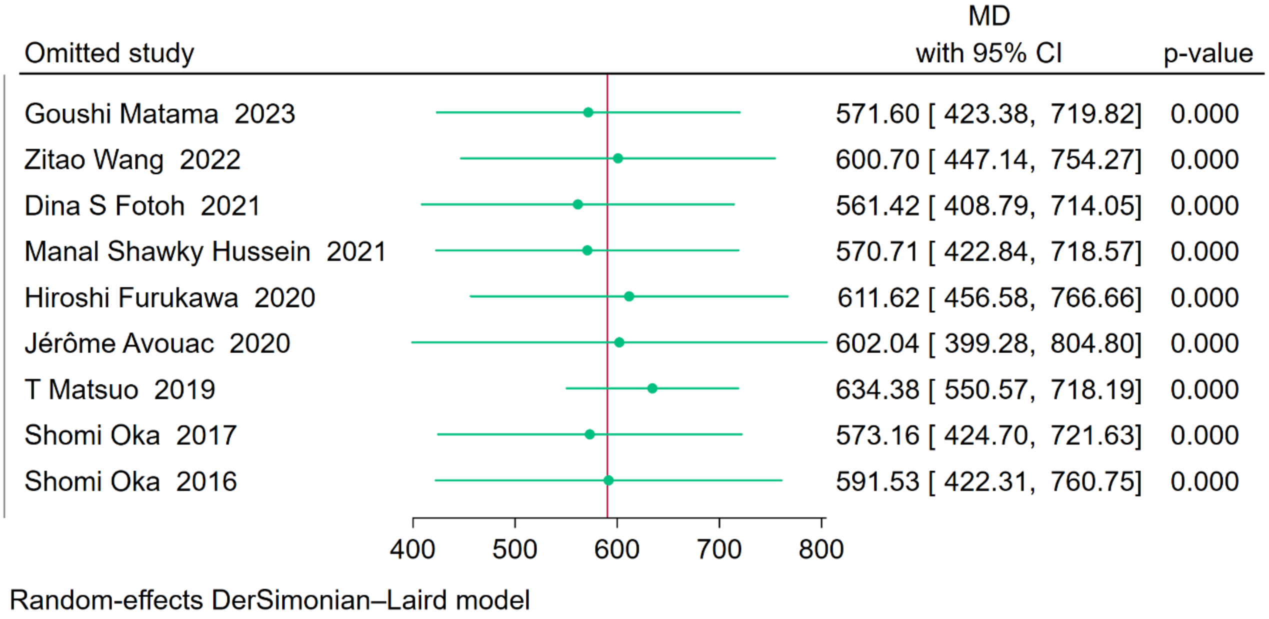

Sensitivity Analysis of KL-6 as a Biomarker for RA-ILD

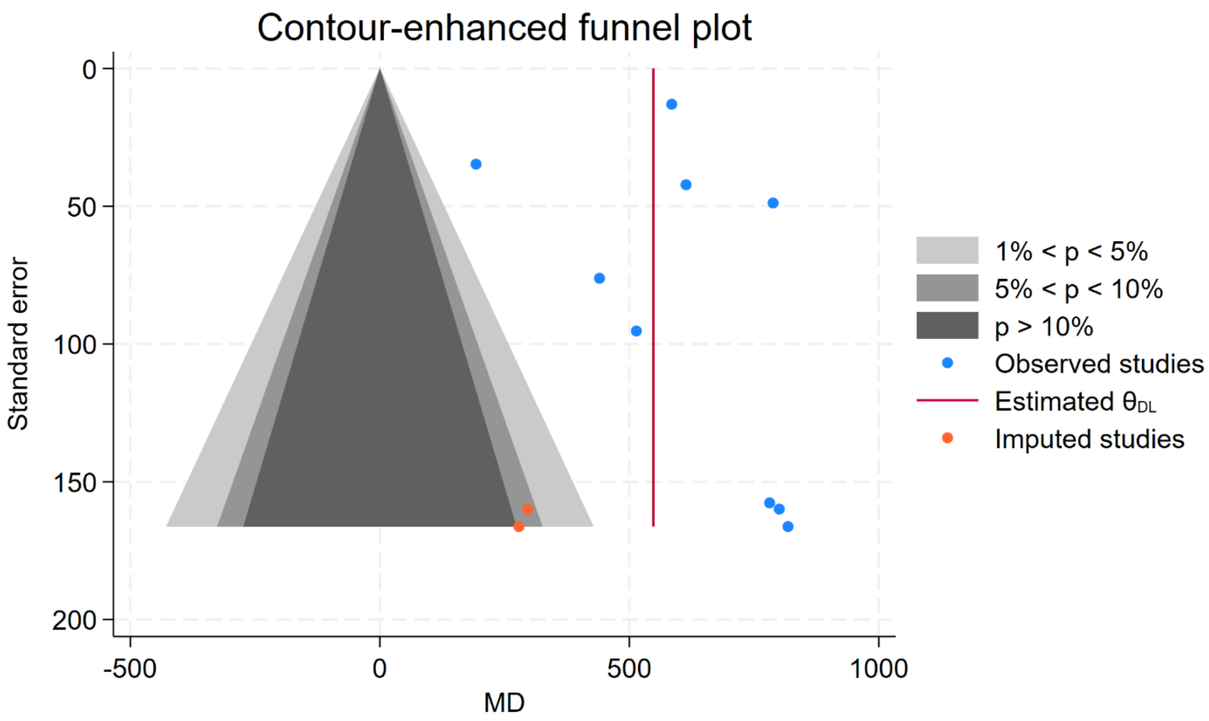

Snip-and-fill funnel plot of KL-6 as a Biomarker for RA-ILD

6 SP-D

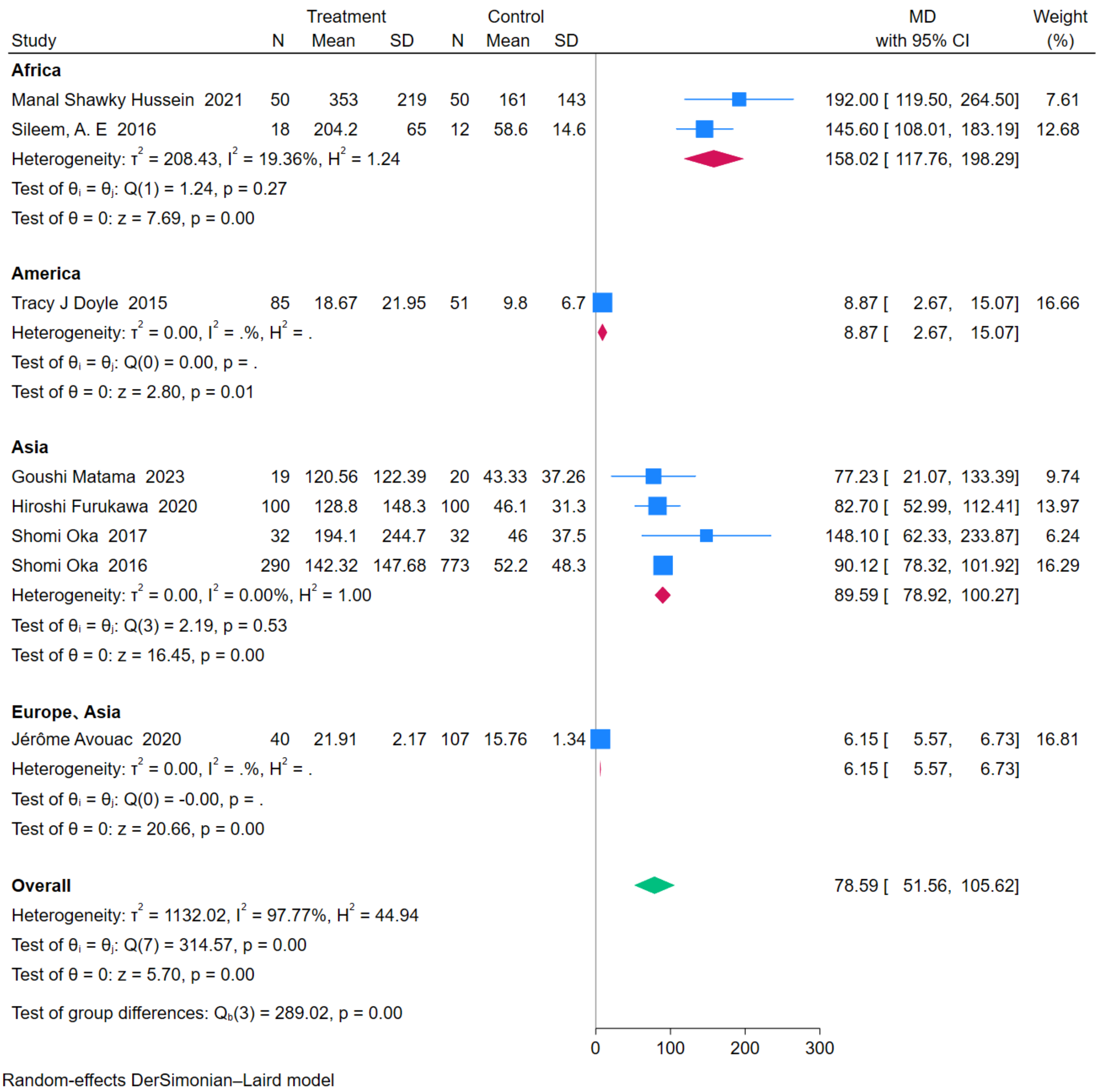

Association Between SP-D and RA-ILD: Subgroup Analysis by Region

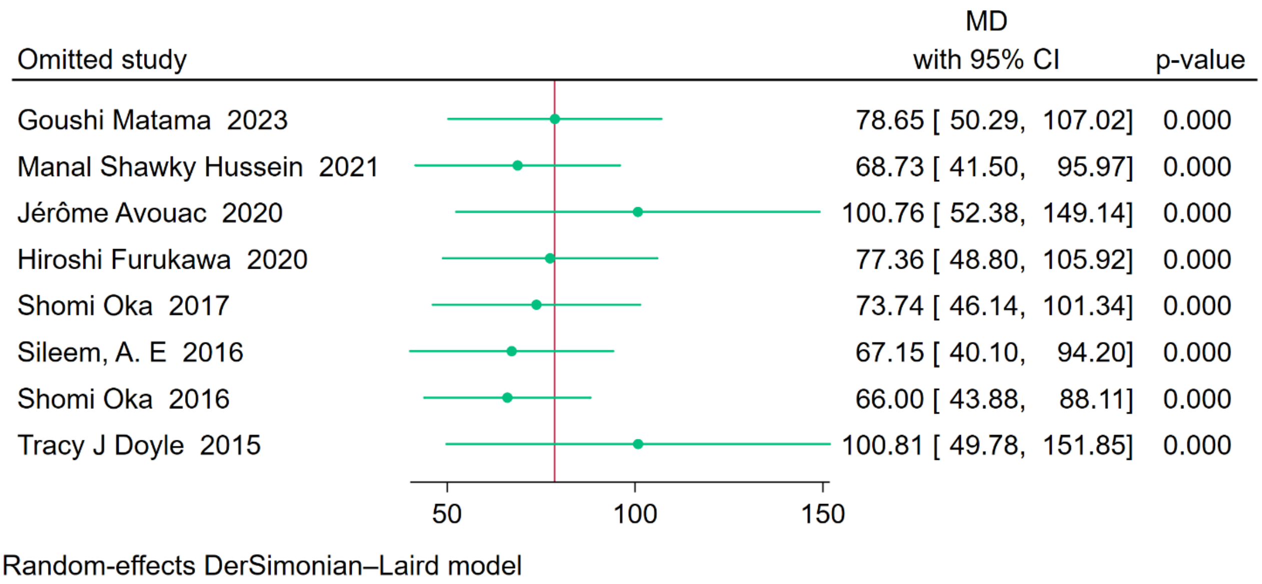

Sensitivity Analysis of SP-D as a Biomarker for RA-ILD

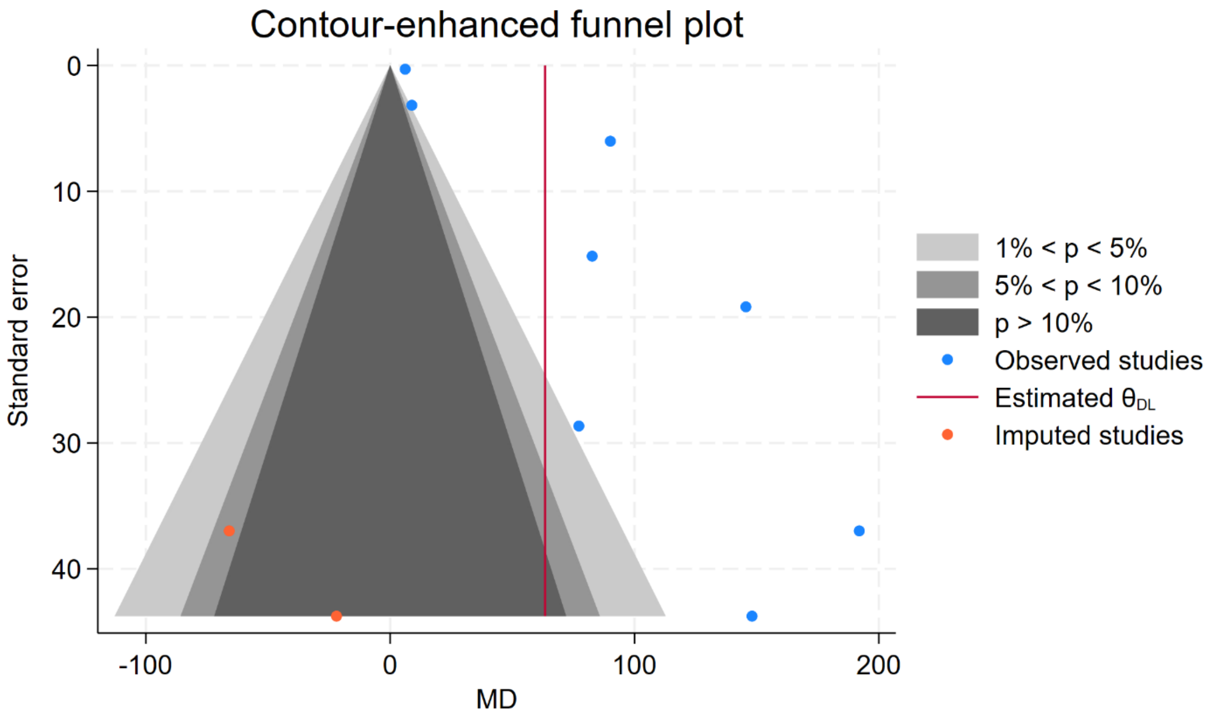

Snip-and-fill funnel plot of SP-D as a Biomarker for RA-ILD

7 CEA

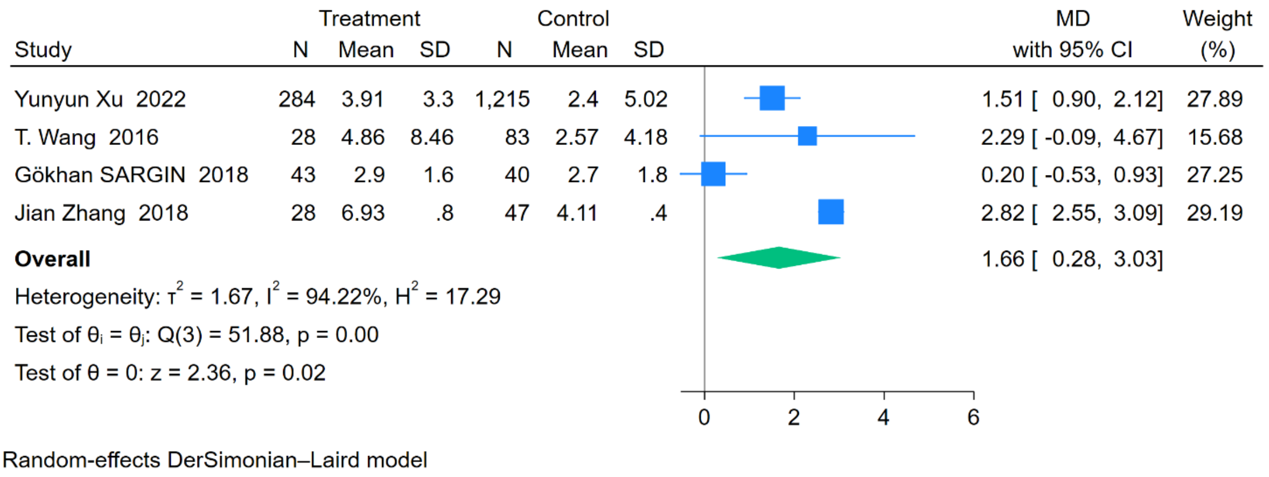

Forest plot for CEA as a Biomarker for RA-ILD

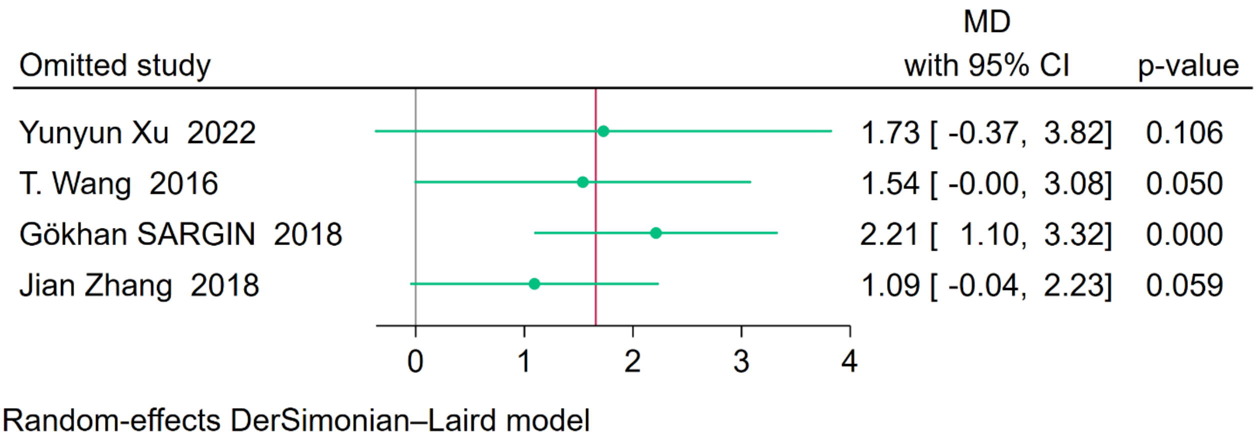

Sensitivity Analysis of CEA as a Biomarker for RA-ILD

8 CA19-9

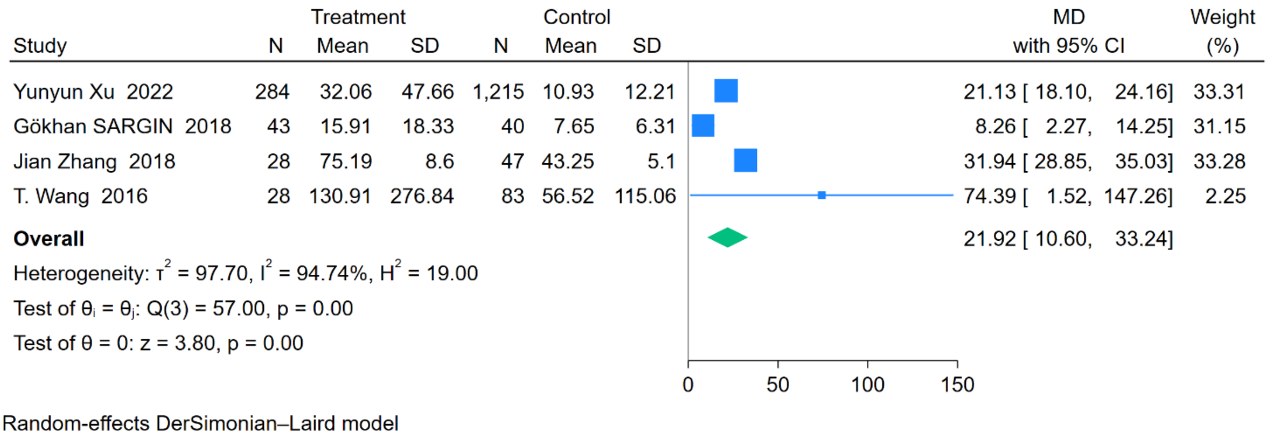

Forest plot for CA19-9 as a Biomarker for RA-ILD

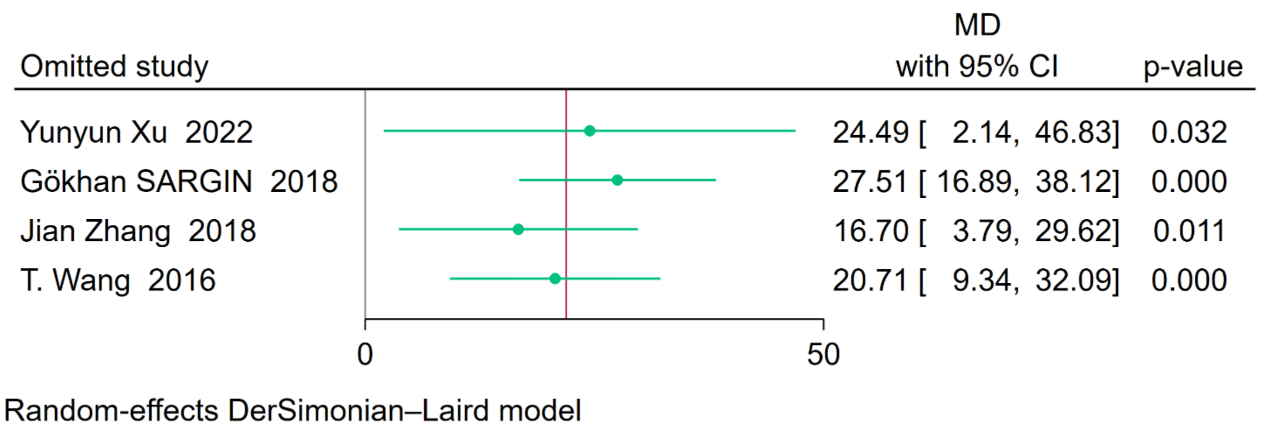

Sensitivity Analysis of CA19-9 as a Biomarker for RA-ILD

9 CA-125

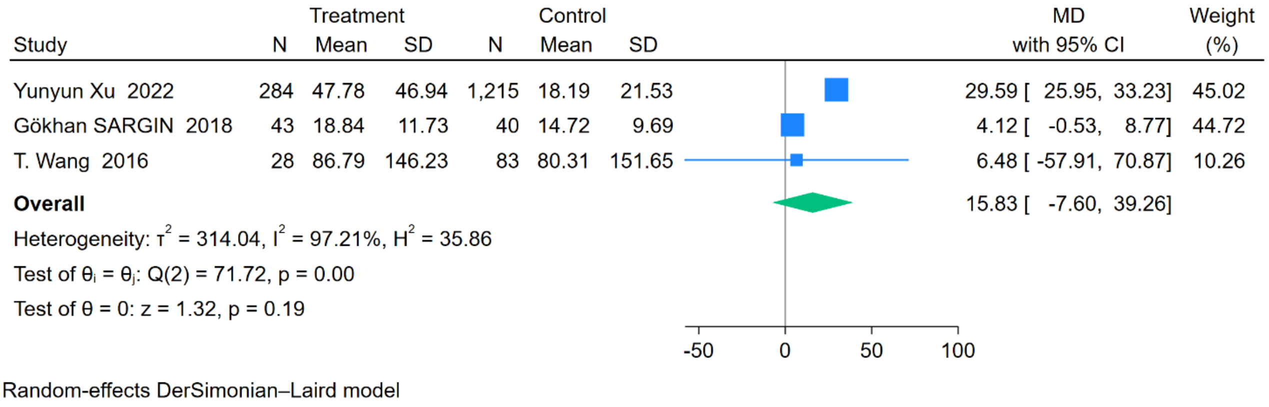

Forest plot for CA-125 as a Biomarker for RA-ILD

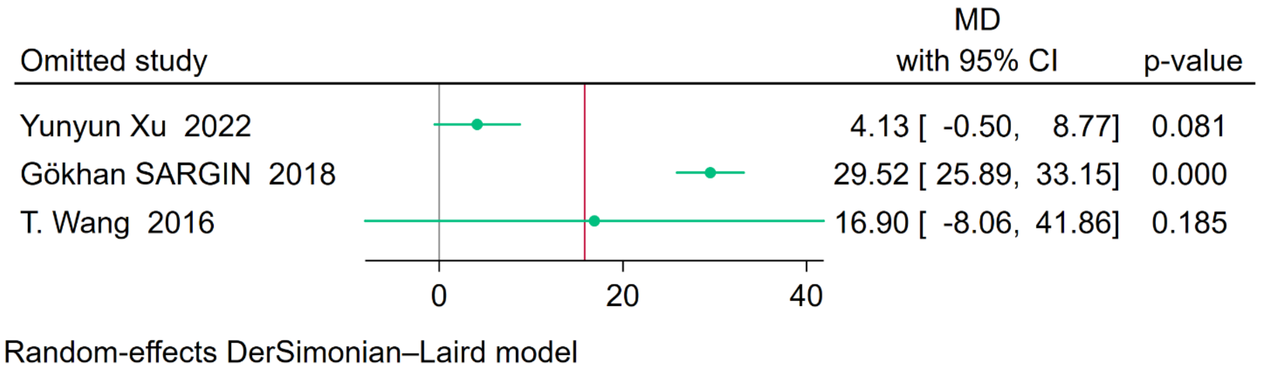

Sensitivity Analysis of CA-125 as a Biomarker for RA-ILD

10 CA-153

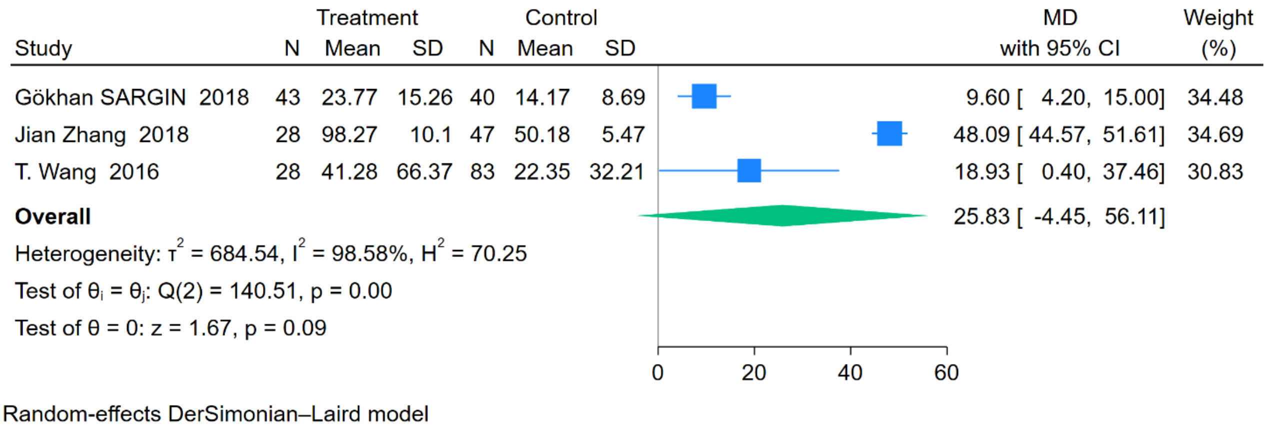

Forest plot for CA-153 as a Biomarker for RA-ILD

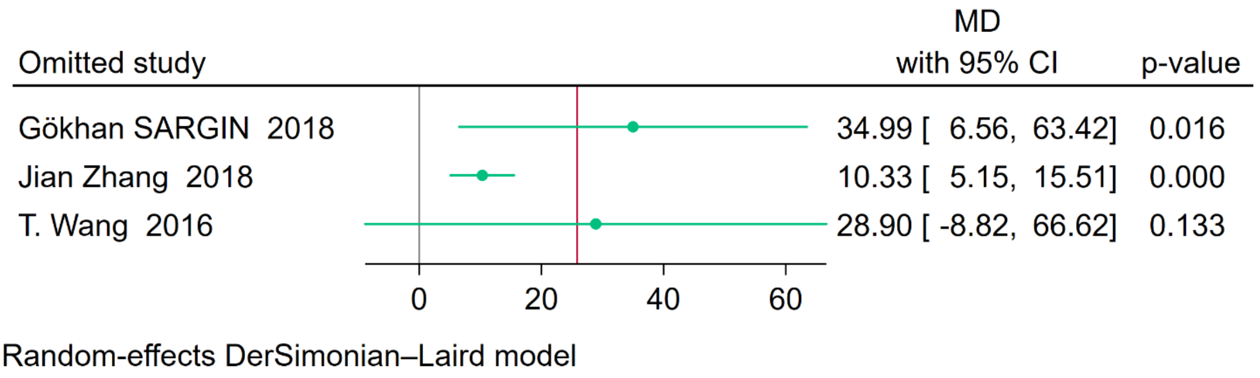

Sensitivity Analysis of CA-153 as a Biomarker for RA-ILD

11 MMP-7

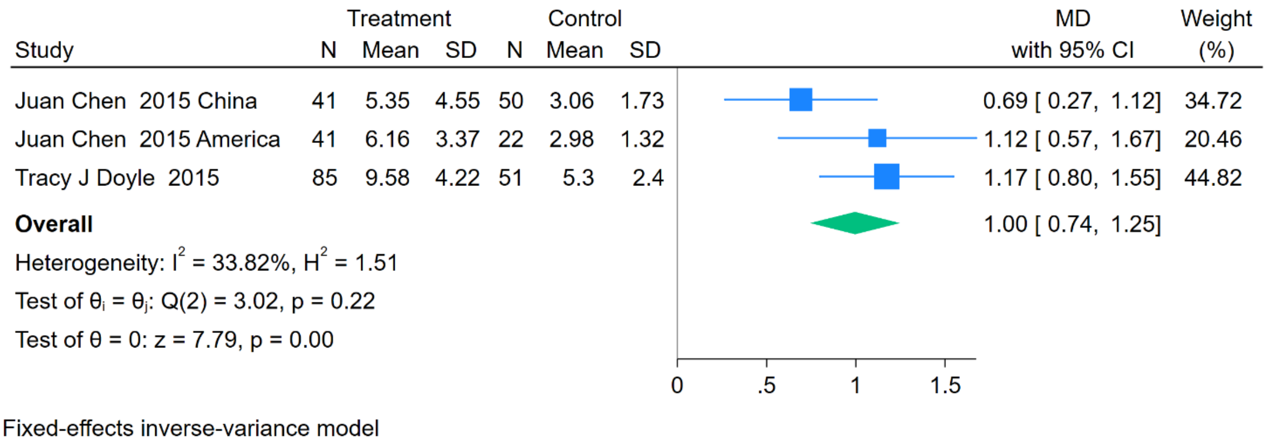

Forest plot for MMP-7 as a Biomarker for RA-ILD

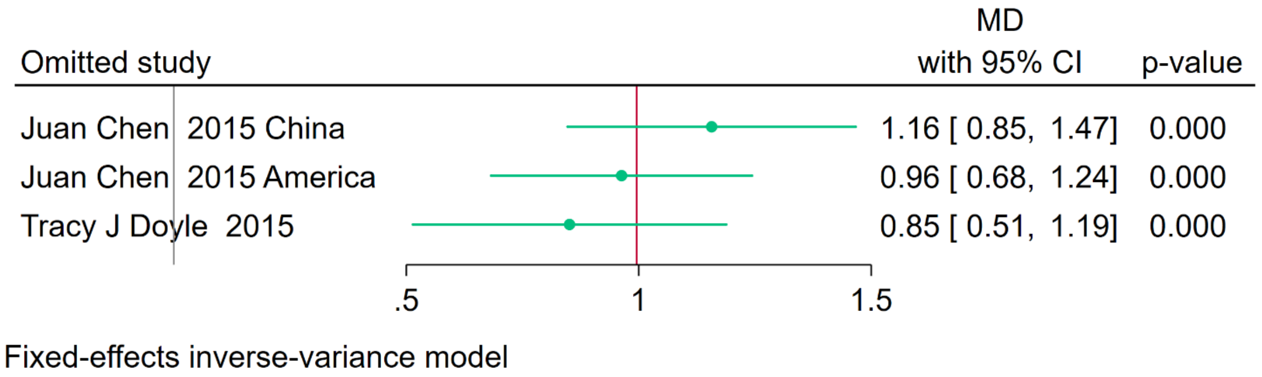

Sensitivity Analysis of MMP-7 as a Biomarker for RA-ILD

12 CXCL-10

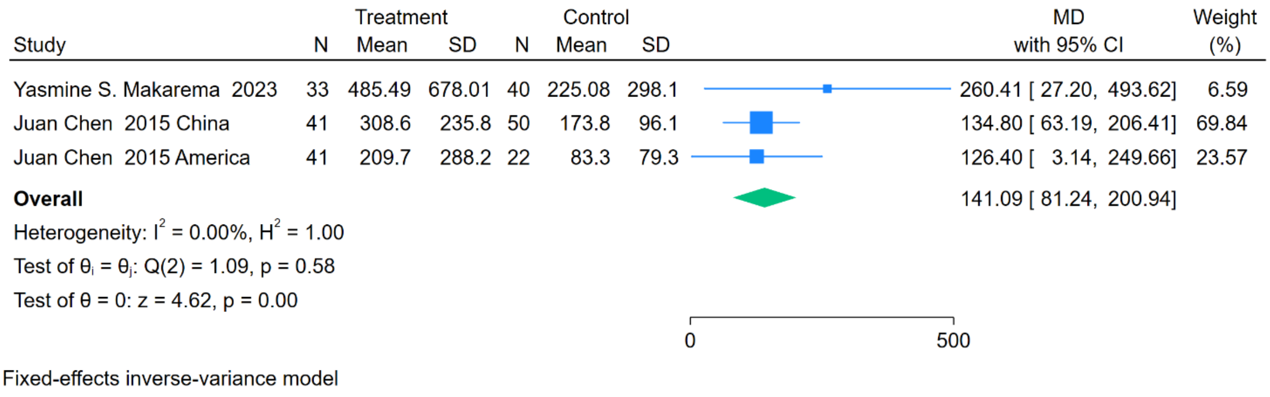

Forest plot for CXCL-10 as a Biomarker for RA-ILD

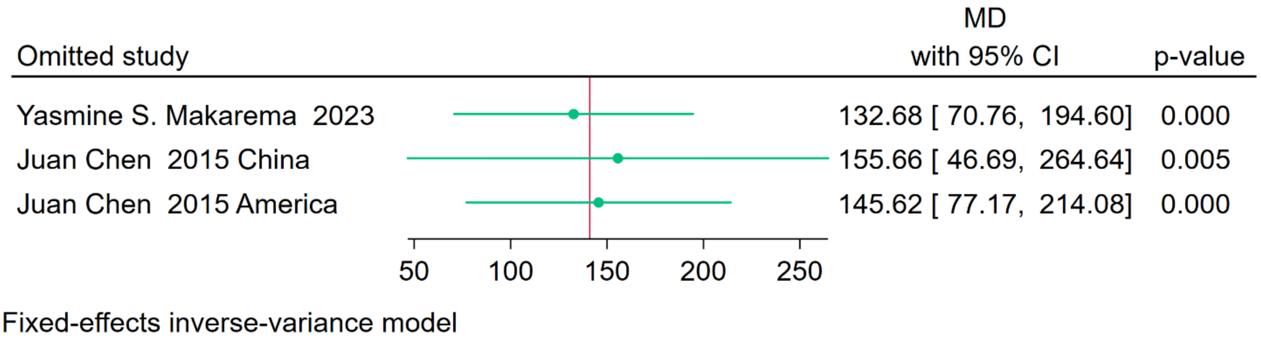

Sensitivity Analysis of CXCL-10 as a Biomarker for RA-ILD

13 PLR

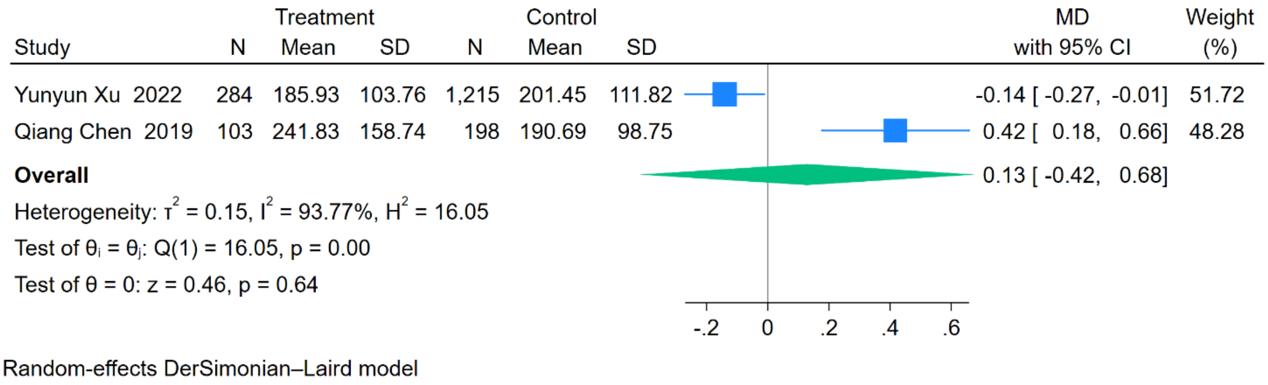

Forest plot for PLR as a Biomarker for RA-ILD

14 NLR

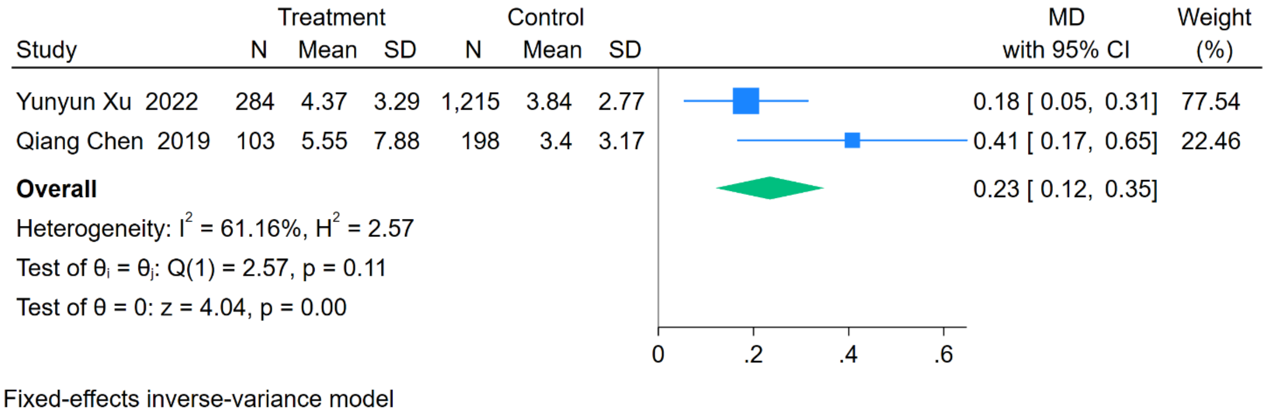

Forest plot for NLR as a Biomarker for RA-ILD

4. **Meta-analysis results of the Severity assessment of RA-ILD**

15 KL-6

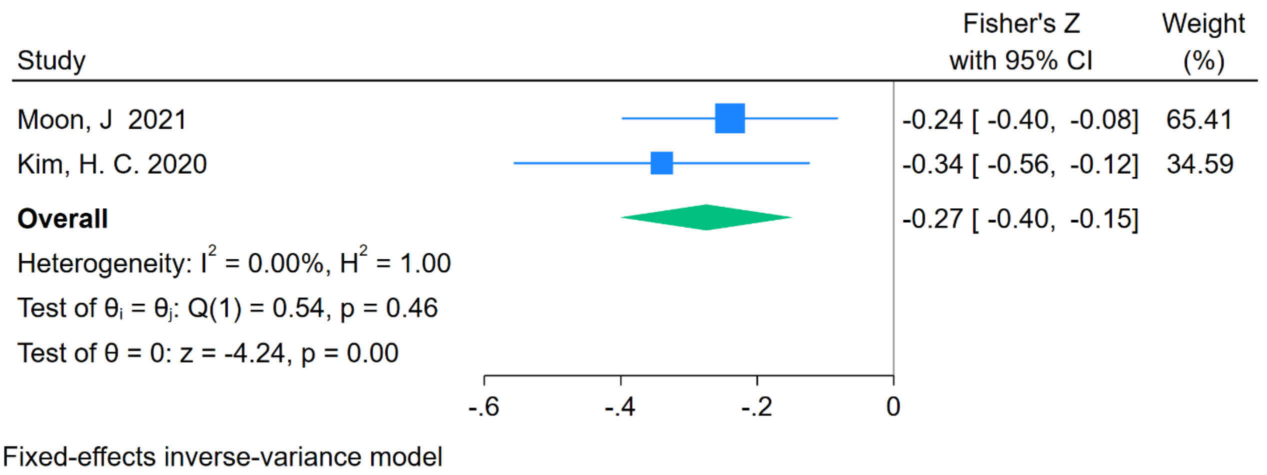

Forest Plot of the Correlation between KL-6 and FVC

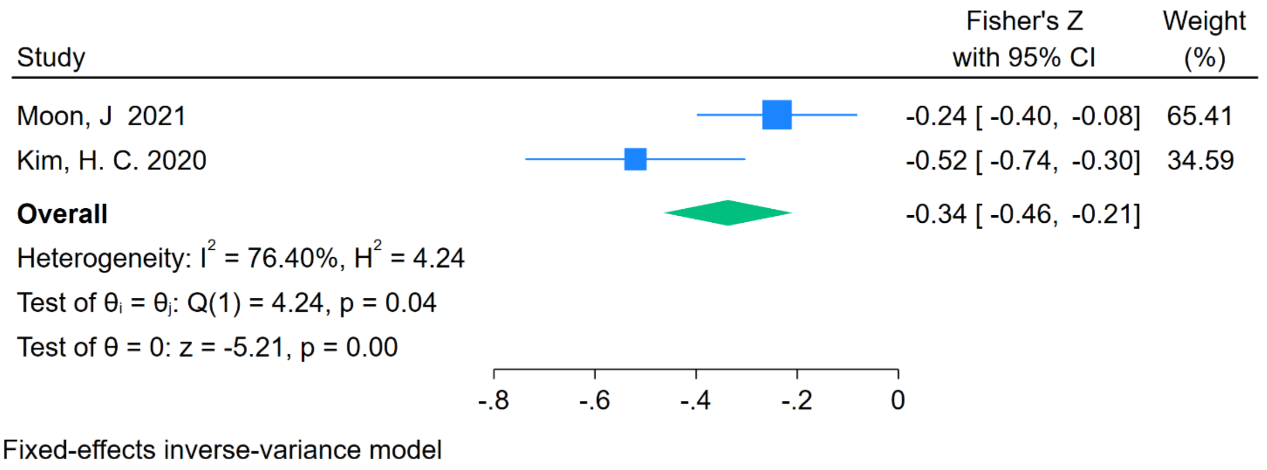

Forest Plot of the Correlation between KL-6 and DLCO

16 MMP-7

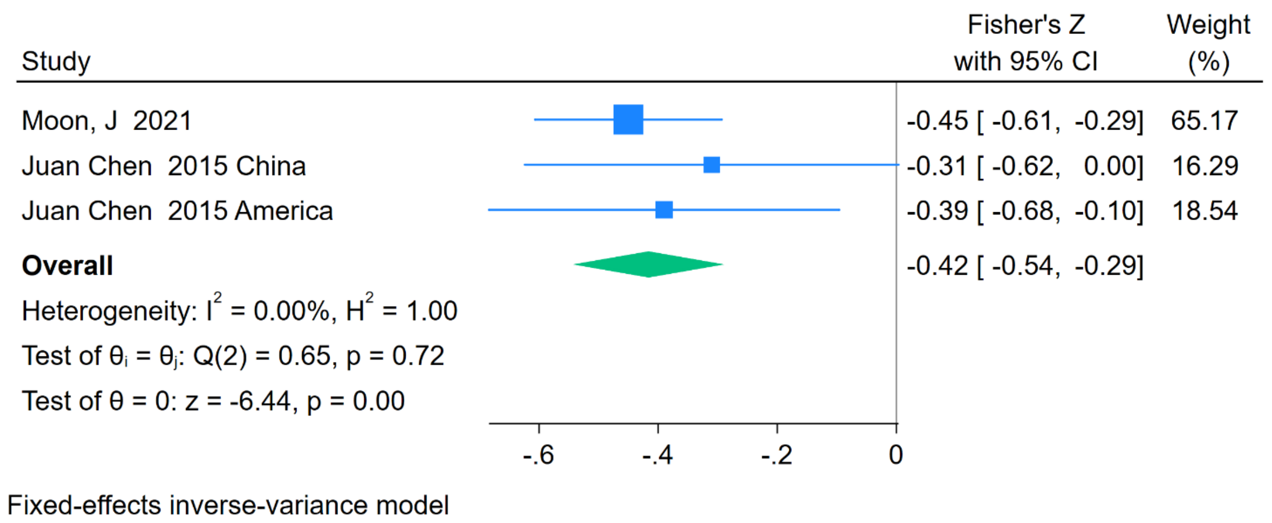

Forest Plot of the Correlation between MMP-7 and DLCO

17 HE4

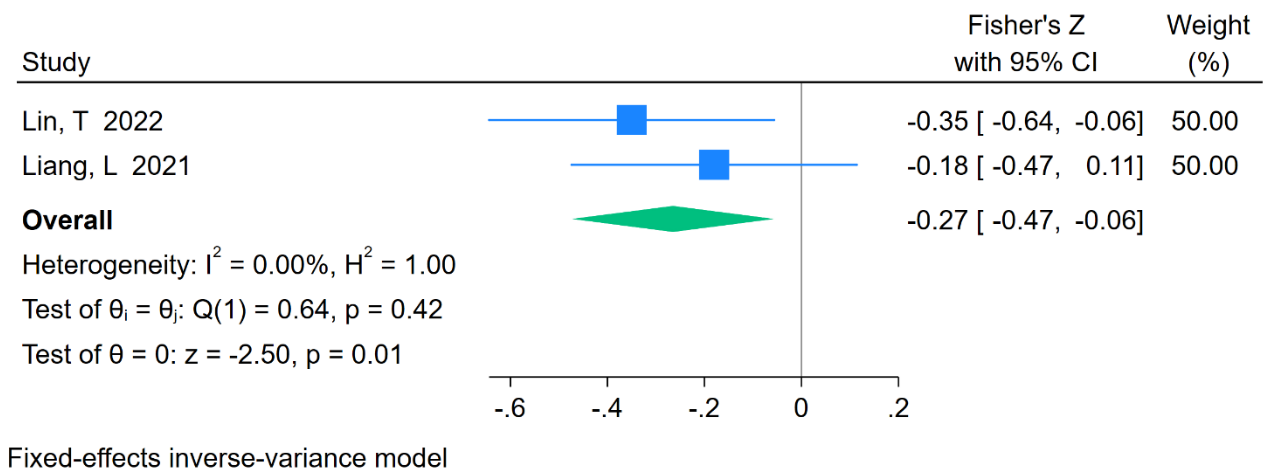

Forest Plot of the Correlation between HE4 and FVC%

5. **Meta-analysis results of the Prognostic assessment of RA-ILD**

18 KL-6

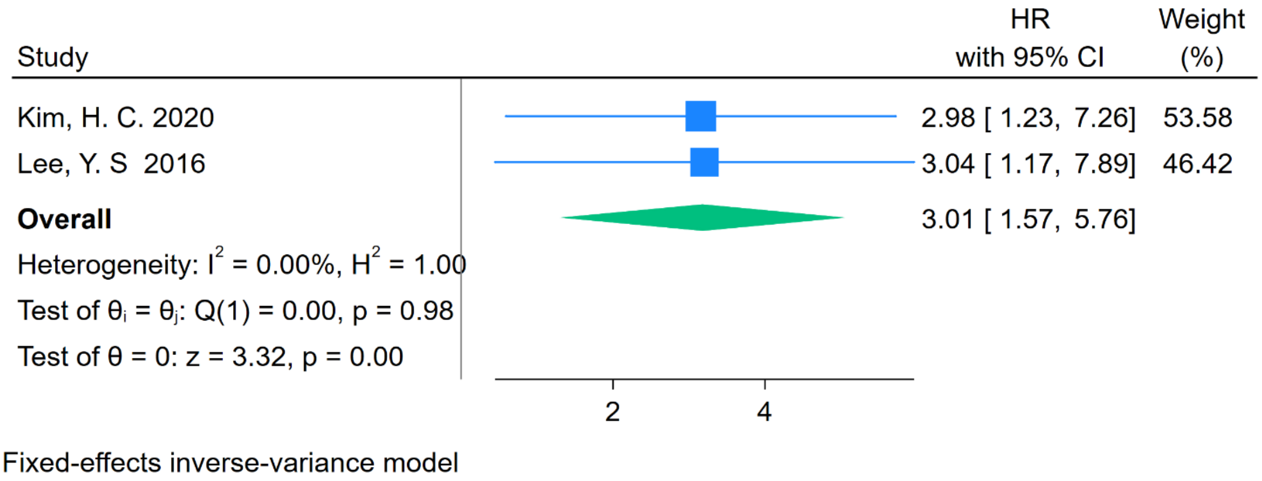

Forest Plot of the Prognostic Relationship between KL-6 and RA-ILD
